# Euglenozoa: taxonomy, diversity and ecology, symbioses and viruses

**DOI:** 10.1098/rsob.200407

**Published:** 2021-03-10

**Authors:** Alexei Y. Kostygov, Anna Karnkowska, Jan Votýpka, Daria Tashyreva, Kacper Maciszewski, Vyacheslav Yurchenko, Julius Lukeš

**Affiliations:** ^1^ Life Science Research Centre, Faculty of Science, University of Ostrava, Ostrava, Czech Republic; ^2^ Zoological Institute, Russian Academy of Sciences, St Petersburg, Russia; ^3^ Institute of Evolutionary Biology, Faculty of Biology, Biological and Chemical Research Centre, University of Warsaw, Warsaw, Poland; ^4^ Institute of Parasitology, Czech Academy of Sciences, České Budějovice (Budweis), Czech Republic; ^5^ Department of Parasitology, Faculty of Science, Charles University, Prague, Czech Republic; ^6^ Martsinovsky Institute of Medical Parasitology, Tropical and Vector Borne Diseases, Sechenov University, Moscow, Russia; ^7^ Faculty of Sciences, University of South Bohemia, České Budějovice (Budweis), Czech Republic

**Keywords:** Euglenida, Kinetoplastida, Diplonemida, microbial eukaryotes, systematics, phylogeny

## Abstract

Euglenozoa is a species-rich group of protists, which have extremely diverse lifestyles and a range of features that distinguish them from other eukaryotes. They are composed of free-living and parasitic kinetoplastids, mostly free-living diplonemids, heterotrophic and photosynthetic euglenids, as well as deep-sea symbiontids. Although they form a well-supported monophyletic group, these morphologically rather distinct groups are almost never treated together in a comparative manner, as attempted here. We present an updated taxonomy, complemented by photos of representative species, with notes on diversity, distribution and biology of euglenozoans. For kinetoplastids, we propose a significantly modified taxonomy that reflects the latest findings. Finally, we summarize what is known about viruses infecting euglenozoans, as well as their relationships with ecto- and endosymbiotic bacteria.

## Graphical abstract

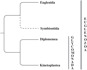

## 1. Introduction

It is generally accepted that Euglenozoa belong to the most unusual eukaryotes [1–3]. This is based on a substantial body of evidence showing that in a number of cellular processes and structures, these almost invariably mono- or bi-flagellated protists departed from what can be considered the ‘eukaryotic consensus'. However, this consensus was defined by the studies of just a handful of model organisms, most of which are multicellular [4]. Hence, since the majority of the extant eukaryotic diversity is hidden in protists [5], we prefer to use a ‘protist-centric’ view, which postulates that these unicellular forms actually are the eukaryotic standard, while the other lineages represent departures from the norm.

The phylum Euglenozoa splits into three well-defined lineages—euglenids, kinetoplastids and diplonemids—with different life strategies and distinct morphologies, yet still unified by a number of common features [6]. Although the euglenids are sometimes further subdivided into Euglenida and Symbiontida [3], both groups are usually treated together due to their morphological similarity, and we still cannot compare their genomic features in the absence of such data from the latter taxon [7]. A recent multigene phylogenetic reconstruction pointed to the potentially sister relationship between Symbiontida and Glycomonada (Kinetoplastea + Diplonemea) [8], suggesting that Symbiontida may become a separate group when more data become available (tree A).

**Tree A.**
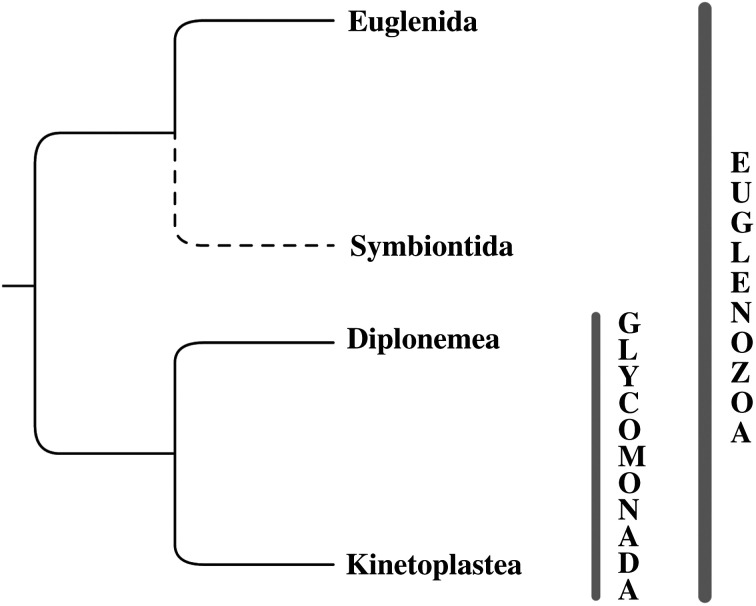
Euglenozoa. A consensus tree based on multiple phylogenetic reconstructions showing relationships among major clades. The unstable position of Symbiontida is marked with a dotted line and further described in the section on euglenid taxonomy.

Apart from summarizing taxonomic works, the euglenozoans are almost never treated together in the literature. The kinetoplastid flagellates are by far the best-studied representatives (almost exclusively from a parasitology-centric perspective), with most attention given to the causative agents of serious diseases, such as sleeping sickness, Chagas disease and leishmaniases [9,10]. The diplonemids, as detailed below, were considered a marginal group with no ecological relevance. That has changed recently [11,12], but still very few molecular data other than 18S rRNA are available for this almost exclusively marine group. Finally, the photosynthetic and heterotrophic euglenids are ecologically significant, primarily in freshwater ecosystems, and have potential in biotechnologies [2,13].

The striking differences in lifestyles and cellular (ultra)structure obscure the significant similarities in basic molecular processes. Firstly, all these groups distinguish themselves from other eukaryotes by transcribing nuclear genes in a polycistronic manner [14]. In neither case are the co-transcribed genes functionally related, which distinguishes them from the prokaryotic operons. The usually very long polycistronic mRNA is subsequently processed into monomeric transcripts, which are subject to another process that is found in eukaryotes rather infrequently—*trans*-splicing. At the 5′ end of each monocistronic mRNA, short spliced leader (SL) RNA, already equipped with a methylated cap, becomes attached. The corresponding SL RNA gene is invariably multicopy, and highly conserved, yet with minor species-specific differences [15].

The similarities do not stop there. In their single or dual flagella, all euglenozoans evolved an extra-axonemal structure termed the paraflagellar rod, which supports their flagella [16]. The paraflagellar rod has a characteristic lattice-like structure, which is composed of dozens of proteins, phylogenetically restricted to euglenozoans. It is reduced only in the endosymbiont-containing trypanosomatids and the amastigotes of *Leishmania* [17]. Studied so far only in kinetoplastids, the paraflagellar rod not only increases propulsion of the cell [18], but also participates in morphogenetic and metabolic roles, as well as in environmental sensing [19]. While all these synapomorphies were probably present in the euglenozoan common ancestor, euglenids, diplonemids and kinetoplastids have acquired significant differences over the course of evolution. This is particularly striking in the case of *cis*-splicing, since spliceosomal introns are almost absent in the latter group [20], while they are abundantly present in euglenids and diplonemids, many being seemingly non-canonical [11,21]. Another clear difference rests in the size of both nuclear and mitochondrial genomes. The dearth of high-quality data for nuclear genomes of euglenids and their absence in the case of diplonemids are due to the large size and repetitive character of the latter. The transcriptomes from both groups contain an extremely high number of protein-coding genes, probably reflecting their metabolic versatility [6,13]. The situation is quite different in kinetoplastids, the parasitic lifestyle of which led to gene reduction and streamlining [6]. Moreover, due to their small and compact genomes, they belong to the most sequenced eukaryotes [22].

Unexpected differences among the main euglenozoan lineages recently became apparent for their mitochondrial genomes and transcriptomes. Kinetoplastids harbour in their mitochondrial DNA in the form of relaxed (rarely supercoiled) circular molecules, either catenated or free, of two types—maxicircles and minicircles, with the former carrying all protein-coding genes, while the latter encode guide RNA genes required for the editing of the maxicircle transcripts [23]. The size of maxicircles is rather uniform, while the minicircles come in different variants [24]. In diplonemids, the single type of non-catenated circles uniquely encodes fragments of protein-coding genes, the transcripts of which have to be massively *trans*-spliced and edited in order to become translatable [25]. However, in both groups, the mitochondrial DNA is inflated, and its transcripts are extensively edited [26]. This contrasts with euglenids that lack any form of editing in their mitochondrion, which also contains a small genome composed of heavily fragmented linear molecules [27]. Probably, the most important difference among these groups is the presence of a secondary green plastid solely in euglenids, which have acquired it after their divergence from other euglenozoans [2,28].

Until recently, our knowledge of different groups within euglenozoans was much influenced by the availability of full-size nuclear genome sequences. While hundreds of high-quality genomes are available for trypanosomatids [22], only one such genome is available for bodonids [29] and euglenids [13], respectively, and none for diplonemids. However, this is bound to change soon, mostly due to the ever-decreasing costs and improving sequencing technologies. Recent comparative analyses of molecular features among kinetoplastids, euglenids and diplonemids were based on transcriptomes available for all of them [30].

Future studies of euglenozoans will be heavily influenced by the accessibility of their representatives to (efficient) genetic manipulations. The amenability of trypanosomatids to a range of genetic tools turned them into arguably the functionally best-studied protists [31], while most other groups significantly lag behind. However, this unfavourable situation has changed recently, as first reports of genetic modifications of bodonids, diplonemids and euglenids have been published [32–36]. Anticipated improvement of the methodologies of forward and reverse genetics, which would allow medium- or high-throughput functional analyses in these taxonomic groups, almost guarantee major discoveries.

Euglenozoa is a very peculiar group, encompassing organisms strikingly dissimilar in their ecology, ranging from autotrophy to obligate parasitism. This inevitably influenced their classification in the era of the two-kingdoms-of-life paradigm. Kinetoplastids and diplonemids were historically considered predominantly as protozoa, and thus the International Code of Zoological Nomenclature (ICZN) was used for their nomenclature, while euglenids have been classified by different authors as either protozoa or algae. This ambiguity is reflected in their nomenclature, which has been governed in parallel by the ICZN as well as the International Code of Botanical Nomenclature (ICBN) and the International Code of Nomenclature for algae, fungi and plants (ICN), which replaced the latter in 2011. Apart from formal differences, such as the rules on citing authorship of names and emendations, this led to significant issues that include certain taxa having different names depending on the selected system (zoological or botanical). This concerns names of family-group taxa, which have different suffixes depending on the system and, more importantly, names of genera, which may be valid according to one code, but regarded as junior homonyms, and therefore replaced with different names. In addition, the ICZN jurisdiction does not extend above the family-group level, whereas ICN does not have such a restriction. Here, the nomenclature of kinetoplastids and diplonemids follows the ICZN, while for some euglenid groups, the ICN is used by default with the valid names according to the ICZN indicated.

## 2. Kinetoplastea

### 2.1. Biology

#### 2.1.1. Free-living kinetoplastids

The common ancestor of Kinetoplastea apparently was a free-living benthic bacterivorous organism using the anterior flagellum for motion and transporting food particles to the cytostome, while the posterior one ensured gliding on the substrate (plate A, 1,2,4–10,12–14). This lifestyle is still preserved by a large proportion of kinetoplastids [37]. They inhabit permanent and temporary water bodies with various levels of salinity (freshwater to hypersaline) and some species were shown to tolerate the transition from marine water to freshwater, and *vice versa* [38–41]. Kinetoplastids are very numerous in benthic communities, where they constitute 5–20% of the total biomass of all heterotrophic flagellates, second only to euglenids, suggesting their important role in controlling bacterial growth [42]. They are abundant in seawater ice and some species can be cultured even from the pelagic zone, demonstrating their presence there at least at the cystic stage [43,44]. Recent studies using molecular methods demonstrated that in many water bodies, most of the kinetoplastid biomass is created by neobodonids, of which *Neobodo, Rhynchomonas* and *Dimastigella* are the most frequent ones (plate A, 7,9,14) [45–47]. An extensive analysis of free-living kinetoplastids in hundreds of globally collected oceanic samples revealed their abundance being 0.14%, with highest abundance in the mesopelagic zone. Their community structure and richness are significantly influenced by oxygen concentration, salinity and temperature [48].

**Plate A.**
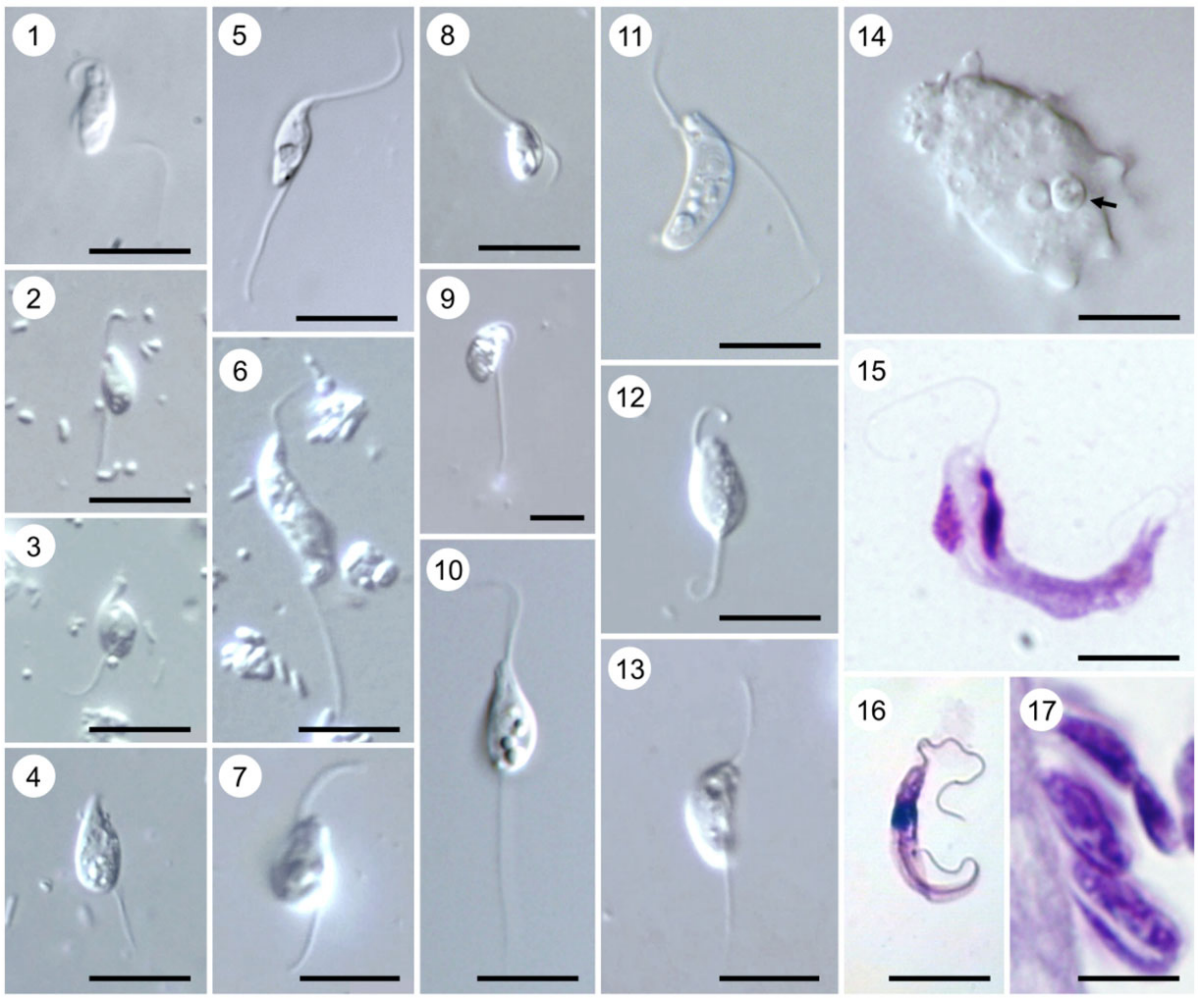
Bodonids. Light micrographs of cultured (1) *Actuariola framvarensis* (provided by Thorsten Stoeck); (2) *Neobodo curvifilus* (provided by Kristina Prokina and Denis Tikhonenkov); (3) *Rhynchomonas nasuta* (provided by Kristina Prokina and Denis Tikhonenkov); (4) *Rhynchobodo* sp. (provided by Kristina Prokina and Denis Tikhonenkov); (5) *Azumiobodo hoyamushi* (provided by Shinichi Kitamura and Euichi Hirose); (6) *Dimastigella mimosa* (provided by Kristina Prokina and Denis Tikhonenkov); (7) *Bordnamonas tropicana* (provided by Kristina Prokina and Denis Tikhonenkov); (8) *Klosteria bodomorphis* (provided by Kristina Prokina and Denis Tikhonenkov); (9) *Bodo saltans* (provided by Kristina Prokina and Denis Tikhonenkov); (10) *Cruzella marina*; (11) *Allobodo chlorophagus* (provided by Alastair Simpson and Yana Eglit); (12) *Procryptobia sorokini* (provided by Kristina Prokina and Denis Tikhonenkov); (13) *Parabodo caudatus* (provided by Kristina Prokina and Denis Tikhonenkov); (14) *Perkinsela* sp. (arrow indicates its position inside *Paramoeba pemaquidensis*) (provided by Ivan Fiala); (15) Giemsa-stained *Trypanoplasma borreli*; (16) *Cryptobia vaginalis* (provided by Marina N. Malysheva); (17) Toluidine-stained semi-thin section of fish gill with attached *Ichthyobodo necator* (provided by Iva Dyková). Scale bar, 10 µm (1–8; 10–17); 5 µm (9).

Moreover, many kinetoplastids live in the soil and readily settle on various organic substrates, such as faeces, composts, etc. [49,50]. Most of the free-living flagellates graze on bacteria with the help of cytostomal lips or, as in Rhynchomonadidae, a flagellum-attached motile proboscis. In addition to bacterial cells, their digestive vacuoles can contain microalgae or detritus particles. *Parabodo caudatus* (plate A, 13), being a relatively large (up to 20 µm long) species, exerts both bacterivory and predation, while *Rhynchobodo* spp. (plate A, 4) are obligatory predators devouring other flagellates [37].

Some kinetoplastids are known to form cysts, which help them to survive adverse conditions, for example, pass through the digestive system of an animal and settle in its faeces after their discharge [37]. Moreover, some of these flagellates become very tolerant to harsh environments even at the active (non-encysted) stage. This resulted in a series of records of free-living kinetoplastids (*Parabodo caudatus, Dimastigella trypaniformis* and *Procryptobia tremulans*) from stool and urine samples or urine-impregnated animal cage bedding sometimes misinterpreted as evidence of their parasitic nature [51–54]. Interestingly, such tolerance in parabodonids apparently preadapted them to parasitism, which originated in this group at least twice [55].

#### 2.1.2. Parasitic, mutualistic and commensal non-trypanosomatid kinetoplastids

Multiple transitions to various forms of symbiosis can be observed in all orders of Kinetoplastea except Eubodonida (tree B). The earliest branch within this group, Prokinetoplastida, does not contain any described free-living forms. The ectoparasitic *Ichthyobodo*, affecting both freshwater and marine fish, is generally similar to free-living kinetoplastids (plate A, 17). In contrast with other symbiotic forms, it anchors on the host epithelium with its rostrum forming an attachment disc and a cytostome process, which is inserted directly into the cytoplasm of the host cell and feeds by myzocytosis [56]. Accumulation of parasites on the epithelium of gills and fins leads to tissue necrosis, which often entails the death of fish, especially fingerlings. Dissemination of *Ichthyobodo* spp. occurs using a free-swimming stage lacking the rostrum [57].

Another prokinetoplastid genus, *Perkinsela*, represents one of the most simplified symbiotic eukaryotes, permanently living in the cytoplasm of amoebae, such as *Paramoeba* (plate A, 14) [58]. The nature of their relationships is mutualistic as judged by reciprocal metabolic dependence of the two partners, evident from the study of their genomes [59]. Since both *Ichthyobodo* and *Paramoeba* live on fish gills, it was proposed that an ancestral *Ichthyobodo*-like flagellate had been engulfed, but not digested, by an amoeba, and eventually evolved into the endosymbiont *Perkinsela* [58]. *Perkinsela* is tightly associated with cosmopolitan *Paramoeba*, lacks any traces of flagellum (plate A, 14), and has an extremely reduced metabolism, as well as the largest known mitochondrial DNA [59].

The currently unclassified flagellate *Desmomonas prorhynchi*, a parasite of the turbellarian *Prorhynchus*, shares two features with *Ichthyobodo*: polykinetoplast DNA and attachment to the host cell by an appendage at the anterior end. However, this structure performs an exclusively mechanical function, while feeding is supposed to occur via osmotrophy [60]. Another flagellate with an uncertain taxonomic position, *Cephalothamnium cyclopum*, is the only described colonial kinetoplastid, which attaches to freshwater copepods [61]. Like its free-living relatives, this flagellate feeds by intercepting bacterial cells with its anterior flagellum and directing them to cytostomal opening. Given that *C. cyclopum* uses its host only as a substrate and the only inconvenience from its presence may consist of decreased hydrodynamic characteristics of the crustacean, this kinetoplastid is considered as ectocommensal.

*Azumiobodo hoyamushi* is a neobodonid parasite of ascidians, of which the most important is the sea pineapple *Halocynthia roretzi*, a cultivated edible species popular in Korea and Japan (plate A, 5). By invading the tunic, this flagellate is responsible for the so-called soft tunic syndrome, associated with high mortality rates [62]. Being seasonal, it survives the period of high temperatures in resistant cysts attached to the substrate [63,64]. Another parasitic neobodonid is the recently described *Allobodo chlorophagus*, invading the utricles and the main filaments of the green siphonal alga *Codium fragile* and feeding on its chloroplasts and starch granules (plate A, 11) [65].

Parasitic parabodonids are represented by the genera *Trypanoplasma* and *Cryptobia*, which often used to be combined into one genus due to morphological similarity. *Trypanoplasma* spp. are extracellular parasites of fish bloodstream transmitted by haematophagous leeches during blood-feeding (plate A, 15) [66]. However, at least two species, *T. salmositica* and *T*. *bullocki,* can exit to the body surface where they reside in the mucus and can be transmitted to other fish by direct contact [57]. After ingestion with the blood, parasites multiply in the leech crop without significant changes in morphology and migrate to the proboscis sheath, wherefrom they are transmitted to the bloodstream of another fish [67]. The severity of infection—acute to chronic—apparently depends more on the level of mutual adaptation as well as the individual variation of host immunity between host and parasite than on parasitaemia [68–70]. There is only a single record of a trypanoplasma in a non-fish host, namely in a salamander [71].

The genus *Cryptobia* can be subdivided into three distinct ecological groups: (i) ectoparasites of fish, (ii) endoparasites of invertebrates and (iii) endoparasites of fish. Members of the first group, represented by *Cryptobia carassii*, live on fish gills and are regarded as commensals feeding on dead epithelium and microorganisms [57]. However, gill infections by *C. branchialis* are associated with high mortality in adult cultured carps, goldfish and catfish as well as in juvenile grass carp [72]. *Lamellasoma bacillaria*, described as a monoflagellated kinetoplastid living on the fish gills, may also belong to this group of cryptobiae [73].

In invertebrates, *Cryptobia* infections are quite diverse in terms of localization within the host and taxonomic groups parasitized. Some of them (*Cryptobia helicis, C. innominata* and *C. carinariae*) were found attached to the epithelium of spermatheca of floating sea snails and various pulmonates [74–76]. The vagina of haematophagous leeches often serves as a habitat of *C. vaginalis* (plate A, 16) [77], while *C. udonellae* was described from the excretory system of an ectoparasitic marine worm [78]. Other species were described from the intestine of a chaetognath (*C. sagittae*) and a freshwater planarian (*C. dendrocoeli*), the latter of which was also detected in the eggs, pointing to a potential transovarial transmission [79,80]. It is presumed that *Cryptobia* spp. from the reproductive system are transmitted via sexual contacts [75,81], while the ectoparasites of aquatic animals should have free-swimming swarmers, although this has not been confirmed [73]. The cryptobiae found in the intestinal contents of frogs and lizards appear to be accidentally ingested parasites of invertebrates, as judged by the morphology of the flagellates and uniqueness of such records [82,83].

In the third group of cryptobiae, encompassing piscine intestinal flagellates, six out of seven described species are known as specific parasites of marine fish. These species do not display any pathogenic effect and therefore are usually considered as commensals [57]. The only known freshwater representative of this group, *C. iubilans*, infects various cichlid fishes and causes gastroenteritis often associated with invasion of other organs, leading to high mortality [84–86]. Сryptobiae belonging to this group can be transmitted directly by ingestion from water and by feeding on infected corpses [57].

*Jarrelia atramenti*, a flagellate described from the blowhole mucus of a pygmy sperm whale, appears to be a harmless commensal feeding on detritus and/or bacteria [87]. Its resemblance to parasitic parabodonids, proposed to be evidence of their relatedness, may be in fact a parallelism caused by similar living conditions. Indeed, flexible body and flagellar attachment evolved independently in *Cryptobia*, *Trypanoplasma* and *Dimastigella*.

#### 2.1.3. Trypanosomatids

The family Trypanosomatidae contains exclusively obligate parasites and represents the most diverse kinetoplastid group in terms of the number of species described and/or revealed using molecular typing [88–90]. Among parasitic protists, it has the widest host range: animals (predominantly insects and vertebrates), flowering plants and even ciliates [91]. Based on the type of life cycle, trypanosomatids are usually subdivided into two non-taxonomic groups. Monoxenous species develop in a single host, whereas dixenous switch between two, of which one serves as a vector. Molecular phylogenies suggest that the most recent common ancestor of trypanosomatids was a monoxenous parasite of insects [92,93], with the dixenous lifestyle emerging independently at least three times in distantly related lineages of these flagellates [55].

##### 2.1.3.1. Monoxenous trypanosomatids

Most trypanosomatid genera are monoxenous and the overwhelming majority of their species parasitize two large groups of insects: Diptera and Heteroptera (i.e. flies and true bugs, respectively) [91,94]. Among other insects, used by them as hosts are Hymenoptera (bees, bumblebees, wasps and sawflies), Siphonaptera (fleas), Blattodea (cockroaches), Lepidoptera (moths) and Trichoptera (caddis flies). The single records of monoxenous trypanosomatids from a louse (Anoplura), a planthopper (Homoptera), a scorpion fly (Mecoptera) and a domestic cricket (Orthoptera) may refer to accidental non-specific infections [91]. The adaptation to insects, which are omnipresent, extremely diverse and abundant animals, probably predetermined the transition of these flagellates to other hosts. Trypanosomatids invaded Acari (ticks and mites) and freshwater ciliates living side-by-side with insects, vertebrates and plants. The two latter host groups are associated with dixenous trypanosomatids, although monoxenous species have also been occassionally reported from them [95,96]. The presence of trypanosomatids in nematodes and molluscs [97] may indicate a more complex evolutionary pathway of these flagellates, but first it requires confirmation with modern methods.

The ancestral and still most common lifestyle of monoxenous trypanosomatids includes stages that inhabit insect gut, usually being attached to its wall, and some either active (i.e. flagellate) or inactive (endomastigote or cyst-like amastigote) cells are discharged with faeces. Other insects become infected by feeding on contaminated substrates or directly on fresh faeces (coprophagy) [98]. In addition, the parasites can be transmitted between insects via cannibalism and predation, although the latter way is probably responsible only for the transmission of non-specific transient infections [99]. Some monoxenous trypanosomatids can migrate within insects to other locations in order to facilitate transmission [89]. Thus, parasitism in Malpighian tubules of female firebugs ensures timing the mass production of infective cyst-like amastigotes of *Blastocrithidia papi* to oviposition [100]. Haemocoel invasion allows the inheritance of *Herpetomonas swainei* between developmental phases of the host saw fly [101], while in the case of *Leptomonas pyrrhocoris*, this increases the efficiency of transmission by cannibalism [102]. The role of intracellular stages, which are very rare in life cycles of monoxenous trypanosomatids, is uncertain [103]. However, the potential to live intracellularly probably preconditioned transition of these flagellates to dixeny (see below) and parasitism in ciliates. The latter has been repeatedly described from various ciliate species where it was always associated with the macronucleus and, at least in some cases, effective transmission between host cells was observed pointing to specific relationships [104–107].

Although most monoxenous trypanosomatids are considered non-pathogenic or even commensals [98], this view is influenced by the fact that their effects on the hosts are poorly known and have been investigated in only a few practically important or model insect species. It was shown that trypanosomatid infections lead to elevated mortality rates in triatomine bugs, honeybees, sawflies, eye gnats, fruit flies, firebugs and water striders [101,108–112]. Other adverse consequences of trypanosomatid infections on insects include delayed development, decrease in body weight, disturbed digestion and excretion, lower endurance, impaired foraging efficiency and lower fecundity [113–118]. The above effects have a significant impact on host fitness, and thus trypanosomatids play an important role in controlling the population sizes of their hosts.

##### 2.1.3.2. Phytomonas

Some trypanosomatids acquired the ability to live in plants, on which their bug hosts feed and, thus, became dixenous. These flagellates belong to the genus *Phytomonas* and parasitize phloem, fruits, latex or seeds of various plants [119,120]. The bug hosts serve as vectors and, since the contaminative route of transmission to plants is not very effective, the parasites migrate from the intestine through haemocoel to salivary glands [96]. Here, the infective endomastigotes are formed, which are inoculated into plant juices with the bug's saliva during feeding [103,121,122]. Interestingly, in some species, no development occurs in the host gut, which is then used only for the transit of flagellates [103,123]. At least one phytomonad species, *P. nordicus*, became secondarily monoxenous, since it inhabits a predatory pentatomid bug [124]. The pathogenicity of *Phytomonas* for insects remains unknown, while their effect on plants ranges from asymptomatic infections to serious diseases of cultural plants [120]. *Phytomonas francai* living in lactiferous ducts of manioc is associated with root dystrophy; *P. leptovasorum* causes phloem necrosis and subsequent lethal wilt of coffee trees; *P. staheli* obstructing phloem of oil and coconut palms accounts for acute wilt in these plants; and an unnamed phytomonad is responsible for the withering of red ginger [96]. These diseases have a high impact on agriculture in developing countries and result in serious economic losses [119].

##### 2.1.3.3. *Leishmania* and related dixenous genera

The genera *Leishmania*, *Porcisia* and *Endotrypanum* represent a monophyletic group, whose parasitism in blood-sucking sandflies (Phlebotominae) allowed them to become dixenous parasites of mammals [125]. Secondarily, some *Leishmania* spp. changed either the vertebrate host or the vector: the subgenus *Sauroleishmania* switched from mammals to lizards and snakes, while the subgenus *Mundinia* started using biting midges (Ceratopogonidae) instead of sandflies [126–128]. *Leishmania* is most species-rich genus and many of its members are human parasites, which drew most attention to this group, while the information about *Porcisia* and *Endotrypanum* is scarce. The development of leishmaniae in vectors is confined to the intestine, although there are some differences between subgenera in the localization of the proliferative procyclic promastigotes (midgut, pylorus and/or hindgut) [127]. However, they eventually migrate to the anterior midgut, where they destroy the chitin lining of the stomodaeal valve and secrete a gel plug obstructing the alimentary canal, thus disturbing the normal sucking process [129]. An infected sandfly regurgitates the plug with metacyclic flagellates into the vertebrate bloodstream and due to the inability to swallow the blood makes more attempts increasing chances of spreading the parasites [130]. In the vertebrate, the metacyclic promastigotes are quickly taken up by phagocytic cells and proliferate in their phagolysosomes as amastigotes [131]. Depending on the behaviour of infected macrophages, leishmaniasis manifests itself as either cutaneous (skin ulcers), mucocutaneous (sores in the mucosa of nose, mouth or throat) or visceral, which affects internal organs such as the liver, spleen and bone marrow and is usually fatal without treatment [127].

About 20 species of *Leishmania*, belonging to the subgenera *Leishmania*, *Viannia* and *Mundinia* parasitize humans. They are responsible for up to one million new cases of leishmaniasis annually, of which up to 90 000 correspond to the visceral form [132]. The visceral form of the disease can be spread even outside the endemic areas either venereally or congenitally [133–136]. In addition to humans, *Leishmania* was reported to infect about 70 species of mammals (rodents, carnivores, xenartrans, hyraxes, marsupials, chyropterans, ungulates lagomorphs and primates), with most cases being asymptomatic. The only notable exceptions are canine visceral leishmaniasis, with severe symptoms in over 50% of cases [137], and rare cases of atypical cutaneous leishmaniasis in cows and horses [138,139]. *Porcisia* living in porcupines and *Endotrypanum* parasitizing sloths and squirrels do not appear to produce any symptoms [125]. However, *Leishmania colombiensis* (now assigned to *Endotrypanum*) is known to cause both cutaneous and visceral leishmaniasis-like diseases in humans [140,141].

##### 2.1.3.4. Trypanosoma

*Trypanosoma* is a very speciose genus enclosing approximately 500 species or over 60% of all described species of the family Trypanosomatidae (plates B and C, 18–40). While the frog-infecting type species (*T. sanguinis* = *T. rotatorium*) described by Gruby already in 1843 may be of marginal importance, ever since trypanosomes became the best-known protists. Life cycles of these flagellates vary considerably as they parasitize all classes of vertebrates (from agnathans to mammals) and are transmitted by a wide range of vectors including blood-sucking insects (flies, bugs, fleas and lice), ticks, leeches and even vampire bats [9,91]. In vertebrates, they occur most frequently as trypomastigotes, rarely as epimastigotes or amastigotes, while in invertebrates, they predominantly exhibit most trypomastigote or epimastigote morphology, or infrequently occur as promastigotes and amastigotes [92].

**Plate B.**
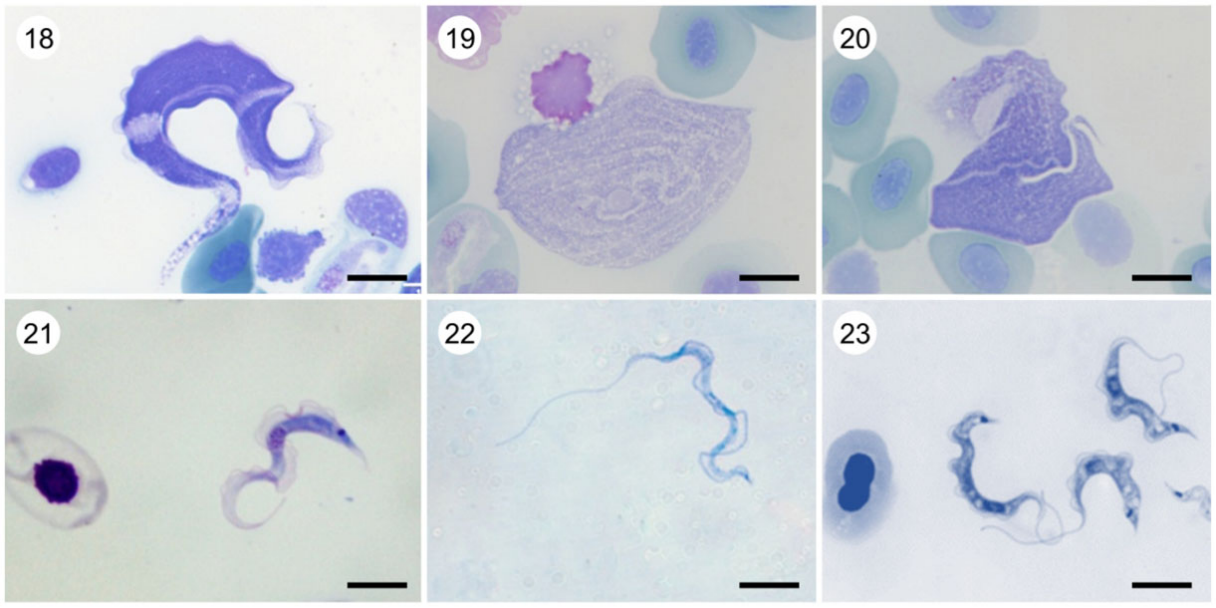
*Trypanosoma* (aquatic clade). Light micrographs of Giemsa-stained (18) *T*. (*Trypanosoma*) *rotatorium* ex *Pelophylax* kl. *esculentus* (provided by Klára Poloprudská); (19) *T*. (*Trypanosoma*) *loricatum* ex *Pelophylax* kl. *esculentus* (provided by Klára Poloprudská); (20) *T*. (*Trypanosoma*) *ranarum* ex *Pelophylax* kl. *esculentus* (provided by Klára Poloprudská); (21) *T*. (*Haematomonas*) *clandestinus* ex *Caiman yacare* (experimental infection) (provided by Erney Camargo and Marta Teixeira); (22) *T*. (*Haematomonas*) cf. *cobitis* ex *Cobitis* ‘*taenia*'; (23) *T*. (*Haematomonas*) sp. ex cichlid (provided by Iva Dyková). Scale bar, 10 µm (18–23).

**Plate C.**
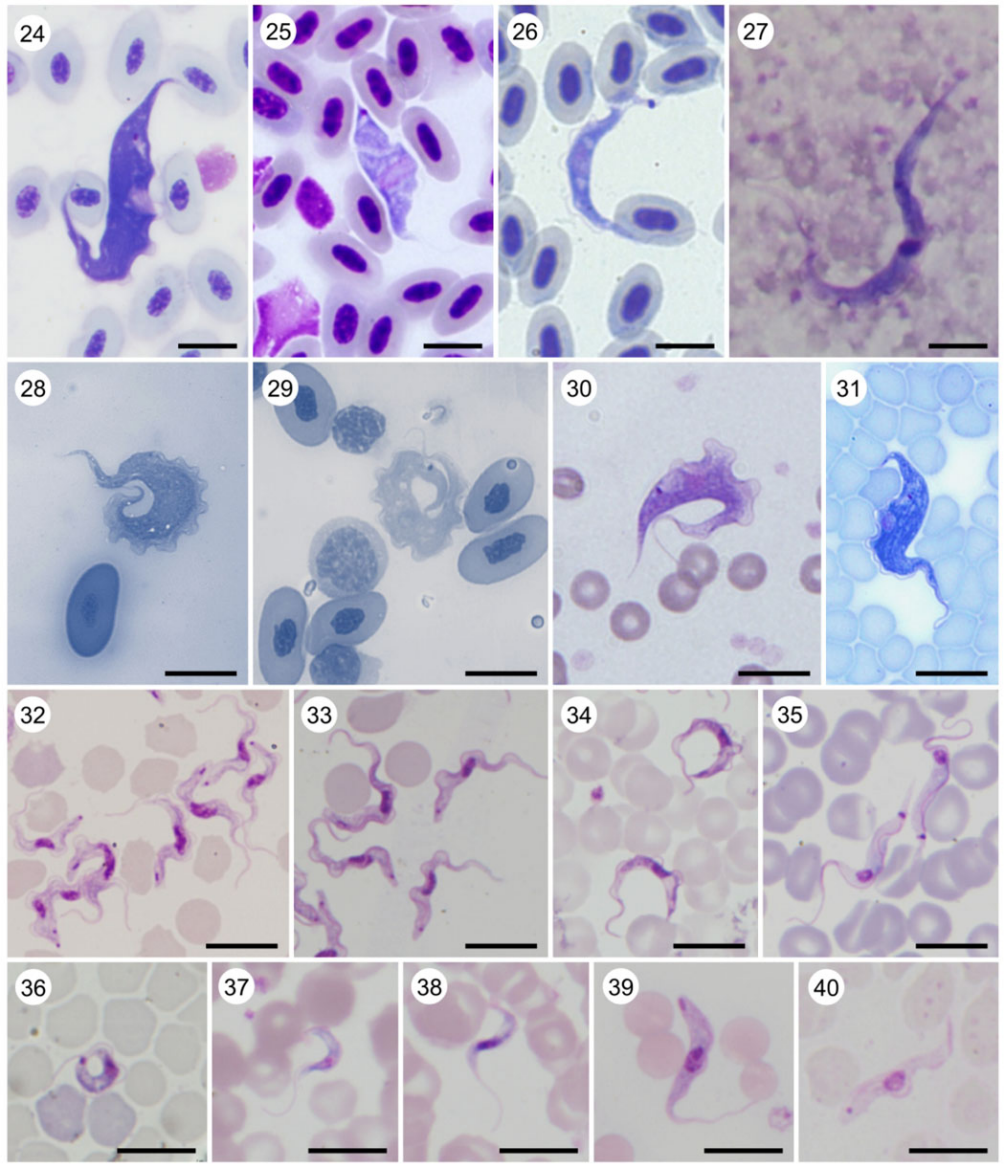
*Trypanosoma* (terrestrial clade). Light micrographs of Giemsa-stained (24) *T*. (*Trypanomorpha*) *avium* ex *Lanius collurio*; (25) *T*. (*Ornithotrypanum*) *everetti* (provided by Gediminas Valkiūnas); (26) *T*. (*Avitrypanum*) *culicavium* (experimental infection) (provided by Milena Svobodová); (27) *T*. (*Megatrypanum*) *theileri* ex cattle (provided by Andrei Mihalca); (28) *T*. (*Squamatrypanum*) *cascaveli* ex *Crotalus durissus* (provided by Erney Camargo and Marta Teixeira); (29) *T*. (*Crocotrypanum*) *terena* ex *Caiman yacare* (provided by Erney Camargo and Marta Teixeira); (30) *T*. (*Australotrypanum*) *copemani* ex *Bettongia penicillata* (provided by Sarah Keatley and Andrew Thompson) (31) *Trypanosoma livingstonei* ex African bat (provided by Erney Camargo and Marta Teixeira); (32) *T*. (*Trypanozoon*) *brucei brucei* ex mouse (experimental infection); (33) *T*. (*Trypanozoon*) *brucei equiperdum* ex mouse (experimental infection); (34) *T*. (*Trypanozoon*) *brucei evansi* ex mouse (experimental infection); (35) *T*. (*Herpetosoma*) *lewisi* ex *Rattus* sp.; (36) *T*. (*Aneza*) *vespertilionis* ex *Pipistrelus pipistrelus*; (37) *T*. (*Schizotrypanum*) *cruzi* (C-shape; experimental infection); (38) *T*. (*Schizotrypanum*) *cruzii* (S-shape; experimental infection); (39) *T*. (*Duttonella*) *vivax*; (40) *T*. (*Nannomonas*) *congolense*. Scale bar, 10 µm (24–40).

From the practical point of view, the most important mammalian trypanosomes were traditionally subdivided into two sections or intrageneric taxons that follow distinct developmental programmes [142]: Salivaria (derived from saliva), with the best-known member being the *Trypanosoma brucei* complex causing human African sleeping sickness and nagana in livestock and other animals (plate C, 32–34), terminate development in the salivary glands mouthparts of the vector and are transmitted to a vertebrate host by bite. Stercoraria (stercus = dung) exemplified by *Trypanosoma cruzi* complex species causing Chagas disease (plate C, 37–38), terminate development in the rear part of the digestive tract of the vector with the transmission to the vertebrate host being contaminative by excrements. With the advent of molecular phylogenetics, it became obvious that neither mammalian trypanosomes in general nor any of the two proposed sections represent monophyletic groups, and therefore they do not deserve a taxonomic status [89,143,144]. Nevertheless, the words salivarian/stercorarian still can refer to the type of development within the vector. Non-mammalian trypanosomes generally follow the stercorarian developmental programme, but in leeches, parasites migrate to the proboscis sheath to be transmitted during blood-sucking [57].

*Trypanosoma brucei evansi* and *T. b. equiperdum* are two notable exceptions (plate C, 33–34), as they lost the capacity to survive in the gut of an insect vector [145]*.* The former subspecies therefore switched to mechanical transmission, which allowed it to use non-specific vectors, while the latter adapted to the direct (venereal) transmission and thus became a monoxenous parasite [146]. In other species, direct transmission can also occur, but is facultative [147–150]. The most (in)famous species are *Trypanosoma brucei* and *T. cruzi*, which cause serious human diseases—sleeping sickness and Chagas disease, respectively [151]. The first one is transmitted by tsetse flies in Africa and invades various tissues, but primarily the blood and adipose tissue [152], as free-swimming trypomastigotes and eventually infects cerebrospinal fluid with fatal consequences [153]. Being a serious public health threat in the past, this disease is now on the way to elimination [154]. *Trypanosoma cruzi* is transmitted by triatomine bugs among a wide range of mammalian hosts, in which it develops in various organs and tissues as intracellular amastigotes [155]. In most cases, the disease does not manifest clinical signs at the beginning, but during the prolonged chronic phase, it significantly undermines health in the human population of South and Central America leading to increased mortality rates [156]. *Trypanosoma rangeli* has the same geographical distribution and vectors as *T. cruzi* and is also able to infect humans but appears to be non-pathogenic [157]. Some tsetse-transmitted African trypanosomes, such as *T. vivax*, *T. congolense* and *T. brucei brucei*, cause serious diseases in livestock, collectively named African animal trypanosomiasis. These diseases are associated with high mortality rates and lead to significant damage in animal husbandry, although some local breeds and wild animals acquired tolerance to them [158]. For the overwhelming majority of trypanosome species, their effects on the host are not known and they are often considered as non- or subpathogenic and can cause observable disease only under stress conditions. This is exemplified by piscine trypanosomes, which seem to be well tolerated in wild fish populations [159]. However, in farmed fish or wild juvenile individuals, infections are associated with high mortality rates due to anaemia, anorexia and tissue damage [160–163].

### 2.2. Taxonomy

This section contains nomenclatural changes and according to the ICZN requirements for publications in online-only journals, this work has been registered in Zoobank: urn:lsid:zoobank.org:pub:81EA01C5-8989-4BBD-9C64-04D81132307D.

Class Kinetoplastea Honigberg, 1963 emend. Vickerman, 1976 (tree B).

**Tree B.**
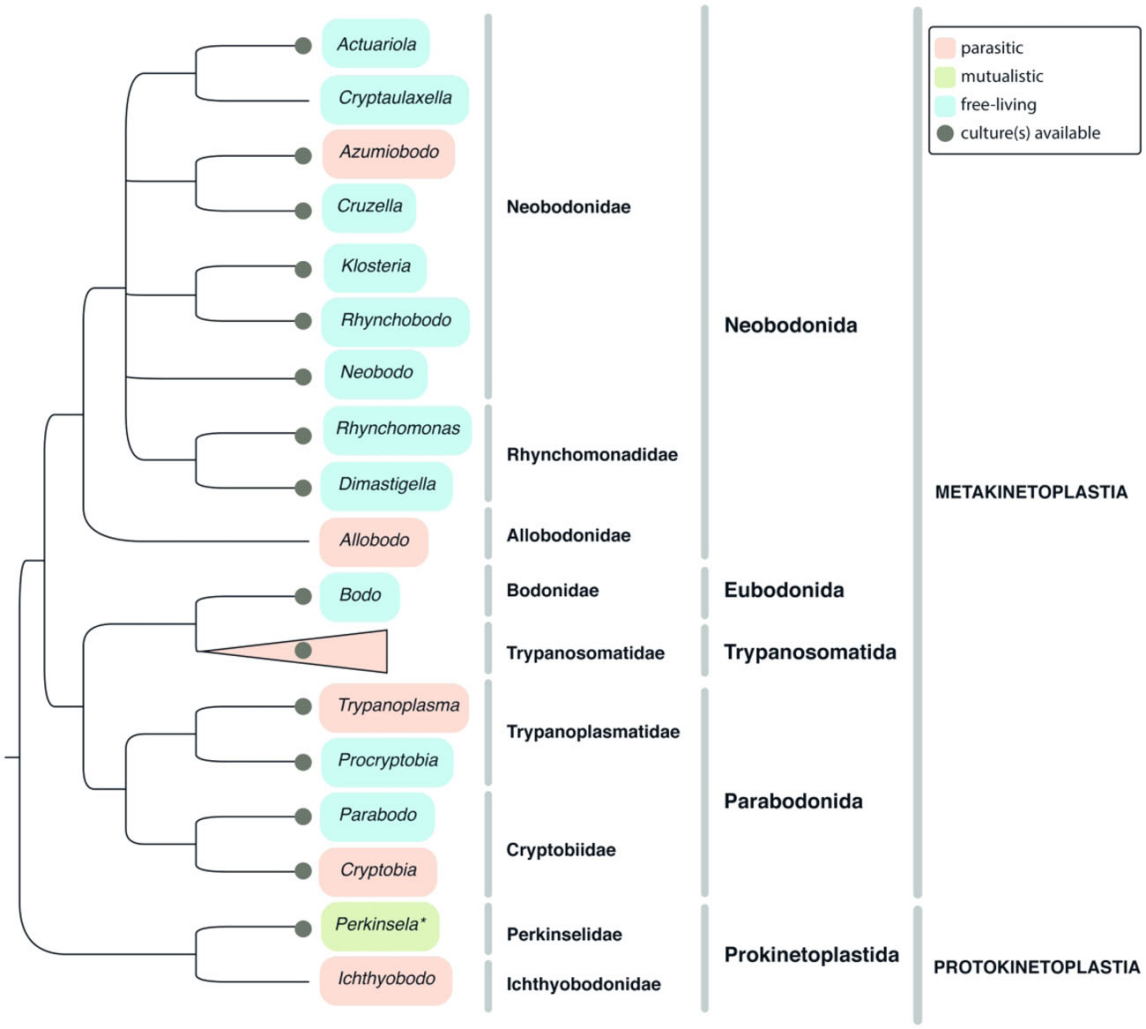
Kinetoplastea. A tree summarizing multiple phylogenetic reconstructions, mostly 18S rRNA gene-based. Highlighting denotes lifestyles (see graphical legend). Asterisk denotes that the protist can be cultivated only within its host. Host Trypanosomatidae clade is collapsed (shown with a triangle) and is presented in detail on a separate figure.

Possess kinetoplast, represented by one or several large masses of mitochondrial DNA termed kinetoplast DNA (kDNA). Four kinetoplast types are distinguished: eukinetoplast—dense network of interlocked DNA circles, prokinetoplast—single compact mass not organized into a network, polykinetoplast—several clusters scattered over the mitochondrial lumen, and pankinetoplast—a diffuse mass occupying a large portion of a mitochondrial lumen [164]. Ancestrally, kinetoplastids bear two heterodynamic flagella, of which one or both were lost in some lineages; mitochondrial RNA undergoes editing represented by deletions and insertions of uridine residues. Some features considered for a long time to be defining (e.g. polycistronic transcription of nuclear genes, *trans*-splicing via spliced leader RNA, compartmentalized glycolysis, base J, etc.) were recently shown to be present also in other euglenozoan lineages [6].

Note: Until relatively recently, all kinetoplastids have been classified into two large groups—bodonids (free-living, ectocommensals, ecto- or endoparasitic biflagellate species) and trypanosomatids (exclusively endoparasitic uniflagellate species). However, the 18S rRNA gene-based molecular phylogenetic analysis showed paraphyly of bodonids, which were subsequently separated into four orders [165]. Thus, now the term ‘bodonids’ for the designation of non-trypanosomatid kinetoplastids is deprecated. As judged by available environmental sequences, the diversity of kinetoplastids is much broader than that described to date, and there are some undiscovered lineages potentially representing new high-level taxa (up to the subclass) [166].

• Subclass Prokinetoplastia Vickerman, 2004. The phylogenetic group enclosing the genera *Ichthyobodo* and *Perkinsela* as judged by 18S rRNA gene-based trees [165].
  ○ Order Prokinetoplastida Vickerman, 2004. With the same definition as the subclass.
    ▪ Family Ichthyobodonidae Isaksen *et al*., 2007. Ectoparasitic on freshwater and marine fish; polykinetoplastic; biflagellate; the flagellar pocket extends to the lateral cell surface as a longitudinal groove; the modified anterior end (rostrum), present in trophozoites, is used for attachment [167]. Single genus.
      ▪ Genus *Ichthyobodo* Pinto, 1928. With the same definition as the family.
        Type species: *Costia necatrix* Henneguy, 1883 (= *Ichthyobodo necator*) (plate A, 17).
    ▪ Family Perkinselidae Kostygov, fam. nov.
      Diagnosis: The phylogenetic group comprising *Perkinsela* Dyková, Fiala and Peckova, 2008 (type genus) and related endosymbiotic forms as judged from 18S rRNA gene-based trees [168,169].
      ▪ Genus *Perkinsela* Dyková, Fiala and Pecková, 2008. Permanently endosymbiotic in the cytoplasm of various amoebae (*Paramoeba*, *Neoparamoeba*, *Janickina*, etc.), parasitophorous vacuole not formed; oval aflagellate cells; massive prokinetoplast; usually binucleate; microtubular corset reduced and present only in a thin layer on both sides of the kinetoplast; no oral apparatus, no flagellum [58,169].
        Type species: *Perkinsiella amoebae* Hollande, 1980 (= *Perkinsela amoebae*). Monotypic (plate A, 14).
        Note: Axenic cultivation is impossible, but can be grown in the host amoebae.
• Subclass Metakinetoplastia Vickerman, 2004. The phylogenetic group enclosing Neobodonida, Parabodonida, Eubodonida and Trypanosomatida as judged by 18S rRNA gene-based trees [165].
  ○ Order Eubodonida Vickerman 2004. Free-living; biflagellate, with non-tubular mastigonemes on the anterior flagellum; prokinetoplastic; phagotrophic, with anterolateral cytostome bordered by lappets and no conspicuous preoral ridge, cytopharynx traversing body [165]. Single family.
    ▪ Family Bodonidae Bütschli, 1883. With the same definition as the order. Single genus.
      ▪ Genus *Bodo* Ehrenberg, 1830. Solitary, free-living; phagotrophic; prokinetoplastic; free recurrent flagellum; non-prominent rostrum; lateral cytostome—cytopharynx complex without prismatic rod [165].
        Type species: *Bodo saltans* Ehrenberg, 1830 (plate A, 9).
  ○ Order Neobodonida Vickerman, 2004. Free-living, or parasitic; solitary; biflagellate, usually without mastigonemes, both flagella free or the posterior one attached to the cell body; pro- or polykinetoplastic; apical cytostome on preflagellar rostrum; phagotrophic [3,165].
    ▪ Family Allobodonidae Goodwin *et al*., 2018. The phylogenetic group enclosing *Allobodo* and related forms on 18S rRNA gene-based trees [65].
      ▪ Genus *Allobodo* Goodwin *et al*., 2018. Parasitic in seaweeds; both flagella free; with apical rostrum; phagotrophic, short tubular cytopharynx not supported by a microtubular rod; pankinetoplastic [65].
        Type species: *Allobodo chlorophagus* Goodwin, Lee, Kugrens and Simpson, 2018. Monotypic (plate A, 11).
    ▪ Family Neobodonidae Cavalier-Smith, 2016. Free-living or parasitic in animals; biflagellate, flagella free or attached; rostrum rigid; pro-, poly- or pankinetoplastic; phagotrophic, bacterivorous or eukaryovorous [170].
      Note: There is no evidence that the family is monophyletic.
      ▪ Genus *Actuariola* Stoeck, Schwarz, Boenigk, Schweikert, von der Heyden and Behnke, 2005. Free-living; solitary, phagotrophic; both flagella free and without mastigonemes; prokinetoplastic; cytopharynx supported by a non-prismatic microtubular rod [171].
        Type species: *Actuariola framvarensis* Stoeck, Schwarz, Boenigk, Schweikert, von der Heyden and Behnke, 2005. Monotypic (plate A, 1).
      ▪ Genus *Azumiobodo* Hirose, Nozawa, Kumagai and Kitamura, 2012. Parasitic in ascidians; anterior flagellum attached to the rostrum in basal part, posterior flagellum usually attached to the cell body; polykinetoplastic; cytostome at the apex of the long rostrum; curved cytopharynx, presence of supporting rod not assessed; unique globular bodies with electron-dense bands of various shapes [62].
        Type species: *Azumiobodo hoyamushi* Hirose, Nozawa, Kumagai and Kitamura, 2012. Monotypic (plate A, 5).
      ▪ Genus *Cruzella* Faria, Cunha and Pinto, 1922, emend Kostygov.
        Diagnosis: Free-living, solitary, two mastigoneme-free flagella originating under beak-shaped rostrum; phagotrophic, cytostome on rostrum tip, well-developed tubular cytopharynx without supporting microtubular rod; polykinetoplastic; intensive metaboly [172–174].
        Type species: *Cruzella marina* Faria, Cunha and Pinto, 1922. Monotypic (plate A, 10).
      ▪ Genus *Cryptaulaxella* Kostygov, nom. nov.
        Diagnosis: Free-living, solitary; both flagella free; prominent spiral groove on the surface; the presence of extrusomes questionable; ultrastructure not studied; type of kinetoplast uncertain [49,175].
        Type species: *Spiromonas akopos* Skuja, 1939 (= *Cryptaulaxella akopos* comb. nov.).
        Justification: This newly proposed name refers to the genus previously known as: (i) *Spiromonas* Skuja, 1939—homonym of *Spiromonas* Perty, 1852 [176] (Dinoflagellata); (ii) *Cryptaulax* Skuja, 1948—homonym of *Cryptaulax* Tate, 1869 [177] (Gastropoda) and *Cryptaulax* Cameron, 1906 [178] (Insecta); and (iii) *Cryptaulaxoides* Novarino, 1996—homonym of *Cryptaulaxoides* Uchida 1940 (Insecta).
        Etymology: The new name and the two previous ones share the Greek roots *κρυ*π*τ*ός (hidden) and *α*ὖ*λαξ* (furrow), referring to the distinctive feature of the genus, and the feminine gender.
        Note: All described species were once assigned to *Rhynchobodo* and the diplonemid *Hemistasia* based on light microscopy [179]. Molecular phylogenetic inference showed that flagellates identified as *Rhynchobodo* and *Cryptaulax*/*Cryptaulaxoides* are unrelated [180].
      ▪ Genus *Klosteria* Mylnikov and Nikolaev, 2003. Free-living, solitary; both flagella free, arise from a subapical flagellar pocket and bear short acronemes, anterior one with mastigonemes; rostrum not prominent; phagotrophic, cytopharynx tubular, without supporting microtubular rod; cytostome lips absent; pankinetoplastic; trichocysts near ventral side of the flagellar pocket [181].
        Type species: *Klosteria bodomorphis* Mylnikov and Nikolaev, 2003. Monotypic (plate A, 8).
      ▪ Genus *Neobodo* Vickerman, 2004. Free-living; solitary, phagotrophic; biflagellate with free posterior flagellum; prokinetoplastic; cytopharynx supported by a prismatic microtubular rod [165].
        Type species: *Bodo designis* Skuja, 1948 (= *Neobodo designis*) (plate A, 2).
        Note: as judged by available phylogenies, the genus is polyphyletic [65,180].
      ▪ Genus *Rhynchobodo* Vørs, 1992. Free-living, solitary; flagella exit subapically and bear acronemes; phagotrophic; well-developed rostrum with apical cytostome, tubular cytopharynx and multiple extrusomes; conspicuous spiral groove on the body surface; polykinetoplastic [182] (plate A, 4).
        Type species: *Cryptaulax taeniata* Skuja, 1956 (= *Rhynchobodo taeniata*).
        Note: Authorship of this name is often attributed to Lackey who mistakenly used it instead of *Rhynchomonas* [183]. However, only Vørs made the name available by providing the genus description and specifying the type species [182].
    ▪ Family Rhynchomonadinae Cavalier-Smith, 2016. Solitary, free-living; biflagellate, the anterior flagellum adheres to the flexible proboscis and they move together, the posterior flagellum is used for gliding and attached to the body at least in its proximal part; cytopharynx not supported by a microtubular rod [170].
      ▪ Genus *Dimastigella* Sandon, 1928. Free-living, in soil or freshwater; anterior flagellum significantly longer than the proboscis, posterior flagellum attached to the cell body across the whole length of the latter; cytostome on or under rostrum; phagotrophic; polykinetoplastic [184,185] (plate A, 6).
        Type species: *Dimastigella trypaniformis* Sandon, 1928.
      ▪ Genus *Rhynchomonas* Klebs, 1892. Free-living, short anterior flagellum is attached to a long rostrum representing a motile proboscis, posterior flagellum attached to the cell body in the proximal part, both flagella with mastigonemes; phagotrophic; prokinetoplastic [186].
        Type species: *Heteromita nasuta* Stokes, 1888 (= *Rhynchomonas nasuta*) (plate A, 3).
  ○ Order Parabodonida Vickerman, 2004. Clade enclosing the genera *Cryptobia*, *Parabodo*, *Procryptobia* and *Trypanoplasma*. Free-living, commensal or parasitic; biflagellate, without mastigonemes, posterior flagellum attached or free; pro-, poly- or pankinetoplastic; phagotrophic or osmotrophic; anterolateral cytostome with or without developed cytopharynx [3,165]. Previously, parasitic representatives of this group were considered a single lineage and often lumped into one genus (*Cryptobia*), but molecular phylogenetic analyses showed their polyphyly [187].
    ▪ Family Cryptobiidae Poche, 1911 emend. Kostygov. Clade uniting the genera *Cryptobia* and *Parabodo* based on 18S rRNA gene phylogenies [68,188].
      ▪ Genus *Cryptobia* Leidy, 1846. Parasites/commensals of fish (on gills or in the gut) or various invertebrates (in the lumen of reproductive, digestive or excretory organs) [73,78,189]; recurrent flagellum attached to cell body without the formation of undulating membrane, its posterior part is used for attachment to host epithelium; conspicuous ventral furrow; phagotrophic with well-developed but miniaturized cytopharynx in most species; subpellicular microtubules extend to whole-cell length; pro- or pankinetoplastic [81,164] (plate A, 16).
        Type species: *Cryptobia helicis* Leidy, 1846.
        Note: The genus may be paraphyletic with respect to *Parabodo* as judged by sequence data on two species from invertebrates and fish, although the relationships are poorly resolved [190].
      ▪ Genus *Parabodo* Skuja, 1939 emend. Vickerman, 2004. Free-living, solitary; posterior flagellum free; the cytostome is placed at the anterior end of the cell making the latter bifurcate, well-developed cytopharynx; subpellicular microtubules extend to whole-cell length; prokinetoplastic [191,192] (plate A, 13).
        Type species: *Parabodo nitrophilus* Skuja, 1939.
    ▪ Family Trypanoplasmatidae Hartmann and Chagas, 1910 emend. Kostygov. The clade uniting the genera *Procryptobia* and *Trypanoplasma* based on 18S rRNA gene phylogenies [68,188].
      ▪ Genus *Procryptobia* Vickerman, 1978. Solitary, free-living, prokinetoplastic; recurrent flagellum attached to the cell surface, ventral groove absent; short anterolateral rostrum; cell bears subpellicular microtubules only in the anterior portion and easily changes shape; phagotrophic [52,193] (plate A, 12).
        Type species: *Procryptobia vorax* Vickerman, 1978.
      ▪ Genus *Trypanoplasma* Laveran and Mesnil, 1901. Leech-transmitted obligate hemoparasites of fish; posterior flagellum attached to the cell body forming a conspicuous undulating membrane bordering a ventral furrow; osmotrophic, cytopharynx reduced; subpellicular microtubules extend to whole-cell length; megakinetoplast [66,164].
        Type species: *Trypanoplasma borreli* Laveran and Mesnil, 1901 (plate A, 15).
  ○ Order Trypanosomatida Kent, 1880. Monoxenous or dixenous obligatory endoparasites of arthropods, leeches, vertebrates, plants and ciliates; single flagellum, emerging from flagellar pocket apically or laterally, is mastigoneme-free and oriented anteriorly; eukinetoplastic with the kDNA network attached to the basal body of the flagellum [194]; phagotrophic or osmotrophic; cytostome–cytopharyngeal complex fully developed only in a few representatives, while the majority has no cytopharynx, and cytostome is present as a shallow pit or completely absent [195,196]. For a long time, the classification was based on the presence of the following morphotypes in the cell cycle: promastigote (elongated with apical flagellum and prenuclear kinetoplast), choanomastigote (shortened, with apical flagellum and prenuclear kinetoplast), opisthomastigote (elongated, with apical flagellum and postnuclear kinetoplast), opisthomorph (shortened, with apical flagellum and postnuclear kinetoplast), epimastigote (with lateral flagellum attached to the cell body and prenuclear kinetoplast), trypomastigote (with lateral flagellum attached to the cell body and postnuclear kinetoplast), amastigote/endomastigote (flagellum not emerging from the pocket, the first variant predominantly used when flagellum is very short) and cyst-like amastigote (compact cells with dense cytoplasm, completely lacking flagellum). Single family. The taxonomic changes introduced here follow the guidelines specified for this group previously [197].
    ▪ Family Trypanosomatidae Doflein, 1901. With the same definition as the order (tree C).
      Note: Historically, all monoxenous genera were often termed ‘lower trypanosomatids', but after the switch to the phylogeny-oriented paradigm, this concept has been abandoned [92]. As an alternative, a colocation ‘insect trypanosomatids’, which has no evolutionary connotations, is often used.
      ▪ Subfamily Trypanosomatinae Doflein, 1901. A distinct clade on phylogenetic trees is based on 18S rRNA, gGAPDH and multiple protein-coding genes enclosing the genus *Trypanosoma* [10]. Single genus.

**Tree C.**
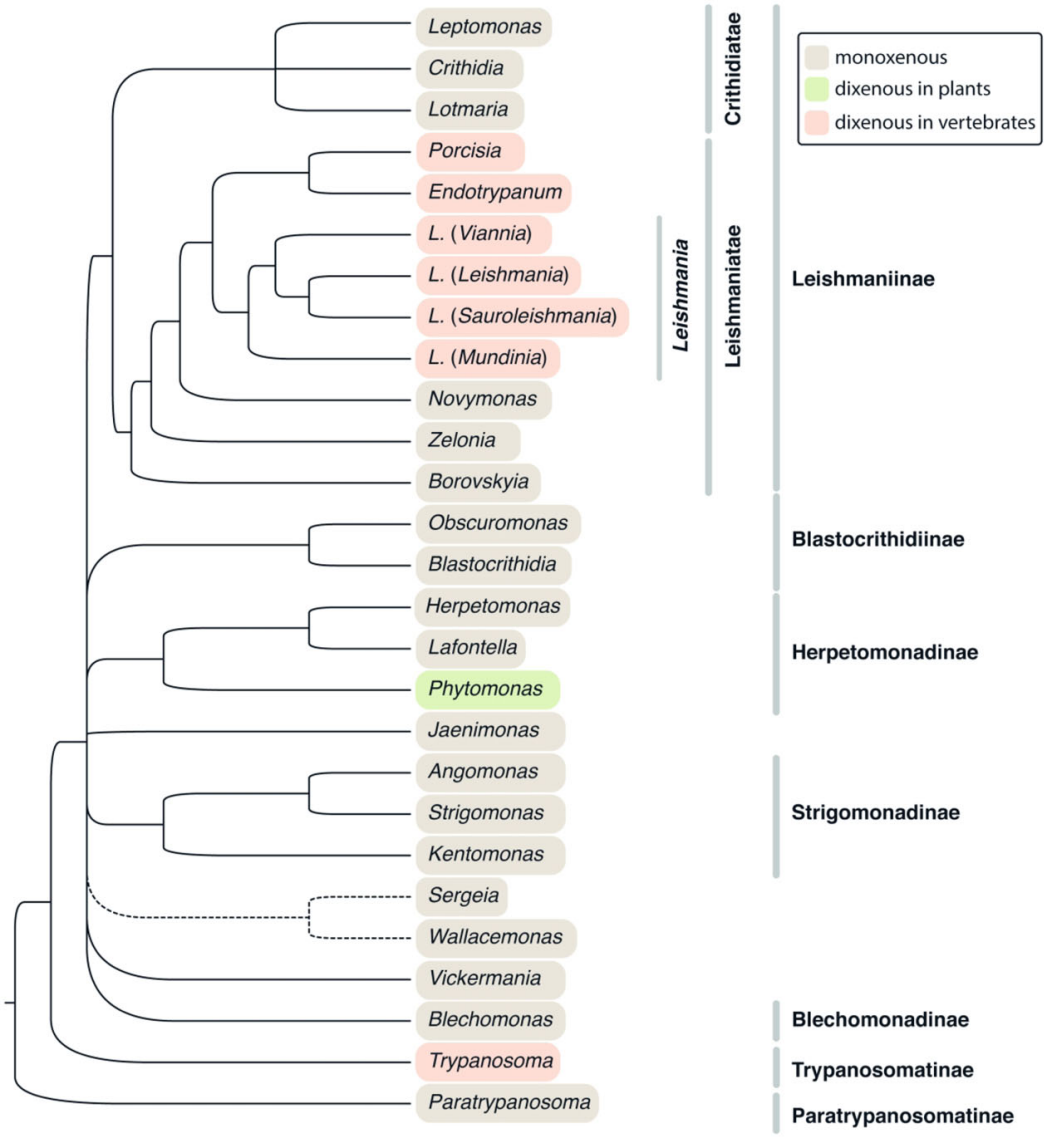
Trypanosomatidae. A tree summarizing multiple phylogenetic reconstructions, mostly 18S rRNA gene-based. Dashed line denotes an unstable clade, which is disrupted when certain taxa are included into the analysis. For the genus *Leishmania*, relationships between its four subgenera are also shown. Highlighting denotes types of the life cycles (see graphical legend).

Genus *Trypanosoma* Gruby, 1843. Dixenous parasites of all classes of vertebrates (in blood and tissues) transmitted by blood-sucking arthropods or leeches (digestive tract and salivary glands); trypomastigotes and amastigotes (in vertebrates) or epimastigotes and trypomastigotes (in invertebrates) (plates B and C, 18–40) (tree D).

**Tree D.**
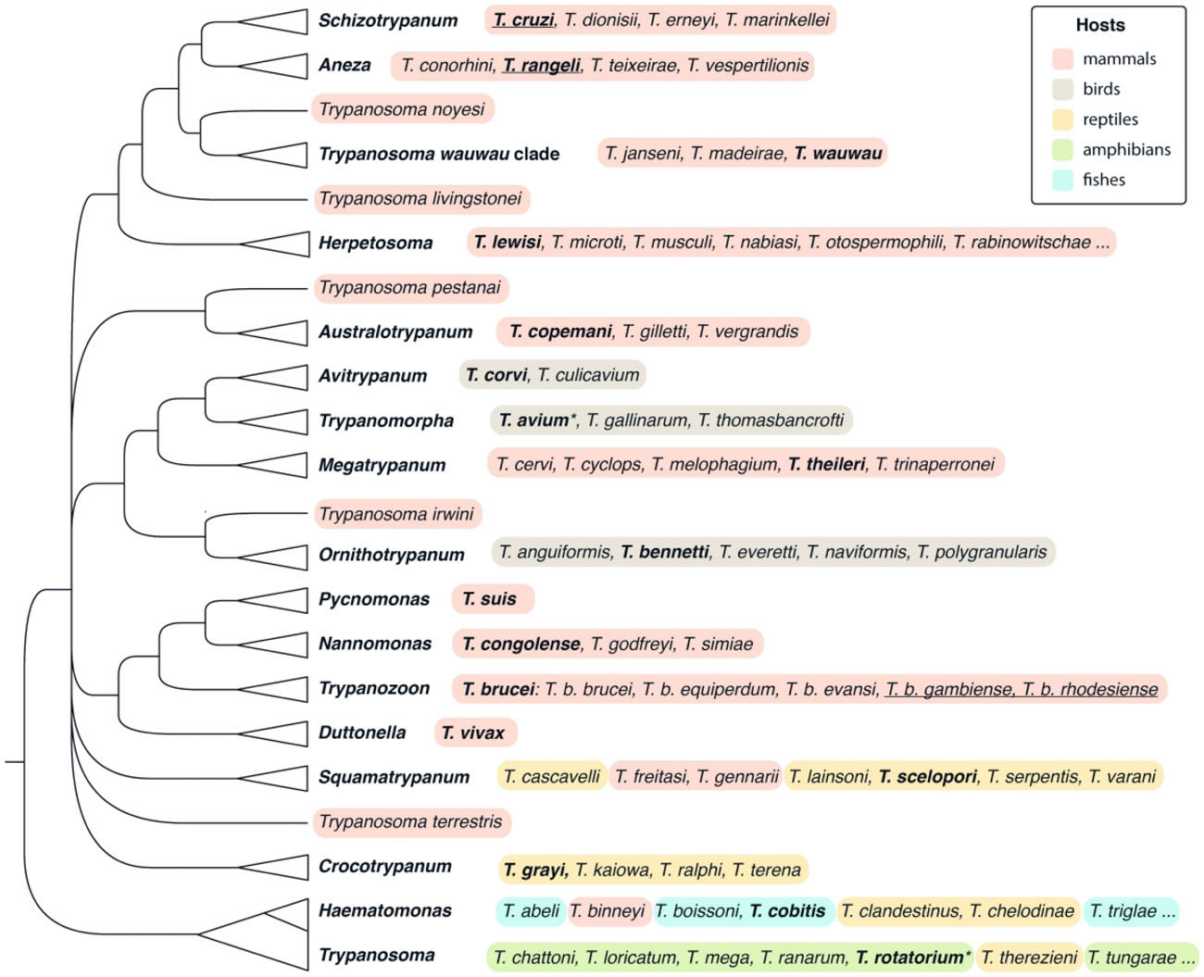
*Trypanosoma*. A tree summarizing multiple phylogenetic reconstructions, mostly 18S rRNA gene-based. All species or a selection of the most important ones (marked with three dots at the end), for which affiliation with a given subgenus and/or clade was confirmed using molecular phylogenetic methods, are listed. Highlighting denotes orders of vertebrate hosts (see graphical legend). Human parasites are underlined. Type species are shown in bold and the names accepted as senior subjective synonyms are marked with an asterisk.

Type species: *Trypanosoma sanguinis* Gruby, 1843 (junior subjective synonym of *T. rotatorium* (Mayer, 1843)).

Note: Historically, mammalian trypanosomes were subdivided into the sections Stercoraria (*Herpetosoma*, *Megatrypanum* and *Schizotrypanum*) and Salivaria (*Duttonella*, *Nannomonas*, *Pycnomonas* and *Trypanozoon*). Being long-established and of practical importance for the community of medical and veterinary doctors, this mammalian-centred classification is incorrect from the phylogenetic point of view (see Biology of Kinetoplastea), as it not only does not correspond with the known diversity of trypanosomes but also cannot accommodate species transmitted by other modes. Moreover, mammalian trypanosomes are not monophyletic and the names stercoraria and salivaria, which were proposed to reflect the differences between the modes of transmission, are non-taxonomical and have only historical value.

In parallel, the genus *Trypanosoma* was divided into several subgenera based on rather subtle morphological differences. Interestingly, this historical taxonomical classification in general corresponds well with the current phylogenetic analysis. However, despite the indisputable usefulness of this classification, in recent decades, the usage of subgenera was largely omitted (probably because of anticipated—but not materialized—conflicts between the morphology- and phylogeny-based systems). As a consequence, newly emerging clades in the expanding phylogenetic trees were named after the best-known representatives, causing confusion. Here, we revive the original subgeneric concept, and by erecting several new subgenera achieve mutual harmonization of the old morphological and the modern phylogenetic approaches. We believe that this taxonomical system reflects best the true diversity of these important parasites.

Species for which only morphological but no sequence information is available are marked with an asterisk (*). We have mapped the hosts (classes of vertebrates) onto the phylogenetic trees, highlighting associations among clades and their vertebrate host.

• ‘the aquatic clade’ (monophyletic) (plate B, 18–23).
  Note: Molecular phylogenies confirmed the monophyly of the genus *Trypanosoma* [198] and its subdivision into the aquatic and terrestrial clades [199–201]. In most reconstructions, the aquatic clade was further split into the ‘fish/turtle’ and ‘amphibian’ subgroups. While the former was invariably monophyletic, depending on the taxonomic set used, the latter appeared either as monophyletic or unresolved until an analysis including some key frog species demonstrated its clear paraphyly [202–204]. The ‘fish/turtle’ subgroup, which also includes trypanosomes from a platypus (*T. binneyi* Mackerras, 1959) and a crocodile (*T. clandestinus* Teixeira and Camargo, 2015) is here designated as the subgenus *Haematomonas*. The remaining aquatic trypanosomes, mostly parasitizing frogs (with *T. therezieni* Brygoo, 1963 infecting chameleons being a single known exception), fall into the paraphyletic subgenus *Trypanosoma*.
▪ Subgenus *Haematomonas* Mitrophanow, 1883 emend. Votýpka and Kostygov. Diagnosis: Leech-transmitted parasites of aquatic vertebrates. Morphologically variable medium to large conspicuously elongated trypomastigotes with a notably bent body, undulating membrane including free flagellum, and with the kinetoplast situated close to the posterior end of the body. Defined by 18S rRNA-based phylogenetic analyses.
  Type species: *Haematomonas cobitis* Mitrophanow, 1883 (= *Trypanosoma cobitis*).
  Note: Mitrophanow described two monoflagellates from the European freshwater fish and placed them into the new genus *Haematomonas* as *H. cobitis* (from a weatherfish, *Misgurnus fossilis*; formerly genus *Cobitis*) and *H. carassii* (from a crucian carp, *Carassius carassius*) which were both later reclassified into the genus *Trypanosoma* [205]. Since then, more than 190 trypanosome species have been described from both marine and freshwater jawless, cartilaginous and bony fish worldwide [206]. Phylogenetic analyses [202,204,207,208] revealed three monophyletic clades within this subgenus: (i) freshwater fish trypanosomes (including *T. clandestinus* Teixeira and Camargo, 2016 from crocodiles); (ii) marine fish trypanosomes (including *T. rajae* Laveran and Mesnil, 1902 from rays); (iii) turtle trypanosomes (including *T. binneyi* from a platypus). The following species are included in published phylogenetic trees: *T. abeli*, *T. binneyi*, *T. boissoni*, *T. cobitis*, *T. chelodinae*, *T. clandestinus*, *T. epinepheli*, *T. fulvidraco*, *T. granulosum*, *T. mocambicum*, *T. murmanensis*, *T. nudigobii*, *T. ophiocephali*, *T. pleuronectidium*, *T. pseudobagri*, *T. rajae*, *T. sinipercae* and *T. triglae*.
▪ Subgenus *Trypanosoma* Gruby, 1843 emend. Votýpka and Kostygov.
  Diagnosis: Morphologically variable medium to large trypomastigotes characterized by a wide range of forms with remarkable morphological plasticity; besides classical fusiform trypomastigotes, there are rounded, oval, claviform, fan-shaped, leaf-like, or irregular cells with or without a free flagellum, and longitudinal or spiral striations. Defined by 18S rRNA-based phylogenetic analyses.
  Type species: *Trypanosoma sanguinis* Gruby, 1843 (junior subjective synonym of *T. rotatorium* [209]).
  Note: Mayer in 1843 found in the blood of a frog (*Rana esculenta*) captured in Germany two organisms that he named *Amoeba rotatoria* and *Paramaecium loricatum*, which were later recognized as first-ever described trypanosomes [209]. Gruby published later the same year a description of a haemoflagellate from the blood of a frog in France and named it *Trypanosoma sanguinis* (from Greek *trypanon*, an auger; *soma*, body) [210]. In 1901, Doflein created the family Trypanosomidae, in which the genus *Trypanosoma* was subdivided into three subgenera including the nominotypical one—*Trypanosoma* with *T. sanguinis* Gruby 1843 as a type [205]. In 1926, International Commission on Zoological Nomenclature accepted *T. rotatorium* Mayer 1843 as the senior synonym of *T. sanguinis* Gruby 1843 [211].
  The following described species are included in published phylogenetic trees: *T. chattoni*, *T. fallisi*, *T. herthameyeri*, *T. loricatum*, *T. mega*, *T. neveulemairei*, *T. percae*, *T. ranarum*, *T. rotatorium*, *T. therezieni* and *T. tungarae*.
• ‘the terrestrial clade’ (monophyletic) (plate C, 24–40).
  Note: The internal classification of trypanosomes from terrestrial hosts is rather confusing. Mammalian trypanosomes, subdivided into the sections stercoraria and salivaria, were mostly singled out, followed by the avian trypanosome branch(es). Additionally, new clades appeared gradually in phylogenetic studies, the inclusion of which is a significant problem. Moreover, these above-mentioned groups are not monophyletic, making the internal system of the terrestrial clade unstable. We have attempted to rectify the situation by building a system that accommodates taxonomic units, for which sequence information is available.
  ○ avian subgenera (paraphyletic)
    Based on comparative morphology and developmental cycles, avian trypanosomes were hypothesized to be closely related to the subgenus *Megatrypanum* that infects ruminants [212,213]. This assumption is now supported by phylogenetic and phylogenomic studies that have also shown paraphyly of avian trypanosomes, represented by at least three distinct lineages (*T. avium*, *T. corvi* and *T. bennetti* clades) [144,214,215]. These groups are distinguishable by the morphology of bloodstream stages and the kinetoplast thickness [216,217].
    ▪ Subgenus *Trypanomorpha* Woodcock, 1906 emend. Votýpka
      Diagnosis: Medium to large size trypomastigotes (40–100 μm) with longitudinal striations (myonemes), central oval nucleus, prominent undulating membrane and small kinetoplast to which the free flagellum is anterior. Cells in culture have kinetoplast exceptionally thick (greater than 500 nm). Defined by 18S rRNA-based phylogenetic analyses; cosmopolitan distribution.
      Type species: *Trypanosoma noctuae* Schaudinn, 1904 (junior subjective synonym of *T. avium* Danilewsky, 1885, see note).
      Note: For the type species described from a European little owl *Athene noctua* [218], neither culture nor sequence data are available. Both exist for *T. thomasbancrofti* Šlapeta, 2016 and also for less clearly defined *T. avium* Danilewsky, 1885 and *T. gallinarum* Bruce *et al*., 1911 [214–217,219]. The vaguely defined species *Trypanosoma avium* was described in 1885 by Danilewsky from birds, but the type material was not preserved [220]. In 1903, Laveran proposed to restrict this species name to parasites of owls [221]; however, the name was often used to designate any bird trypanosome.
    ▪ Subgenus *Avitrypanum* Votýpka, subgen. nov.
      Diagnosis: Medium to large trypomastigotes (about 40–80 μm) in the bloodstream of bird hosts with longitudinal striations (myonemes), nucleus positioned centrally and posterior kinetoplast; the thickness of kinetoplast in cultured cells is less than 500 nm. Defined by 18S rRNA-based phylogenies; cosmopolitan distribution.
      Type species: *Trypanosoma corvi* Stephens and Christophers, 1908, here designated.
      Etymology: The generic name refers to the fact that trypanosomes come from bird hosts, the order Aves (the Latin name for a bird is *avis*).
      Note: Baker [222] emended *T. corvi* and restricted the use of the name to large trypanosomes from non-American corvids and also from other bird families. The species was re-described [223] and phylogenetically characterized [214] and forms the *T. corvi* clade along with *T. culicavium* Votýpka *et al*., 2012. Although morphologically indistinguishable from the subgenus *Trypanomorpha* (*T. avium* clade), *Aviotrypanum* (*T. corvi* clade) is not directly related to it [144,216,217], justifying separate treatment.
    ▪ Subgenus *Ornithotrypanum* Votýpka, subgen. nov.
      Diagnosis: Small to medium-size non-striated avian trypanosomes (less than 40 µm in length) with kinetoplast situated close to the posterior end of the body. Kinetoplast thickness of cells in culture below 500 nm. Defined by 18S rRNA-based phylogenetic analyses; cosmopolitan distribution.
      Type species: *Trypanosoma bennetti* Kirkpatrick, Terway-Thompson and Iyengar, 1986, here designated.
      Etymology: The generic name refers to the fact that trypanosomes come from bird hosts, the order Aves (the ancient Greek name for a bird is *órnῑs*; ὄ*ρν*ῑς).
      Note: Out of a number of morphologically described species parasitizing wild birds, for five of them (*T. anguiformis* Valkiūnas *et al*., 2011*, T. bennetti*, *T. everetti* Molyneux, 1973, *T. naviformis* Sehgal *et al*., 2015 and *T. polygranularis* Valkiunas *et al*., 2011) sequence data are available [144,224,225]. Their divergence justifies the establishment of a new subgenus—*Ornithotrypanum*.
      Phylogenomic approach revealed that the *T. bennetti* (= *Ornithotrypanum*) and *T. avium* (= *Trypanomorpha*) clades are nested within the mammalian clade and are paraphyletic with respect to the ruminants-infecting *Trypanosoma theileri* Laveran, 1902 [144], thus breaking the monophyly of mammalian trypanosomes. Interestingly, the host generalism of avian trypanosomes contrasts with the host specificity observed for some mammalian flagellates.
  ○ ‘mammalian subgenera (stercoraria)’ (polyphyletic)
    ▪ Subgenus *Herpetosoma* Doflein, 1901.
      Diagnosis: Medium-size trypomastigotes (20–40 µm) with long pointed posterior extremity, large rod-like subterminal kinetoplast but well away from the posterior end and long free flagellum. Parasitizing a wide range of rodents and lagomorphs, as amastigote and/or epimastigotes.
      Type species: *Herpetosoma lewisi* Kent, 1880 (= *Trypanosoma lewisi*).
      Note: This globally distributed species found in more than 100 rodent species (predominantly *Rattus* spp.) and rarely also in humans [226], is transmitted by the ingestion of fleas *Xenopsylla cheopis* and *Nosopsyllus fasciatus* or in their faeces. Considered largely non-pathogenic for rodents, it can cause serious disease in unnatural hosts. After its introduction in synanthropic rats to Christmas Island, it drove the endemic rat *Rattus macleari* to extinction, being the only known cases of a trypanosomatid responsible for the extinction of its host species.
      Molecular phylogenies revealed the polyphyly of *Herpetosoma* [143,227–229], excluding *T. rangeli* Tejera, 1920 into a newly established subgenus *Tejeraia* (now *Aneza*), keeping *Herpetosoma* (also named *T. lewisi*-like clade) monophyletic. 18S rRNA sequences are available for the following species: *T. niviventerae* (rat, *Niviventer confusianus*), *T. musculi* (mouse, *Mus musculus*/*domesticus*), *T. grosi* (field mouse, *Apodemus* spp.), *T. microti* (vole, *Microtus* spp.), *T. evotomys* (bank vole, *Clethrionomys glareolus*), *T. rabinowitschae* (hamster, *Cricetus cricetus*), *T. blanchardi* (dormouse, *Eliomys quercinus*), *T. kuseli* (squirrel, *Pteromys volans*), *T. ostospermophili* (squirrel, *Urocitellus* spp.) and *T. nabiasi* (rabbit, *Oryctolagus cuniculis*), while molecular data are not available for approximately 30 more species, including *T. acomys, *T. acouchii, *T. ellobii and *T. lemmi.
      The questions whether three phylogenetically related species with *Megatrypanum*-like morphology and basal phylogenetic position, *T. talpae* (European mole, *Talpa europaea*), *T. sapaensis* (white-toothed shrew, *Crocidura dracula*) and *T. anourosoricus* (mole shrew, *Anourosorex yamashinai*) should be included into the subgenus *Herpetosoma* remains unresolved [230].
    ▪ Subgenus *Megatrypanum* Hoare, 1964
      Diagnosis: Large trypomastigotes (40–100 µm) with long pointed posterior extremity; medium nonterminal kinetoplast located near the nucleus and far from the posterior end of the body; long free flagellum; reproduction as epimastigotes in the mammalian host.
      Type species: *Trypanosoma theileri* Laveran, 1902; a cosmopolitan non-pathogenic bovine trypanosome transmitted by tabanids.
      Note: Other members are ovine *T. melophagium* Flu, 1908 using a sheep ked (*Melophagus ovinus*) as a vector, while caprine *T. theodori Hoare, 1931 is transmitted by a goat ked (*Lipoptena capreoli*). Two species of cervid trypanosomes, European and North American *T. cervi* Kingston and Morton, 1975 and newly described Pan-American *T. trinaperronei* Teixeira, Camargo and García, 2020, are transmitted by deer keds (*Lipoptena cervi* and *L. mazamae*) [231]. Tabanids probably transmit *T. stefanskii Kingston *et al*., 1992 from a roe deer (*Capreolus capreolus*) [232] and *T. ingens Bruce *et al*., 1909 from African antelopes [233]. Vectors remain unknown for the closely related simian *T. cyclops* Weinman, 1972 and *T. lucknowi Weinman, White and Antipa, 1984 from *Macaca* monkeys. Morphological [234,235] and phylogenetic studies [199,236] justify inclusion of these two species into the subgenus *Megatrypanum*.
    Note: Probably least settled within the former stercoraria is the taxonomy within the so-called *T. cruzi* superclade. Its members have been hypothesized to primarily be parasites of bats (Chiroptera), from which they have expanded into other mammals. Two species, *T. cruzi* Chagas, 1909 and *T. rangeli*, both restricted to the New World, are capable of infecting humans. The majority of known invertebrate vectors of these trypanosomes belong to true bugs (Heteroptera). The *T. cruzi* superclade incorporates two subgenera: *Schizotrypanum* and *Aneza*, as well as several (un)named clades and species complexes [237–239].
    *Trypanosoma wauwau* Lima *et al*., 2015 from *Pteronotus* bats in South America constitutes a potentially novel subgenus, now termed the *T. wauwau* clade. Other members of this clade are unnamed species from the Neotropical bats, *T. janseni* Lopes *et al*., 2018 from inner organs of a Brazilian opossum *Didelphis aurita*, and *T. madeirae* Battos *et al*., 2019 from a Neotropical vampire *Desmodus rotundus*. The other two candidate subclades are represented by *Trypanosoma noyesi* Botero and Cooper, 2016 found in Australian marsupials (woylie, wallabies, kangaroos and possums) and the genetically highly diverse (a complex of species) infecting various African bats with the only described member, *Trypanosoma livingstonei* Teixeira and Camargo, 2013.
    ▪ Subgenus *Schizotrypanum* Chagas, 1909
      Diagnosis: Relatively small trypomastigotes (15–25 μm), typically C- or S-shaped in blood smears with short pointed posterior extremity, a large subterminal kinetoplast and long free flagellum. In mammals, reproduction takes place in form of the intracellular amastigotes.
      Type species: *Trypanosoma cruzi* Chagas, 1909; a causative agent of Chagas disease in humans, transmitted by triatomine bugs (e.g. *Triatoma*, *Rhodnius*) [240].
      Note: *Trypanosoma cruzi* (also known as *T. cruzi cruzi* or *T. cruzi sensu* *stricto*) has a very high molecular and phenotypic heterogeneity, reflected by the existence of seven genetically distinct lineages (or discrete typing units, DTUs) termed TcI–TcVI and Tcbat [241]. An impartial comparison of this conspicuous genetic diversity, which corresponds very well with life cycles, clinical manifestations and host specificity, with the situation of the *T. brucei* complex, reveals a striking and untenable discrepancy between these two key species complexes. While in the *T. brucei* complex, five DTUs have the status of five different (sub)species (see below), the *T. cruzi* complex has so far not been split into subspecies and sticks to the DTU code. Therefore, we propose that the same system, based on subspecies, should be applied for both species complexes. We urge our colleagues working with *T. cruzi* to implement such a system.
      The current *T. cruzi* complex is accompanied by three (sub)species from bats with typical *Schizotrypanum* morphology: *T. marinkellei* Baker *et al*., 1978 (restricted to South America), *T. dionisii* Bettencourt and França, 1905 (occurring in the Old and New World) and *T. erneyi* Lima and Teixeira, 2012 (found in Africa).
    ▪ Subgenus *Aneza* Özdikmen, 2009 (= *Tejeraia* Añez, 1982 [preoccupied]).
      Diagnosis: Small to medium-size trypomastigotes (25–35 µm) with long pointed posterior extremity, medium subterminal kinetoplast and long free flagellum, are all similar to the subgenus *Herpetosoma*. At least, some species (e.g. *T. rangeli*) produce metacyclic stages in the salivary glands of its triatomine bug vectors, and therefore are not strictly speaking stercorarians (although transmission via faeces also occurs).
      Type species: *Trypanosoma rangeli* Tejera, 1920.
      Note: *T. rangeli* is restricted to South America and has a wide mammalian host range including humans, for which it is non-pathogenic; the only known vectors are triatomine bugs of the genus *Rhodnius*. While another member of the subgenus, *T. conorhini* Donovan, 1909, is found worldwide in rats and Indonesian primates and is transmitted by triatomine bug *T. vespertilionis* Edmond and Etienne Sergent, 1905 infecting bats is widely distributed in Africa and Europe, where it is transmitted by *Cimex* spp. Sequence data are also available for *T. teixeirae* Barbosa *et al*., 2016 found in the blood of Australian flying foxes.
  ○ ‘mammalian subgenera (salivaria)’ (monophyletic)
    ▪ Subgenus *Duttonella* Chalmers, 1908
      Diagnosis: Small to medium-size trypomastigotes (21–26 µm) with large and usually terminal kinetoplast, small to rounded posterior extremity and long free flagellum. Development in the insect vector is confined to the proboscis and the adjacent cibarial pump.
      Type species: *Trypanosoma vivax* Ziemann, 1905; a causative agent of souma in cattle and other ungulates in Africa and South America (following its import from western Africa).
      Note: Although only one described species has been formally assigned to this subgenus, phylogenetic analyses revealed a complex of species related to *T. vivax* [242]. Previously described *T. uniforme Bruce *et al*., 1911 and *T. vivax ellipsiprymni Keymer, 1969, termed ‘*T. vivax*-like’ may be a part of this complex.
    ▪ Subgenus *Nannomonas* Hoare, 1964
      Diagnosis: Small trypomastigotes (12–20 μm) with medium-sized subterminal marginal kinetoplast, blunt posterior extremity and free flagellum either absent or very short. Vector development takes place in the midgut and proboscis.
      Type species: *Trypanosoma congolense* Broden, 1904; a causative agent of nagana in cattle and other ungulates.
      Note: *T. congolense* is further split into three types/‘subspecies’ (Savannah, Forest and Kilifi) that are, arguably, sufficiently different to warrant species status due to different virulency for domestic animals. Other species infect ungulates and monkeys (*T. simiae* Bruce *et al*., 1909 which is represented by two ‘good’ species) or were detected in tsetse flies like *T. godfreyi* McNamara, Mohammed and Gibson, 1994 and several unnamed species [243,244].
    ▪ Subgenus *Pycnomonas* Hoare, 1964
      Diagnosis: Small trypomastigotes (8–20 µm) with very short pointed posterior extremity, small subterminal kinetoplast and short free flagellum. Vector development takes place in the midgut and salivary glands of tsetse flies (*Glossina* spp.).
      Type species: *Trypanosoma suis* Ochmann, 1905; a causative agent of chronic porcine trypanosomiasis, infects Suidae in sub-Saharan Africa.
      Note: Although this subgenus contains only one described species, two unnamed species were found in tsetse flies [243] and in a wide variety of domestic and free-living ungulates [244].
    ▪ Subgenus *Trypanozoon* Lühe, 1906
      Diagnosis: Pleomorphic trypomastigotes represented by long slender (mean length 30 μm) with long free flagellum and short stumpy forms (mean length 18 µm) with no free flagellum; both have a small subterminal kinetoplast.
      Type species: *Trypanosoma brucei* Plimmer and Bradford, 1899.
      Note: According to the life cycle, transmission mode, vectors, vertebrate hosts and clinical manifestations, five (sub)species are recognized and widely accepted: *Trypanosoma brucei brucei* (in ungulates, the causative agent of nagana in cattle; transmitted by tsetse that restrict its distribution to sub-Saharan Africa), *T. brucei rhodesiense* (causative agent of acute sleeping sickness in humans; game animals and livestock are primary reservoir; vectored by tsetse, sub-Saharan Africa), *T. brucei gambiense* (chronic sleeping sickness in humans; some domestic animals are reservoir; vectored by tsetse, sub-Saharan Africa), *T. brucei evansi* (causative agent of trypanosomiasis in camels, horses, cattle, buffalo, dogs and pigs, called surra in Africa and Asia and murrina in South America; transmitted mechanically by blood-sucking insects and vampires) and *T. brucei equiperdum* (causes dourine in horses in Asia, Africa, South America and Europe; transmitted sexually). The latter two subspecies are closely related and are unique in being so-called petite mutants of *T. brucei* [145].
  ○ ‘other terrestrial subgenera’ (paraphyletic)
    ▪ Subgenus *Australotrypanum* Votýpka and Kostygov, subgen. nov.
      Diagnosis: Morphologically highly variable trypomastigotes in the blood of marsupials and bats in Australia. Defined by 18S rRNA-based phylogenetic analyses.
      Type species: *Trypanosoma copemani* Austen, Jefferies, Friend, Ryan, Adams and Reid, 2009, here designated.
      Etymology: The generic name refers to the origin from Australian mammals.
      Note: A distinct monophyletic clade composed of *T. copemani*, *T. gilletti* McInnes *et al*., 2011, and *T. vegrandis* Thompson *et al*., 2013 that infect marsupials and bats (in case of *T. copemani*) [245–248]. They seem to be transmitted by ticks [249] and have been implicated in the decreased survival of koalas (*Phascolarctos cinereus*) [248]. *T. copemani* exhibits polymorphic ‘slender’ and ‘broad’ trypomastigote stages in the bloodstream [245] and intracellular amastigotes [250]. Sphaeromastigotes, amastigotes and promastigotes were present *in vitro*. On the other hand, *T. vegrandis* is believed to be the smallest species formally described from mammals, with trypomastigotes below 10 μm of length [247]. *Trypanosoma gilletti*, described from koalas based on 18S rRNA sequences only, is 50 µm long [246].
    ▪ Subgenus *Crocotrypanum* Votýpka and Kostygov, subgen. nov.
      Diagnosis: Large striated trypomastigotes (up to 100 µm) occurring in very small numbers in peripheral blood of crocodiles and caimans in the Neotropic and Afrotropic. The conspicuous undulating membrane forms a well-marked frill along the edge of the cell and continues to free flagellum. In tsetse flies (*Glossina*) and horse flies (Tabanidae), epimastigotes and promastigotes develop in the midgut and hindgut; transmission occurs via contaminative way. Defined by 18S rRNA-based phylogenetic analyses.
      Type species: *Trypanosoma grayi* Novy, 1906, here designated.
      Etymology: The generic name refers to the fact that trypanosomes come from hosts of the order Crocodilia.
      Note: *T. grayi* transmitted by tsetse [251–253] clusters together with three recently described species, *T. terena* Teixeira and Camargo, 2013, *T. ralphi* Teixeira and Camargo, 2013 and *T. kaiowa* Teixeira and Camargo, 2019 transmitted by insect vectors [254,255], into a strongly supported monophyletic clade [255]. Based on morphology, *T. cecili Lainson, 1977 could also belong to this subgenus. All described crocodilian trypanosomes form the monophyletic crocodilian clade (subgenus *Crocotrypanum*) of the terrestrial lineage and are transmitted by insect vectors. *Trypanosoma clandestinus* Teixeira and Camargo, 2016, transmitted among caimans by leeches, is not related to this group and is nested within the aquatic lineage (subgenus *Haematomonas*) [255].
    ▪ Subgenus *Squamatrypanum* Votýpka and Kostygov, subgen. nov.
      Diagnosis: Morphologically variable medium to large-sized trypomastigotes with multi-folded undulating membrane including free flagellum and kinetoplast located near the nucleus. Defined by 18S rRNA-based phylogenetic analyses.
      Type species: *Trypanosoma scelopori* Ayala, 1970, here designated.
      Etymology: The unusual combination of hosts (see below) was used for the subgenus name combining the Latin name of reptiles (Squamata) and mammals (Mammalia).
      Note: This clade brings together trypanosomes from diverse hosts, namely lizards, snakes, rodents and marsupials. Based on their morphology, *T. lainsoni* Naiff and Barrett, 2013 from rodents and *T. freitasi* Rêgo, Magalhães and Siqueira, 1957 from marsupials used to belong to the subgenus *Megatrypanum*; however, this taxonomic classification does not reflect their phylogenetic position. While *T. varani* Wenyon, 1908 described from a Nile monitor lizard (*Varanus niloticus*) in Sudan [256] and later found in a Ghanaian ball python (*Python reginus*) [257] represents the only Afrotropical species, other three species within this subgenus were described in American reptiles: *T. serpentis* Viola *et al*., 2009 from Brazilian snake *Pseudoboa nigra* [258], *T. scelopori* Ayala, 1970 from North American western fence lizard (*Sceloporus occidentalis*) [259] and *T. cascavelli* Pessôa and Da Biasi, 1971 from a South American rattlesnake (*Crotalus durissus*) [260]. The latter species also survives in the blood of Neotropical marsupials [261]. *T. freitasi* and *T. gennarii* Marcili, 2017, were described from *Didelphis* and *Monodelphis* opossums, respectively [262,263], yet their host spectrum is even broader [261]. Finally, *T. lainsoni*, originally described from Amazonian rodents [264], can infect South American marsupials and bats [261].
  ○ ‘incertae sedis species'
    Note: Three species—*Trypanosoma irwini* McInnes *et al*., 2009, *T. pestanai* Bettencourt and Franca, 1905 and *T. terrestris* Marcili, 2013—do not fall into any of the above-listed subgenera and constitute separate branches.
    *Trypanosoma irwini* from Australian koala (*Phascolarctos cinereus*), with middle-sized (approx. 40 µm) trypomastigotes with prominent kinetoplast, undulating membrane, pointed posterior end and long free flagellum [265], is closely related to the avian trypanosomes of the subgenus *Ornithotrypanum* [265,266].
    *Trypanosoma pestanai* from Eurasian badgers was, based on morphology (middle-sized trypomastigotes approx. 35 µm long, subterminal kinetoplast) [267,268], associated with the subgenus *Megatrypanum*, yet phylogenetic analyses indicate its affiliation rather with the subgenus *Australotrypanum* [266,269].
    Finally, *T. terrestris* infecting South American lowland tapir (Perissodactyla) is not closely related to any subgenera [270].
  ▪ Subfamily Leishmaniinae Maslov and Lukeš, 2012. Group identified by 18S rRNA and GAPDH gene-based phylogenies. Includes the monoxenous genera *Borovskyia*, *Crithidia*, *Leptomonas*, *Lotmaria*, *Novymonas* and *Zelonia*, as well as the dixenous genera *Endotrypanum*, *Porcisia* and *Leishmania* [271].
    ○ Infrafamily Crithidiatae Kostygov and Yurchenko, 2017. The clade comprises genera *Crithidia*, *Leptomonas* and *Lotmaria*, which currently cannot be reliably separated from each other [271].
      ▪ Genus *Leptomonas* Kent, 1880. Parasites of the gut of invertebrates; promastigotes as the only motile form [94] (plate D, 42).
        Type species: *Leptomonas butschlii* Kent, 1880.
        Note: The type species parasitizes nematodes and does not belong to Trypanosomatidae [97], all other species are parasites of insects [91].
      ▪ Genus *Crithidia* Léger, 1902. Parasites of the gut of insects (dipterans, heteropterans, hymenopterans); choanomastigotes as the only motile form [94] (plate D, 41).
        Type species: *Crithidia fasciculata* Léger, 1902.
      ▪ Genus *Lotmaria* Evans and Schwarz, 2014. The clade comprising *Lotmaria passim* and related species, based on the concatenation of 18S rRNA and gGAPDH genes; promastigotes in the gut of bees [272] (plate D, 43).
        Type species: *Lotmaria passim* Schwarz, 2014. Monotypic (see note).
        Note: Only the type species is currently assigned to this genus. The referred tree topology is strongly gGAPDH-dependent, prone to artefacts due to compositional bias in nucleotide sequences of this gene [103,273–275].
○ Infrafamily Leishmaniatae Maslov and Lukeš, 2012. Comprises the dixenous genera of the subfamily along with *Novymonas*, *Zelonia* and *Borovskyia* [271].
  ▪ Genus *Leishmania* Ross, 1903. Dixenous parasites of mammals and reptiles infecting cells of the mononuclear phagocyte system, where they multiply as amastigotes. Widely distributed in tropical and subtropical regions. In mammals, depending on the preferred type(s) of phagocytes, they cause different clinical forms of the disease: cutaneous, mucocutaneous and visceral. Transmitted predominantly by phlebotomine sandflies (Diptera: Psychodidae: Phlebotominae), in whose gut they develop as promastigotes [127] (plate D, 49,50).
    Type species: *Piroplasma donovani* Laveran and Mesnil, 1903 (= *Leishmania donovani*).
    • Subgenus *Leishmania* Ross, 1903. Cause cutaneous and visceral forms of leishmaniasis in mammals. Distributed in Africa, Eurasia and Americas. Transmitted by sandflies, in which they develop in the midgut [127].
      Type species: same as for the genus.
    • Subgenus *Viannia* Lainson and Shaw, 1987. Cause cutaneous and mucocutaneous forms of leishmaniasis in mammals; restricted to South America; transmitted by sandflies, in which they develop in the midgut and hindgut [127].
      Type species: *Leishmania braziliensis* Vianna, 1911.
    • Subgenus *Sauroleishmania* Ranque, 1973. Live in the blood cells of lizards and snakes, predominantly in the mononuclear phagocyte system, but reported also from erythrocytes and thrombocytes [276]. Transmitted by sandflies, in which they develop in the hindgut [127].
      Type species: *Leishmania tarentolae* Wenyon, 1920.
    • Subgenus *Mundinia* Shaw, Camargo and Teixeira, 2016. Cause cutaneous and visceral leishmaniases in mammals [125,277]; recorded on all continents except Antarctica; transmitted by biting midges [126,278].
      Type species: *Leishmania enriettii* Muniz and Medina, 1948.
    ▪ Genus *Porcisia* Shaw, Camargo and Teixeira, 2016. *Leishmania*-like dixenous flagellates parasitizing skin and visceral organs of porcupines as intracellular amastigotes; transmitted by phlebotomine sand flies as promastigotes [125,279,280] (plate D, 46).
      Type species: *Leishmania hertigi* Herrer, 1971 (= *Porcisia hertigi*).
    ▪ Genus *Endotrypanum* Mesnil and Brimont, 1908. May represent a mixture of two distinct dixenous taxa, one of which is defined morphologically (intraerythrocytic trypomastigotes and/or epimastigotes in sloths) and another phylogenetically (*Leishmania*-like flagellates related to former *L. herreri* and parasitizing skin and visceral organs of various mammals as amastigotes [125,271,281]. The latter is transmitted by phlebotomine sand flies as promastigotes [125] (plate D, 48).
      Type species: *Endotrypanum schaudinni* Mesnil and Brimont, 1908.
    ▪ Genus *Novymonas* Kostygov and Yurchenko, 2020. Monoxenous, insect host unknown; promastigotes and choanomastigotes; the only known species bears multiple vacuole-enclosed β-proteobacterial cells in the cytoplasm [275].
      Type species: *Novymonas esmeraldas* Votýpka, Kostygov, Maslov and Lukeš, 2020*.* Monotypic (plate D, 44).
    ▪ Genus *Zelonia* Shaw, Camargo and Teixeira, 2017. Monoxenous; promastigotes parasitizing true bugs and dipterans; represent a distinct lineage that cannot be associated with any other described genus [125].
      Type species: *Leptomonas costaricensis* Yurchenko, Lukeš, Jirků, Zeledon and Maslov, 2006 (= *Zelonia costaricensis*) (plate D, 45).
    ▪ Genus *Borovskyia* Kostygov and Yurchenko, 2017. Monoxenous; parasites of true bugs; only promastigotes are known; represents the earliest branch within Leishmaniatae [271].
      Type species: *Leptomonas barvae* Maslov and Lukeš, 2010 (= *Borovskyia barvae*)*.* Monotypic.
  ▪ Subfamily Herpetomonadinae Alexeieff, 1911, stat. nov., emend. Kostygov and Yurchenko (= Phytomonadinae Yurchenko, Kostygov, Votýpka and Lukeš, 2015; unavailable name).
    Diagnosis: Clade of monoxenous parasites of insects and dixenous parasites of insects and plants defined by phylogenetic analyses based on 18S rRNA and gGAPDH gene sequences; promastigotes or choanomastigotes are dominant morphotypes; may also form opisthomastigotes, opisthomorphs and endomastigotes. Arginase absent.
    Type genus: *Herpetomonas* Kent, 1880.
    Note: *Herpetomonas* was designated as a type genus of the subfamily Phytomonadinae [282], making the latter unavailable according to article 11.7.1.1 of ICZN. At the same time, the name Herpetomonadidae, a synonym of Trypanosomatidae Doflein, 1901 at the family level, is available as a name of the subfamily (with the ending -inae), being the only suitable one for a clade containing its type genus.
  ▪ Genus *Herpetomonas* Kent, 1880. Monoxenous; parasites of dipterans, true bugs, fleas, cockroaches and ciliates; polymorphic: predominant promastigotes varying in size and shape as well as non-mandatory opisthomastigotes, opisthomorphs and endomastigotes [106,282,283] (plate D, 51,52).
    Type species: *Bodo muscarum* Leidy, 1856 (= *Herpetomonas muscarum*)*.*
  ▪ Genus *Lafontella* Kostygov and Yurchenko, 2015. Monoxenous; parasitic in the gut of flies; promastigotes, opisthomastigotes and long endomastigotes with elongated coiled flagellum [282,284].
    Type species: *Herpetomonas mariadeanei* Yoshida, Freymuller and Wallace, 1978 (= *Lafontella mariadeanei*). Monotypic.
  ▪ Genus *Phytomonas* Donovan, 1909 emend. Kostygov.
    Diagnosis: long (often twisted) promastigotes and endomastigotes; most species alternate between plants and phytophagous true bugs, some switched to predatory bugs and became secondary monoxenous; obligate development in salivary glands [103,119,122,123] (plate D, 53,54).
    Type species: *Leptomonas davidi* Lafont, 1909 (= *Phytomonas davidi*).

▪ Subfamily Strigomonadinae Votýpka, Yurchenko, Kostygov and Lukeš, 2014. Monoxenous, with several apomorphic traits: single β-proteobacterial endosymbiont not enclosed in a vacuole, extensively branched mitochondrion disrupting subpellicular corset of microtubules, rudimentary paraflagellar rod [274].
  ▪ Genus *Angomonas* Souza and Corte-Real, 1991. Monoxenous parasites in the gut of blowflies; choanomastigotes and opisthomorphs; kinetoplast nearly rectangular, with kinetoplast minicircles greater than 4 kb [285].
    Type species: *Crithidia deanei* Carvalho, 1973 (= *Angomonas deanei*) (plate D, 56).
  ▪ Genus *Strigomonas* Lwoff and Lwoff 1931. Monoxenous parasites of the gut of dipterans and true bugs; polymorphic: epimastigotes and trypomastigotes or choanomastigotes and opisthomorphs; kinetoplast lens-shaped, usually with only one side convex and another flat or concave; minicircles less than 3 kb [285].
    Type species: *Strigomonas oncopelti* Lwoff and Lwoff, 1931 (plate D, 58).
  ▪ Genus *Kentomonas* Votýpka, Yurchenko, Kostygov and Lukeš, 2014. Monoxenous parasites of the gut of flies; mitochondrial branches press on the plasmatic membrane forming ridges on the cell surface; kinetoplast nearly rectangular [274].
    Type species: *Kentomonas sorsogonicus* Votýpka and Lukeš, 2014. Monotypic (plate D, 57).
▪ Subfamily Blastocrithidiinae Votýpka, Yurchenko and Lukeš, 2021. A well-supported monophyletic group (as judged by the analyses based on 18S rRNA gene) of monoxenous trypanosomatids inhabiting the gut of true bugs (Heteroptera) [286].
  ▪ Genus *Blastocrithidia* Laird, 1959. Monoxenous parasites of the gut of true bugs; epimastigotes form resistant cyst-like straphangers [94]; some members have non-canonical genetic code with all three stop codons coding for amino acids [287] (plate D, 63).
  Type species: *Crithidia gerridis* Patton, 1908 (= *Blastocrithidia gerridis*).
  ▪ Genus *Obscuromonas* Votýpka and Lukeš, 2021. In 18S rRNA-based phylogenies, a sister group to *Blastocrithidia*; monoxenous parasites in different organs of heteropterans; some members produce cyst-like straphangers [286] (plate D, 64).
    Type species: *Obscuromonas modryi* Votýpka and Lukeš, 2021*.*
▪ Subfamily Blechomonadinae Votýpka and Suková, 2013. A clade comprising the genus *Blechomonas* according to phylogeny inferred using 18S rRNA and gGAPDH genes [288].
  ▪ Genus *Blechomonas* Votýpka and Suková, 2013. Monoxenous parasites in the gut of fleas; promastigotes, choanomastigotes and amastigotes significantly varying in size [288] (plate D, 66).
    Type species: *Blechomonas ayalai* Votýpka and Suková, 2013.
▪ Subfamily Paratrypanosomatinae Votýpka and Lukeš, 2013. The earliest-branching lineage within the family as judged by the phylogenies inferred using 18S rRNA and multiple protein-coding genes [289]. Single genus.
  ▪ Genus *Paratrypanosoma* Votýpka and Lukeš, 2013. Monoxenous parasites of the gut of dipterans; promastigotes; well-developed oral apparatus with cytostome on the outer cell surface [290].
    Type species: *Paratrypanosoma confusum* Votýpka and Lukeš, 2013. Monotypic (plate D, 62).
• Genera not assigned to subfamilies
  ▪ Genus *Jaenimonas* Votýpka and Hamilton, 2020. Distinct monoxenous lineage in 18S rRNA and gGAPDH gene-based phylogenies; parasite of the gut of fruit flies; monotypic [112].
    Type species: *Jaenimonas drosophilae* Votýpka and Hamilton, 2020 (plate D, 60).
  ▪ Genus *Vickermania* Kostygov and Yurchenko, 2020. Monoxenous parasites of the gut of flies; promastigotes with two anteriorly oriented flagella of unequal length, typically attached to each other and separated during cell division; uniflagellate cells appear only shortly after division; flagellar tips have rounded or elongated apex and lateral extensions; large and loosely arranged kDNA [291].
    Type species: *Herpetomonas muscarum ingenoplastis* Rogers and Wallace, 1971 (= *Vickermania ingenoplastis*) (plate D, 65).
  ▪ Genus *Sergeia* Svobodová *et al*., 2007. Distinct monoxenous lineage in 18S rRNA and gGAPDH gene-based trees; parasite of the gut of biting midges; promastigotes as the only motile stage [292].
    Type species: *Sergeia podlipaevi* Svobodová *et al*., 2007. Monotypic (plate D, 59).
  ▪ Genus *Wallacemonas* Kostygov and Yurchenko, 2014. Distinct monoxenous lineage in 18S rRNA and gGAPDH gene-based trees; parasites of dipterans and true bugs; promastigotes as well as non-mandatory opisthomorphs and endomastigotes [293,294].
    Type species: *Leptomonas collosoma* Wallace, Clark, Dyer and Collins, 1960 (= *Wallacemonas collosoma*) (plate D, 61).
• Trypanosomatidae *incertae sedis* (none is available in culture)
  ▪ Genus *Cercoplasma* Roubaud, 1911. Monoxenous; in the gut of flies; epimastigotes and trypomastigotes; flagellum without free part, accompanied by a filamentous cell processus [295].
    Type species: *Cercoplasma caulleryi* Roubaud, 1911. Monotypic.
  ▪ Genus *Malacozoomonas* Nicoli, Penaud and Timon-David, 1971. Monoxenous; in the gut and hepatopancreas of molluscs; promastigotes and amastigotes [296].
    Type species: *Herpetomonas patellae* Porter, 1914 (= *Malacozoomonas patellae*).
  ▪ Genus *Nematodomonas* Nicoli, Penaud and Timon-David, 1971. Monoxenous; in the gut of nematodes; promastigotes only [297].
    Type species: *Nematodomonas goodeyi* Nicoli, 1971. Monotypic.
  ▪ Genus *Rhynchoidomonas* Patton, 1910 (= *Cystotrypanosoma* Roubaud, 1911). Monoxenous; in the gut and Malpighian tubules of flies; trypomastigotes without free flagellum and conspicuous undulating membrane; cyst-like amastigotes observed in some species [94,298];
    Type species: *Rhynchomonas luciliae* Patton, 1910 (= *Rhynchoidomonas luciliae*).
• Protists erroneously assigned to Kinetoplastea (none is available in culture)
  ▪ Genus *Trypanophis* Keysselitz, 1904 emend. Kostygov.
    Diagnosis: Parasites of the gastrovascular cavity of siphonophores; biflagellate, with striated rootlet, short free anterior flagellum, long posterior flagellum attached to the cell body and situated in shallow longitudinal groove; subpellicular microtubules present only under groove; membranous sacs under plasmalemma; micropores; elongated mitochondrion parallel to the flagellar groove, with tubular cristae, possesses anterior dilation (‘kinetoplast’) with multiple osmiophilic bodies; no traces of oral apparatus [299].
    Type species: *Trypanosoma grobbeni* Poche, 1903 (= *Trypanophis grobbeni*).
    Note: Assignment of this genus to kinetoplastids is not justified, since its ‘kinetoplast’, as judged by Feulgen staining, does not contain DNA. The presence of cortical membranous sacs, micropores and a striated rootlet [299] strongly suggest that this flagellate is a member of Alveolata Cavalier-Smith, 1991.
• Kinetoplastea *incertae sedis*
  ▪ Genus *Bordnamonas* Larsen and Patterson, 1990. Free-living, solitary, with pliable body; biflagellate, anterior flagellum forms arc extending in front of the cell, while posterior flagellum is trailing; phagotrophic, cytostome is anterior to flagella [300]; fine structure and type of kinetoplast unknown.
    Type species: *Bordnamonas tropicana* Larsen and Patterson, 1990. Monotypic (plate A, 7).
    Note: although many features of the genus are reminiscent of kinetoplastids, it was also considered to be a stramenopile [182,301].
  ▪ Genus *Cephalothamnium* Stein, 1878. Ectocommensal on freshwater copepods; biflagellate, both flagella with mastigonemes, the posterior flagellum attached to cell body; forms sedentary colonies with up to 30 cells attached to secreted stalk by the distal end of the recurrent flagellum; large prokinetoplast; subpellicular microtubules only in the anterior part; phagotrophic, with apical cytostome and funnel-shaped cytopharynx [61].
    Type species: *Cephalothamnium cyclopum* Stein, 1878. Monotypic.
  ▪ Genus *Desmomonas* Williams, 1999. Parasitic in turbellarian parenchyma, either free or attached to host cell masses via anterior processus with desmosomes; biflagellate, both flagella unattached to the cell body, oriented posteriorly and lacking prominent paraflagellar rod; corset of subpellicular microtubules has breaches allowing body shape changes; no cytostome detected (osmotrophic); compact mitochondrion with polykinetoplast distant from the flagellar base; microneme-like osmiophilic bodies [60].
    Type species: *Desmomonas prorhynchi* Williams, 1999. Monotypic.
  ▪ Genus *Jarrelia* Poynton, Whitaker and Heinrich, 2001. Parasite of blowhole mucus of pygmy sperm whale; biflagellate, posterior flagellum forms undulating membrane and can attach to host material by its tip; polykinetoplastic; osmotrophic [87].
    Type species: *Jarrellia atramenti* Poynton, Whitaker and Heinrich, 2001. Monotypic.
    Note: previously assigned to Parabodonida based on superficial resemblance with *Trypanoplasma* [3]; however, no reliable evidence supporting such an assignment is available.
  ▪ Genus *Lamellasoma* Davis, 1947. Parasite of fish gills; uniflagellate, single flagellum oriented posteriorly and attached to cell body; type of kinetoplast uncertain; epibiotic bacteria on the surface [81].
    Type species: *Lamellasoma bacillaria* Davis, 1947. Monotypic.
    Note: May represent an unusual species of piscine *Cryptobia* with a very short or completely reduced anterior flagellum [81].

**Plate D.**
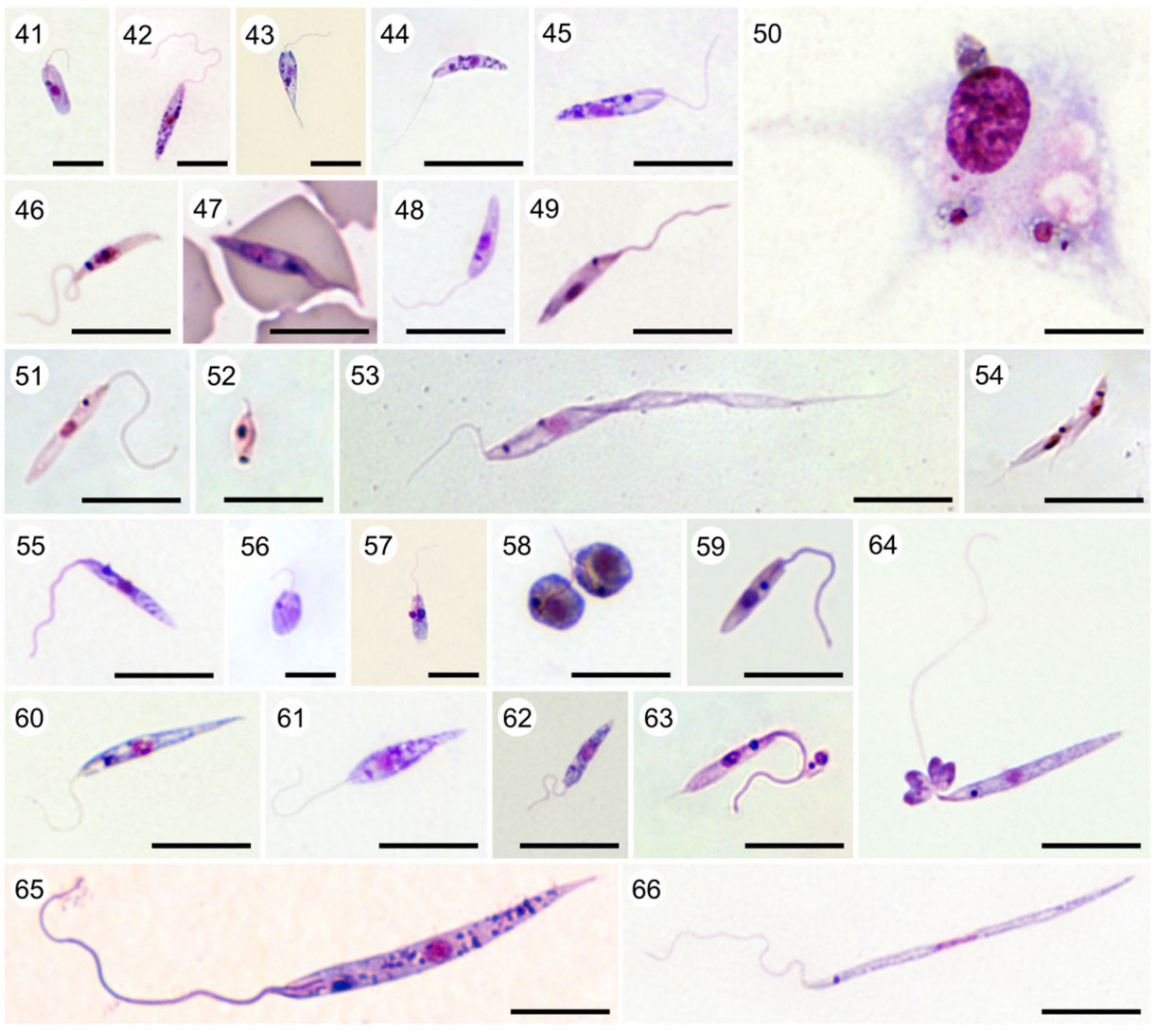
Trypanosomatids other than *Trypanosoma*. Light micrographs of Giemsa-stained (41) *Crithidia thermophila* (culture) (42) *Leptomonas seymouri* (culture); (43) *Lotmaria passim* (culture); (44) *Novymonas esmeraldas* (culture); (45) *Zelonia costaricensis* (culture); (46) *Porcisia hertigi* (culture) (provided by Jovana Sádlová); (47) *Endotrypanum* sp. ex sloth; (48) *Endotrypanum monterogeii* (culture); (49) *Leishmania major*, metacyclic promastigote (culture) (provided by JS); (50) *L. major*, amastigotes in a macrophage (provided by Tereza Leštinová); (51) *Herpetomonas nabiculae*, promastigote (culture) (provided by Marina N. Malysheva and Alexander O. Frolov); (52) *H. nabiculae*, opistomastigote (provided by Marina N. Malysheva and Alaxander O. Frolov); (53) *Phytomonas lipae*, promastigote ex *Coreus marginatus* (provided by Marina N. Malysheva and Alaxander O. Frolov); (54) *P. lipae*, endomastigotes ex *C. marginatus* (provided by Marina N. Malysheva and Alaxander O. Frolov); (55) *Lafontella* sp. (culture); (56) *Angomonas deanei* (culture) (provided by Anna I. Ganyukova); (57) *Kentomonas sorsogonicus* (culture); (58) *Strigomonas oncopelti* (culture); (59) *Sergeia podlipaevi* (culture); (60) *Jaenimonas drosophilae* (culture); (61) *Wallacemonas collosoma* (culture); (62) *Paratrypanosoma confusum* (culture); (63) *Blastocrithidia frustrata* (culture); (64) *Obscuromonas oborniki* (culture); (65) *Vickermania ingenoplastis* (culture); (66) *Blechomonas englundi* (culture). Scale bar, 5 µm (41–43, 56, 57); 10 µm (44–55, 58–66).

## 3. Diplonemea

### 3.1. Biology

The body of knowledge on the biology of diplonemids comprises a large number of environmental 18S rRNA sequences, few cultured and sequenced species, as well as several formally described species lacking sequence data and not available in culture. Until recently, diplonemids have been perceived as a small and unimportant group of euglenozoans. However, deep-sea sampling and extensive metabarcoding surveys in the past two decades uncovered extraordinary diversity of marine planktonic diplonemids [12,302,303]. Their discovery began with the recovery of environmental 18S rRNA sequences from deep-sea planktonic and hydrothermal samples, which together formed a novel well-supported clade, sister to Diplonemidae [304,305]. Subsequently, a diplonemid-focused study of several oceanic regions uncovered considerable diversity within the new clade by amplifying 18S rRNA, designated as deep-sea pelagic diplonemids (DSPD) I clade [302]. The same study identified another small lineage called DSPD II.

A breakthrough came with a V9 region metabarcoding survey by the *Tara* Oceans expedition, which uncovered remarkable abundance and diversity of DSPD in the tropical and subtropical sunlit ocean, expanding the number of potential diplonemid species to over 12 300 [303]. Further analysis of combined datasets from photic and mesopelagic zones identified as many as approximately 45 000 diplonemid species, thus qualifying them among the most species-rich planktonic eukaryotes in the ocean [12]. In the most recent study, extended with smaller datasets from the Arctic, Adriatic Sea and anoxic Cariaco Basin, the number of species increased to approximately 67 000, designating diplonemids as the most diverse and fifth most abundant eukaryotic clade [48]. However, fluorescence *in situ* hybridization studies or those based on the V4 region of 18S rRNA reported significantly lower abundance [306–308].

Analysis of extended *Tara* Oceans datasets identified that 97% of diplonemid diversity is confined to the DSPD I clade, or eupelagonemids, whereas classic diplonemids (or Diplonemidae), hemistasiids and DSPD II accounted for 1% each [12,48]. The distribution of eupelagonemids showed clear depth stratification: although their sequences were recovered from the surface water down to the abyssopelagic zone [309], they are most diverse and abundant in the mesopelagic zone. However, multiple lineages of eupelagonemids show cosmopolitan distribution without a clear biogeographic pattern and a rather weak relation to abiotic factors [310]. Eupelagonemids generally show preference for dark and moderately oxygenated environments, but were occasionally detected under anoxic conditions [48]. It is still not known what drives the high diversity of eupelagonemids given the relative homogeneity of the physico-chemical conditions in the deep ocean, especially in dysphotic and aphotic zones. It has been suggested that different species might use different nutrient resources [302]. Indeed, the co-occurrence analyses showed very few obvious patterns of interaction with other components of marine plankton, among which are positive correlations with parasitic dinoflagellates and stramenopiles as well as with bacteria and bacterivorous stramenopiles, indicating possible bacteriovory and parasitic lifestyle of some eupelagonemids [12] (plate E, 67–76).

Unlike the deep-sea planktonic clades, which inhabit nutrient-poor, dark and cold ocean zones [12,302], classic diplonemids seem to prefer a variety of nutrient-rich environments, such as benthos, coastal surface waters, artificial water bodies and aquaria [311]. Classical diplonemids have been widely considered as benthic organisms [11] apparently due to the sampling bias. Their sequences have been indeed recovered from various benthic environments, including cold anoxic seeps [312], hydrothermal vents [304] and the sea floor [302], in addition to several species that were observed in tropical shallow-water [300,313] and deep-sea sediments [311]. However, diplonemids are also a common component of the plankton in photic layer of the temperate to tropical zones [12]. In addition, their representatives are known from coastal planktonic communities, including *Rhynchopus coscinodiscivorus* [314], *Diplonema breviciliata* [315], *D. papillatum* [316], *D. nigricans* [317], *Lacrimia lanifica*, *Rhynchopus serpens* and *Sulcionema specki* [311]. Classical diplonemids were also frequently isolated from aquaria, such as *D. japonicum*, *D. aggregatum*, *D. ambulator* ATCC 50223, *Rhynchopus humris*, *Flectonema neradi* and *Rhynchopus* ATCC 50230 [311] (plate E, 67–70,72–76).

Diplonemids have been occasionally reported from freshwater ecosystems, such as the case of *Rhynchopus amitus* [318] and *Diplonema ambulator* from a freshwater aquarium [319]. Subsequently, metabarcoding approach identified a low number of diplonemids in geographically isolated deep lakes, such as Baikal [320], and lakes in Japan [321], Switzerland and the Czech Republic [322]. Further systematic metabarcoding screening of lakes using diplonemid-specific primers might uncover so far overlooked diversity of freshwater diplonemids.

The fourth diplonemid clade, Hemistasiidae, is represented by several hundred species, so far found in the photic zone [12]. *Hemistasia*-like flagellates have been frequently detected in coastal waters of North and Baltic seas, Mediterranean, the Sea of Japan and around Australia and Antarctica [313,315,323–325], pointing to their cosmopolitan distribution from cold to tropical regions. All hemistasiids were described from planktonic samples, except for *Artemidia motanka* isolated from an aquarium [311] and a hemistasiid associated with shallow-water sediments [313].

Classic diplonemids and hemistasiids are exclusively heterotrophic organisms, mostly known as eukaryovores, and displaying a wide array of lifestyles, such as ectocommensalism, predation, scavenging and opportunistic endoparasitism. Several species were found to parasitize lobsters, clams [180,326] and plants [319], while others were referred to as epibionts of crabs [327], lobsters [180], plants [300,315,316] and algal biofilms (*Diplonema* sp. 4 ATCC 50232). Another common trophic mode for both groups is predation and/or scavenging on planktonic algae and small invertebrates, including diatoms, dinoflagellates, green algae, prymnesiophytes and copepods [314,315,318,323]. Bacteriovory was described from only two species and seems rather uncommon [300,328].

### 3.2. Taxonomy

Class Diplonemea and order Diplonemida Cavalier-Smith, 1993 (tree E).

**Tree E.**
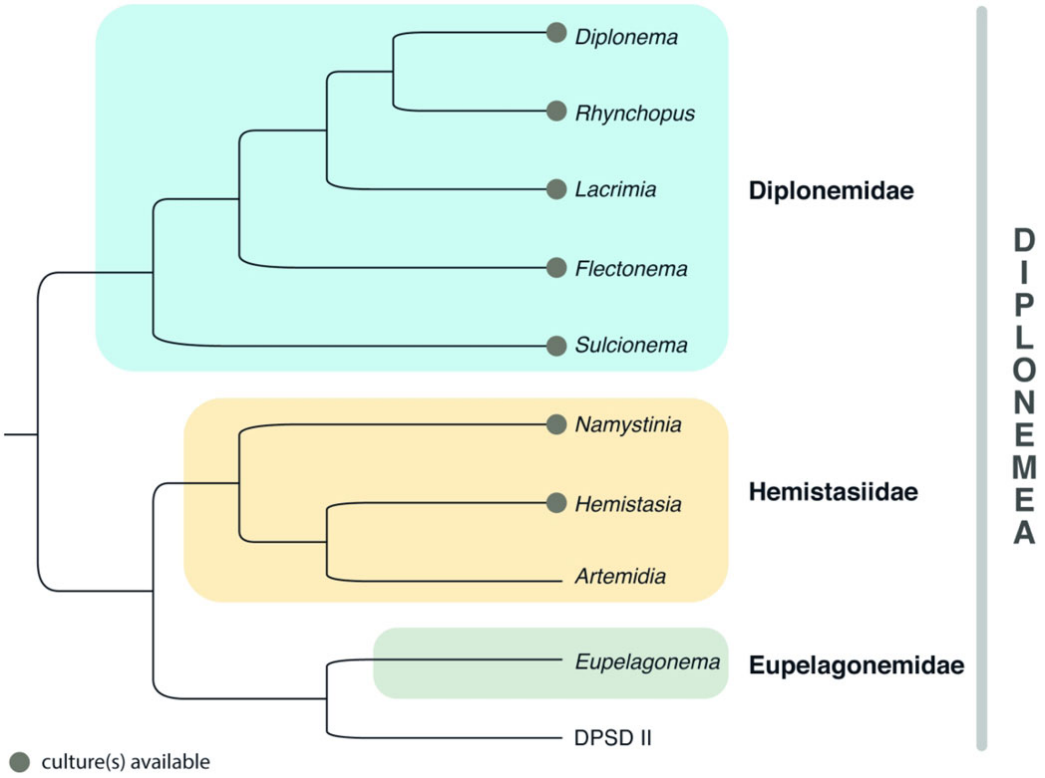
Diplonemids. A tree summarizing phylogenetic reconstructions based on 18S rRNA gene. Circles denote genera with cultured representatives.

Naked colourless biflagellates with apical papilla; subapical flagellar pocket; plasma membrane subtended by dense microtubular corset; peripheral mitochondria with giant lamellar cristae and multiple interspersed DNA aggregates; equally thick flagella; tubular extrusomes in several species; likely phagotrophic feeding.

• Family Diplonemidae Cavalier-Smith, 1993; also known as classic diplonemids with metaboly always present.
  ▪ Genus *Diplonema* Griesmann, 1914. Based on 18S rRNA gene, the genus is paraphyletic [180]; morphological and 18S rRNA discrepancies (*D. papillatum* is 87% different from *D. ambulator*) justify possible division of this genus into two genera. Elongated body with constricted anterior end; equal to subequal flagella; gliding movement and ambulation of flagella in trophic stage; tubular Euglenozoa-type extrusomes and heterodynamic flagella with paraflagellar rods (PFR) in swimming stage (if present) (applies to *Diplonema ambulator*, *D. japonicum* and *D. aggregatum*).
  Type species: *Diplonema* (*Isonema*) *papillatum* Porter, 1973 (short flagella of equal length lacking PFR; nearly apical flagellar pocket; big papilla) (plate E, 67).
  ▪ Genus *Rhynchopus* Skuja, 1948. Flagellar stubs with disorganized axoneme microtubules, concealed inside flagellar pocket and gliding motion in trophic stage; flagella are gradually built in actively gliding cells; long heterodynamic flagella with PFR in swimming stage, anterior flagellum forming a lasso and the posterior one stretched along the body (plate E, 68).
  Type species: *Rhynchopus amitus* Skuja, 1948.
  ▪ Genus *Lacrimia* Tashyreva, Prokopchuk, Horák and Lukeš, 2018. Permanently long subequal flagella with PFR; teardrop-shaped body; big posterior digestion vacuole; rotation movement and oscillating swimming pattern.
  Type species: *Lacrimia lanifica* Tashyreva, Prokopchuk, Horák and Lukeš, 2018 (plate E, 73).
  ▪ Genus *Flectonema* Tashyreva, Prokopchuk, Horák and Lukeš, 2018. Short flagella of equal length; elongated, slender, crooked body reminiscent to *D. ambulator* type; distinguished by the presence of PFR in trophic stage; gliding and rotation motion, swimming absent, dispersal swimming stage not described.
  Type species: *Flectonema neradi* Tashyreva, Prokopchuk, Horák and Lukeš, 2018 (plate E, 74).
  ▪ Genus *Sulcionema* Tashyreva, Prokopchuk, Horák and Lukeš, 2018. Short flagella of equal length, containing PFR; long flat body with conspicuous cytoplasmic inclusions, pleomorphic; writhing motion, but swimming and gliding absent; highly metabolic.
  Type species: *Sulcionema specki* Tashyreva, Prokopchuk, Horák and Lukeš, 2018 (plate E, 69).
• Family Hemistasiidae Cavalier-Smith, 2016. Fast swimming, long flagella with prominent PFR; tubular Euglenozoa-type extrusomes; peripheral lacunae; highly asymmetrical apex with flexible pointy rostrum; metaboly always present; anterior groove; subapically inserted flagella inside a deep invagination; large posterior digestion or lipid vacuole is common; cylindrical to pyriform body; establishment of three genera is justified by substantial differences in 18S rRNA gene [325].
  ▪ Genus *Hemistasia* Griesmann, 1914. Invariable presence of tubular extrusomes; distinguished from related genera by acute rostrum and smaller body.
  Type species: *Oxyrrhis phaeocysticola* Scherffel, 1900 (= *Hemistasia phaeocysticola*) (plate E, 76).
  ▪ Genus *Namystinia* Prokopchuk, Tashyreva and Lukeš, 2019. Broader rostrum; tubular extrusomes only in starved cells; morphologically indistinguishable from *Artemidia.*
  Type species: *Namystinia karyoxenos* Prokopchuk, Tashyreva and Lukeš, 2019 (plate E, 75).
  ▪ Genus *Artemidia* Prokopchuk, Tashyreva and Lukeš, 2019. Broader rostrum; invariable presence of tubular extrusomes.
  Type species: *Artemidia motanka* Prokopchuk, Tashyreva and Lukeš, 2019 (plate E, 70).
• Family Eupelagonemidae Okamoto and Keeling, 2019. Formerly known as ‘deep sea pelagic diplonemids 1’ (DSPD I), possibly non-metabolic.
  ▪ Genus *Eupelagonema* Okamoto and Keeling, 2019. Elongated elliptical body, round on one end and constricted on the other (plate E, 71).
  Type species: *Eupelagonema oceanica* Okamoto and Keeling, 2019.
  Note: DSPD II (deep sea pelagic diplonemids II)—small planktonic clade, well-supported phylogenetically, known exclusively from sequences of the V9 region of 18S rRNA [12], without cultured or formally described representatives; morphology and ultrastructure not known.

**Plate E.**
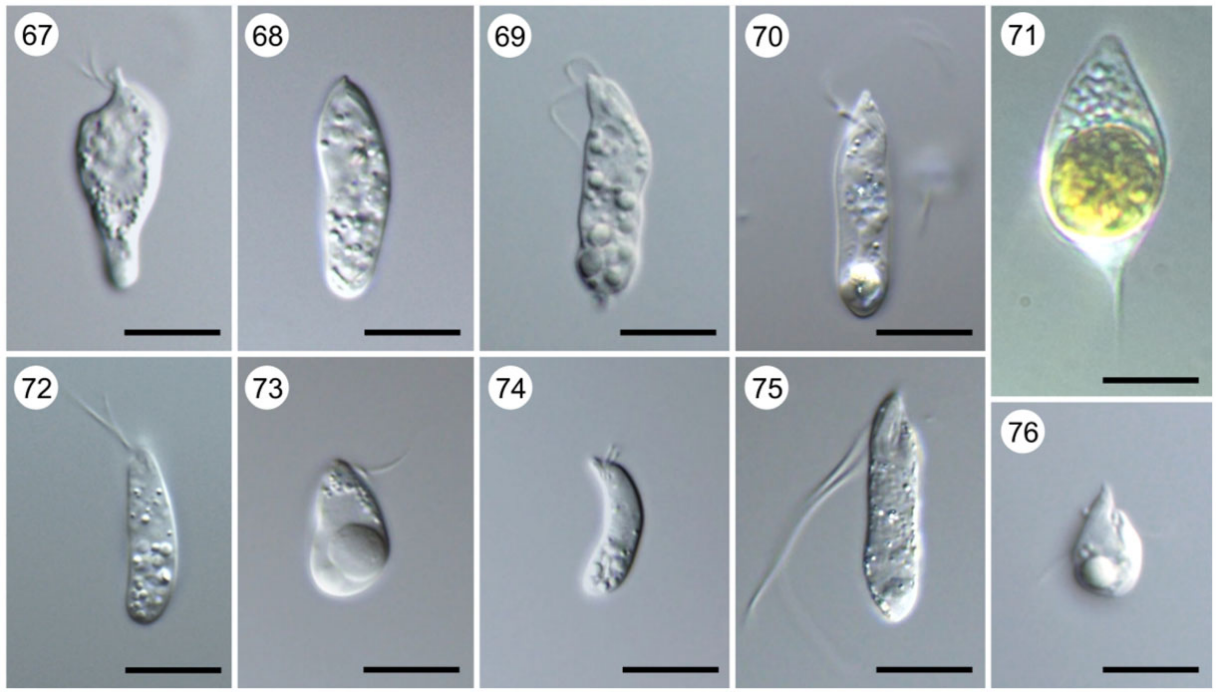
Diplonemids. Light micrographs of cultured (67) *Diplonema papillatum*; (68) *Rhynchopus euleeides*; (69) *Sulcionema specki*; (70) *Artemidia motanka*; (71) Eupelagonemid sp. (provided by Noriko Okamoto and Patrick Keeling); (72) *Diplonema aggregatum*; (73) *Lacrimia lanifica*; (74) *Flectonema neradi*; (75) *Namystinia karyoxenos*; (76) *Hemistasia phaeocysticola*. Scale bar, 10 µm (67–75).

## 4. Euglenida and Symbiontida

### 4.1. Biology

Since most of the 18S rRNA phylogenies placed symbiontids either within euglenids, or as a sister clade to them (e.g. [170,329,330]), they are often regarded as derived euglenids (e.g. [329,331]). For those reasons, here we discuss the biology of euglenids and symbiontids together. However, a recent phylogenomic reconstruction [8] placed the latter group as a sister to diplonemid-kinetoplastid clade (Glycomonada), suggesting that they should be treated as a separate group within Euglenozoa.

Euglenida (plate F, 77–96) and Symbiontida (plate F, 97) inhabit aquatic environments, but they dominate in different ecological niches. Phagotrophic euglenids (plate F, 88–96) are widespread in shallow marine, brackish and freshwater sediments, and are presumably important predators in these ecosystems [42,332,333]. Recently, they have also been reported from deep-sea samples [334]. Osmotrophic (plate F, 82) and phototrophic euglenids (euglenophytes; plate F, 77–81, 83–87) mainly inhabit the water column of freshwater environments. In the temperate zone, euglenophytes are abundant in small eutrophic reservoirs where the water warms up quickly. They might form blooms, including toxic blooms caused by *Euglena sanguinea* [335,336]. In the tropical climate, euglenophyte blooms are also commonly reported, especially from aquaculture ponds [337,338]. Several lineages of typically freshwater genera—*Discoplastis* (plate F, 83), *Phacus* (plate F, 79), *Lepocinclis* (plate F, 78), *Euglena* (plate F, 85) and *Euglenaria*, have been detected in the coastal environments in low abundances [339]. Moreover, some species (*Euglena rustica* and *E. obtusa*) have been reported to migrate vertically in marine sand in coordination with tidal and diurnal cycles. They are usually highly abundant and form green patches in marine sand during low tides [340]. The three earliest-branching lineages of photosynthetic euglenids, *Rapaza* (plate F, 87), *Eutreptia* and *Eutreptiella* (plate F, 86), belong to marine plankton. Although the known diversity of marine species of euglenophytes is low, blooms of Eutreptiales have been reported from eutrophic coastal waters, where they can make up to approximately 46% of the total biomass of the phytoplankton population [341,342]. Symbiontids, inhabiting both shallow and deep anoxic marine sediments, host sulfur-oxidizing or sulfide-oxidizing epsilonproteobacterial epibionts, which detoxify their immediate surrounding environment [41]. They might be a dominant group in certain environments, such as in the protist community associated with bacterial mats in oxygen-depleted sediment in Monterey Bay [343].

**Plate F.**
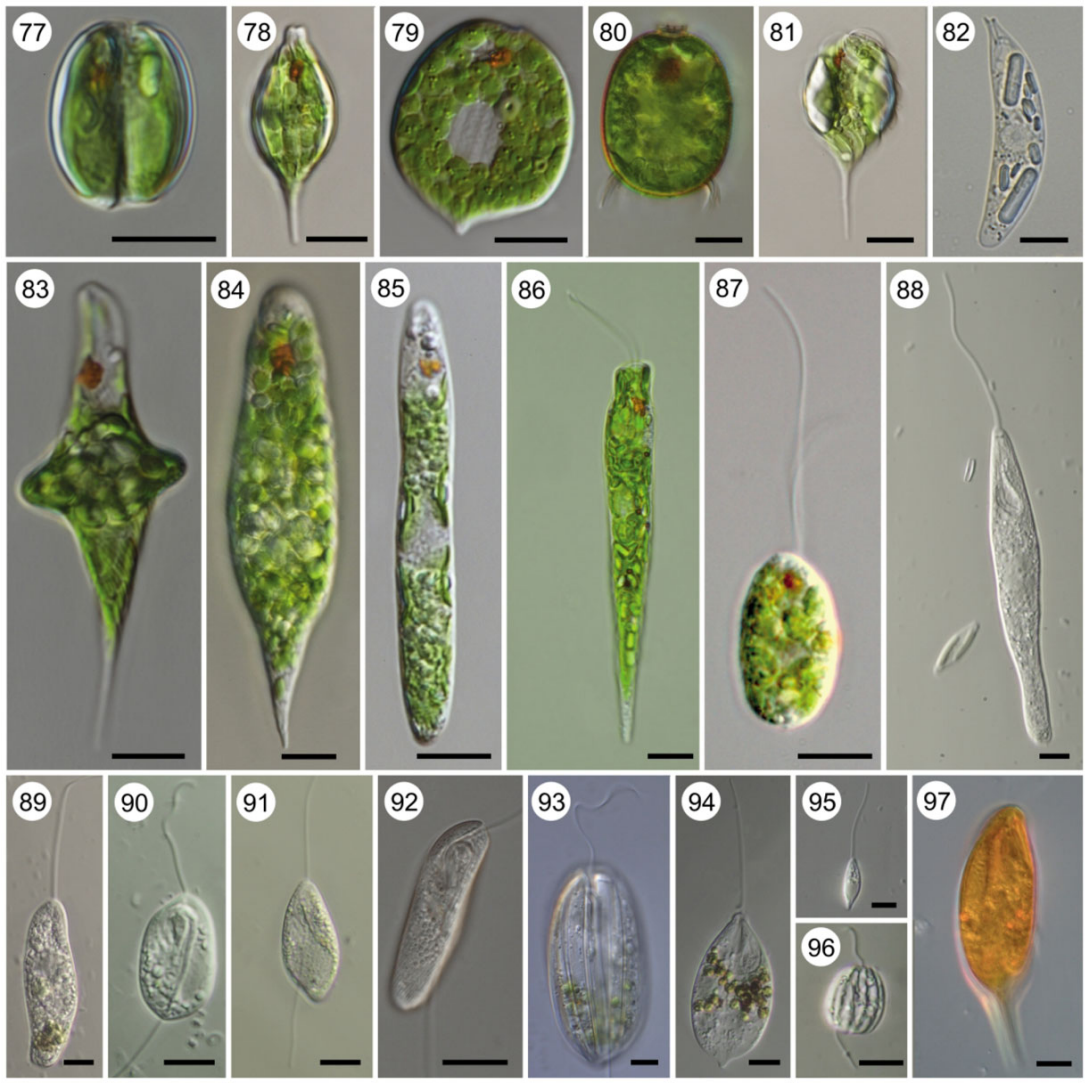
Euglenids. Light micrographs of cultured (77) *Cryptoglena* sp. (provided by Bożena Zakryś); (78) *Lepocinclis autumnalis* (provided by Bozena Zakrys); (79) *Phacus acuminatus* (provided by Bozena Zakrys); (80) *Trachelomonas armata* (provided by Bozena Zakrys); (81) *Monomorphina* sp. (provided by Bozena Zakrys); (82) *Menoidium* sp. (provided by Bozena Zakrys); (83) *Discoplastis spathirhyncha* (provided by Bozena Zakrys); (84) *Euglenaformis proxima* (provided by Bozena Zakrys); (85) *Euglena gracilis* (provided by Bozena Zakrys); (86) *Eutreptiella pomquetensis* (provided by Bozena Zakrys); (87) *Rapaza viridis* (provided by Naoji Yubuki); (88) *Jenningsia* sp. (provided by Gordon Lax); (89) *Peranema* sp. (provided by Gordon Lax); (90) *Anisonema* sp. (provided by Gordon Lax); (91) *Heteronema vittatum* (provided by Gordon Lax); (92) *Dinema* sp. (provided by Gordon Lax); (93) *Olkasia polycarbonata* (provided by Gordon Lax); (94) *Notosolenus ostium* (provided by Gordon Lax); (95) *Sphenomonas teres* (provided by Gordon Lax); (96) *Lentomonas corrugata* (provided by Gordon Lax); (97) *Calkinsia aureus* (provided by Naoji Yubuki). Scale bar, 10 µm (77–97).

It has been demonstrated that both phagotrophic (e.g. *Distigma*) and photosynthetic euglenids (e.g. *Euglena gracilis;* plate F, 85) are remarkably tolerant to various kinds of pollution with heavy metals such as cadmium, chromium or lead, as well as capable to remove these ions from the environment, making these protists potentially suitable for use in bioremediation of heavy metal-rich industrial wastewater [344,345]. The genetic background of heavy metal resistance in euglenids has been examined in detail only in the genus *Peranema.* Interestingly, although the investigated *Peranema* sp. strain exhibited the capability for efficient removal of cadmium from wastewater samples, the study revealed that it possesses genes responsible for resistance to a variety of other heavy metals, but not cadmium [346]. Euglenophytes have also been found in waters polluted with diesel oil [347], phenol [348], herbicides and insecticides [349,350], and can survive in highly radioactive water [351]. Some Euglenophytes are also extremophiles (e.g. *Euglena mutabilis*), as they tolerate very high salinity [352] in extremely acidic environments [353] or in hot mud pools [354].

Euglenids and symbiontids are predominantly free-living, exhibiting a remarkably wide range of nutrition modes, including phagotrophy, osmotrophy and photoautotrophy. Phagotrophic euglenids consume bacteria or microbial eukaryotes, and their prey size correlates with the euglenids' cell size and flexibility. Some phototrophs are capable of pinocytosis, or even phagotrophy of algae in the case of the deep-branching phototroph *Rapaza* [355] (plate F, 87). Symbiontids are marine heterotrophs, presumably phagotrophs, as suggested by their ultrastructure. Additionally, the bacteria on their surface probably exchange metabolites with the hosts' mitochondria-related organelles, and it is also possible that they provide a food source for the symbiontids [7].

In contrast with the dominant free-living euglenids, there is an assemblage of eight heterotrophic genera *incertae sedis* (*Michajlowastasia, Parastasiella, Dinemula, Paradinemula, Mononema, Ovicola, Naupliicola* and *Embryocola*) that exhibit obligate parasitic lifestyles. Although their host range is rather narrow, encompassing exclusively freshwater, free-living copepods (specifically the eggs, larvae and digestive tracts of adults), their geographical range spans across the eutrophic freshwater bodies of all continents and nearly all climate zones, covering the range of their host group. Unfortunately, the phylogeny of these eight genera remains unresolved, as virtually all studies of the parasitic euglenids, however extensive, were carried out in the pre-sequencing era [356]. Occasionally, other photosynthetic (*Euglenamorpha*) and heterotrophic (*Heteronema*) euglenids have been identified in the gastrointestinal tracts of a very wide range of vertebrate and invertebrate animal hosts; however, it remains disputed whether they are symbionts or parasites [357,358]. Several species of euglenophytes (mainly genus *Colacium*) have been recognized as parasites or, more likely, epibionts of zooplankton [359–363], while others (e.g. *Euglena mutabilis, Trachelomonas hispida*) have also been identified in the traps of carnivorous plants, such as *Genlisea* [364] or *Utricularia* [365]. Whether euglenophytes are prey, accidental inhabitants, or a part of the specialized community of the carnivorous plants' traps, remains unresolved.

As most aquatic microbial eukaryotes, euglenids and symbiontids are considered cosmopolitan. Our understanding of their distribution is hampered by the limited number of environmental sequencing projects focused on those groups. Despite clear microscopical evidence of phagotrophic euglenids in sediments and phototrophic euglenids in ponds, they are suspiciously rare in environmental sequencing datasets [166,366–368]. It was suggested that euglenids' 18S rRNA is divergent and often longer than the typical eukaryotic one [369,370], and the universal primers are not working efficiently for euglenids. Environmental sequences might be, however, obtained with specific primers designed for a certain group [371]. The same as for diplonemids, the V9 region of 18S rRNA seems to be a more suitable metabarcoding marker, and phototrophic euglenids have been surveyed in the environmental sequences from the TARA Oceans dataset and OSD dataset [339]. Although euglenophytes are overall quite rare in the marine plankton, their distribution is quite broad in the global ocean, with preference for the coastal upwelling zones, where the nutrient availability is higher than in other parts of the ocean [372]. Symbiontids have been investigated in several environmental 18S rRNA-based surveys in different geographical regions, suggesting their cosmopolitan distribution [166,373–376].

### 4.2. Taxonomy

Class Euglenida Bütschli, 1884 emend. Simpson, 1997 (tree F).

**Tree F.**
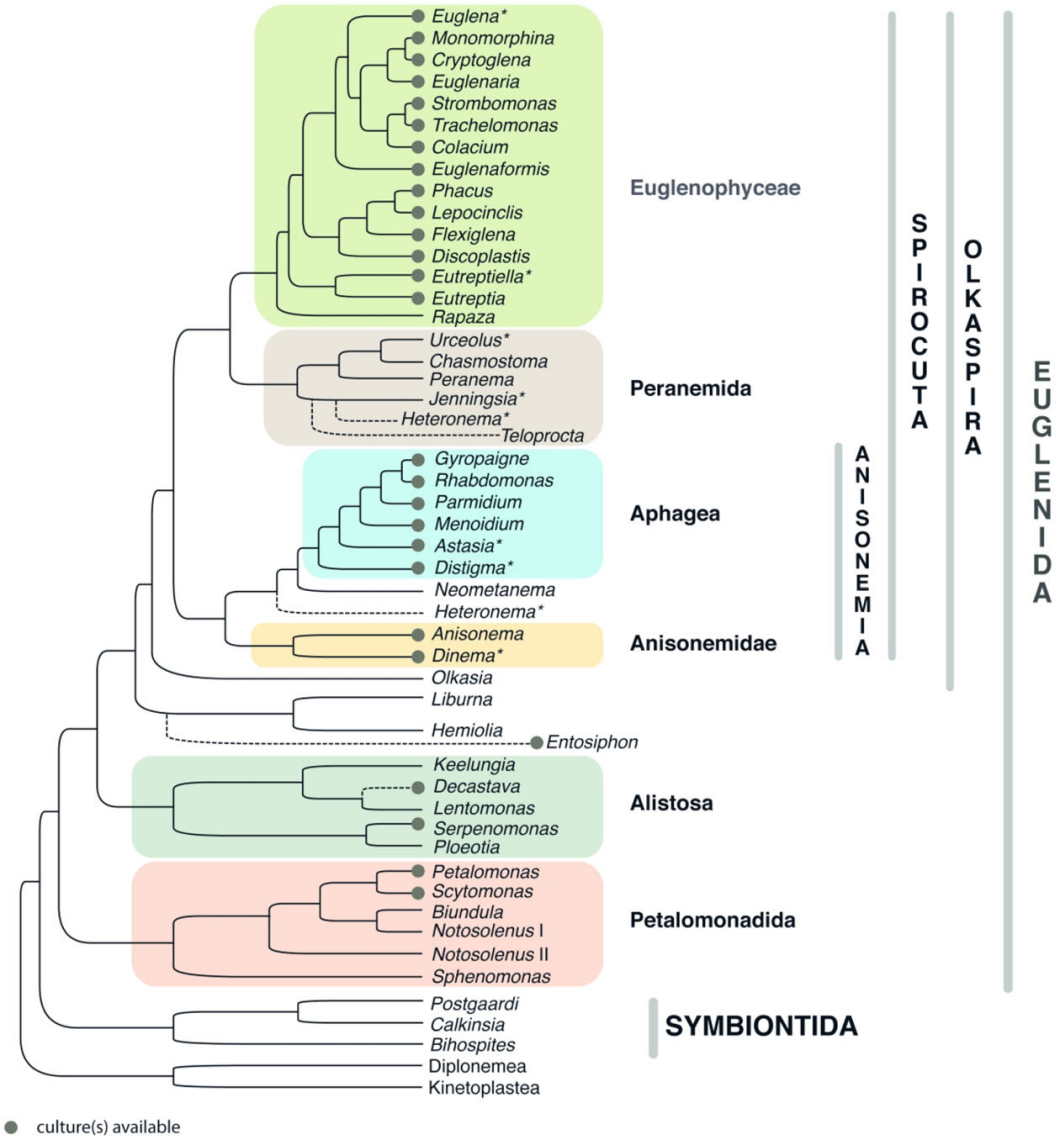
Euglenids and symbiontids. A tree summarizing multiple phylogenetic reconstructions, primarily based on 18S rRNA gene sequences, genes encoded by plastid genomes (Euglenophyceae), and a set of nuclear protein-coding genes retrieved from transcriptomes. Polyphyletic genera are marked with an asterisk (*). Lineages with highly unstable position are marked with a dotted line. Possible paraphyly of clades has been further described in the section on Euglenids taxonomy.

Euglenids’ synapomorphy is a pellicle build of proteinaceous strips beneath the plasma membrane. The strips can be fused together in certain genera; otherwise, the organisms are capable of characteristic ‘euglenoid motion’ also known as ‘metaboly’. Another characteristic feature, shared with Kinetoplastida, is the presence of flagella inserted at the base of the flagellar pocket, and the flagella are conspicuously thickened due to the presence of paraxonemal (paraflagellar) rods. Additionally, the main storage polymer of most euglenids is paramylon, a distinctive β-1,3-glucan.

Note: Historically, species belonging to class Euglenida were described according to the rules of the ICZN or the ICN due to the presence of photosynthetic organisms within this ancestrally non-photosynthetic group. This has created some confusion, as several taxa bear two different, equally valid names. Moreover, many clades are unstable and assigning a rank and a name to them would be unproductive, since these names are likely to become obsolete very soon. The taxonomy proposed here is a consensus between a strictly organized taxonomy for stable clades and informal nomenclature. Additionally, Euglenophyceae and their subordinate taxa are treated as algae and classified according to ICN (with alternative names consistent with ICZN provided in notes), while all other subordinate taxa of Euglenida are classified according to ICZN (with alternative names consistent with ICN provided in notes).

• Clade Olkaspira Lax and Simpson, 2020. This robustly supported monophyletic clade includes organisms with pellicle composed of S-shaped proteinaceous strips with overhangs, and chisel-shaped feeding apparatus (if the apparatus is present; see below) [8]. Flexible cells belong to the subordinate clade Spirocuta; rigid cells belong to the subordinate genus *Olkasia*:
  ▪ Genus *Olkasia* Lax, Lee, Eglit and Simpson, 2019. Rigid, flattened, biflagellate cells with 10 pellicle strips; consists of only one species (*O. polycarbonata*), formerly classified as *Ploeotia* [377].
  Type species: *Olkasia polycarbonata* Lax, Lee, Eglit and Simpson, 2019 (plate F, 93). No culture available; several sequences of SSU rDNA and transcriptomes obtained from single-cell isolates.

○ Clade Spirocuta Cavalier-Smith, 2016. The monophyletic group encompassing all flexible euglenids including phototrophs (Euglenophyceae), primary osmotrophs (Aphagea) and various phagotrophs [378]. The synapomorphy of this group is the capability for ‘metaboly’ also known as ‘euglenoid motion’.
Note: The other name used for this assemblage is Helicales [170,330,379,380].

▪ Clade Euglenophyceae Schoenichen, 1925 emend. Marin and Melkonian, 2003. The monophyletic group [170,381–383] comprising the basal monotypic genus *Rapaza* [355]. A predominantly photosynthetic group with plastids derived from secondary endosymbiosis with green alga, some species secondarily osmotrophic, most species with photosensory eyespot.
Note: The other name used for this group is Euglenea Bütschli, 1884 emend. Busse and Preisfeld, 2002; however, as pointed out by Cavalier-Smith in the work cited above, this name is shared with a beetle genus.
  ○ Order Euglenales Leedale, 1967 emend. Marin and Melkonian, 2003. Cells with one emergent flagellum and one vestigial within the cell; feeding by phototrophy or secondarily by osmotrophy, mostly freshwater [3]; 18S rRNA gene has C in the first position of the Helix 7/8 spacer [381]; introns highly abundant in the plastid genome (always more than 28 and usually more than 51); intron maturase *mat1/ycf13* always present in the plastid genome, with *mat2, mat5* or both of them usually present [384].
    • Family Euglenaceae Dujardin, 1841 emend. Kim *et al*., 2010. Solitary or colonial, mostly free-living, but some inhabit the digestive tracts of animals; usually possess one emergent flagellum and one non-emergent; some may possess mineralized external shells (lorica); size, number and presence of pyrenoids in chloroplasts varies with the species [3]; ribosomal operon may be present in the plastid genome in one copy or more, but never as two identical inverted repeats [384].
      Note: valid name under ICZN is Euglenidae Dujardin, 1841.
      ▪ Genus *Colacium* Ehrenberg, 1834. Solitary or colonial cells with envelopes that also form stalks for surface attachment [385]; often sessile (epizoic; attached to copepods) [386]; ribosomal operon present in the plastid genome in one full and one incomplete copy with the same orientation [387].
        Type species: *Colacium vesiculosum* Ehrenberg, 1834. Type species and other species available in the culture collections; multiple 18S rRNA sequences and a full plastid genome sequence of the type species are available.
      ▪ Genus *Cryptoglena* Ehrenberg, 1831. Rigid, solitary, laterally compressed cells with a longitudinal furrow and one or two chloroplasts (plate F, 77); 18S rRNA gene has AT base pair in the third position of the Helix 40 [381].
        Type species: *Cryptoglena pigra* Ehrenberg, 1832. Type species and other species available in the culture collections; multiple 18S rRNA sequences and a full plastid genome sequence of *C. skujae* (non-type species) are available.
      ▪ Genus *Euglena* Ehrenberg, 1830. Solitary cells with very visible metaboly; chloroplasts with pyrenoids (plate F, 85); at least two species (*E. longa, E. quartana*) secondarily non-photosynthetic, feeding by osmotrophy; 18S rRNA gene has T (rarely A or C) in the seventh position of the Helix 47/33 spacer [381]; ribosomal operon present in the plastid genome in one copy or in consecutive, tandemly repeated copies [384]; mitochondrial genome consists of seven linear chromosomes and encodes seven proteins which constitute components of complexes I, III and IV of the respiratory chain [27].
        Type species: *Cercaria viridis* O.F. Müller, 1786 (= *Euglena viridis*).
        Note: the genus *Euglena* is polyphyletic—on the phylogenetic trees *Euglena archaeplastidiata* and *Euglena velata* do not group with the main clade of *Euglena* [382,383]. Type species and other species available in culture collections, multiple sequences of 18S rDNA available; nuclear genome, plastid genome and mitochondrial genome of *E. gracilis* (non-type species), as well as multiple plastid genomes of other species, are available.
      ▪ Genus *Euglenaformis* Bennett and Triemer, 2014. Cryptic genus, morphologically undistinguishable from *Euglena*, but phylogenetically basal to all Euglenaceae; ribosomal operon present in the plastid genome in one copy [388]. *Euglenaformis* is a monospecific genus.
        Type species: *Euglena proxima* Dangeard, 1902 (= *Euglenaformis proxima*) (plate F, 84). Available in culture collections; 18S rRNA and a full plastid genome sequence of the type species are available.
      ▪ Genus *Euglenaria* Karnkowska, Linton and Kwiatowski, 2010. Solitary, metabolic cells with parietal, lobed chloroplasts with single pyrenoids and bilateral paramylon caps; distinguished from the genus *Euglena* by its distant phylogenetic position, sister to *Monomorphina* [389]; ribosomal operon present in the plastid genome in one copy [390].
        Type species: *Euglena caudata* Hübner, 1886 (= *Euglenaria caudata*). Type species and other species available in culture collections; 18S rDNA of multiple species and a full plastid genome sequence of *Ea. anabaena* (non-type species) are available.
      ▪ Genus *Monomorphina* Mereschkovsky, 1877. Rigid or slightly metabolic cells with pellicle-formed tail, 2–4 large paramylon plates and one or few large, spherical chloroplasts [381,391] (plate F, 81); 18S rRNA gene has T in the third position of the terminal loop of the Helix 27 [381]; ribosomal operon present in the plastid genome in one copy [390].
        Type species: *Euglena pyrum* Ehrenberg, 1832 (= *Monomorphina pyrum*). Type species and other species available in culture collections; 18S rRNA of multiple species and two full plastid genome sequences (*M. aenigmatica* and *M. parapyrum*; non-type species) are available*.*
      ▪ Genus *Strombomonas* Deflandre, 1930. Cells of variable shape and size with discoid or flat chloroplasts with pyrenoids and smooth lorica without collar [392]; ribosomal operon present in the plastid genome in one full and one incomplete copy with opposite orientation [387].
        Type species: *Trachelomonas hispida* var. *verrucosa* E. Daday, 1905 (= *Strombomonas verrucosa*). Type species and other species available in culture collections; 18S rRNA of multiple species and a full plastid genome sequence of *S. acuminata* (non-type species) are available.
      ▪ Genus *Trachelomonas* Ehrenberg, 1834. Cells of variable shape and size, with ornamented lorica with collar (plate F, 80); some species osmotrophic [381]; ribosomal operon present in the plastid genome in one copy [390].
        Type species: *Microglena volvocina* Ehrenberg, 1831 (= *Trachelomonas volvocina*). Type species and other species available in culture collections; 18S rRNA of multiple species and a full plastid genome sequence of the type species are available.
    • Family Phacaceae Kim, Triemer and Shin 2010. Solitary, free-living, with large paramylon grains and numerous small, discoid chloroplasts without pyrenoids.
      Note: valid name under ICZN is Phacidae Kim, Triemer and Shin 2010.
      ▪ Genus *Discoplastis* Triemer, 2006. Cells capable of metaboly, with a sharp, colourless tail [393,394]; two ribosomal operon-containing inverted repeats present in the plastid genome [384].
        Type species: *Euglena spathirhyncha* Skuja, 1948 (= *Discoplastis spathirhyncha*) (plate F, 83). Type species and other species available in culture collections; 18S rRNA of multiple species and a full plastid genome sequence of the type species are available.
      ▪ Genus *Flexiglena* Zakryś and Łukomska, 2020. Highly metabolic cells with numerous small paramylon grains and a distinct large, single grain located near the stigma [394].
        Type species: *Euglena variabilis* Klebs, 1883 (= *Flexiglena variabilis*). Currently not available in culture collections, but deposition of the type species in a culture collection is in progress; 18S rRNA of multiple species available.
      ▪ Genus *Lepocinclis* Perty, 1849. Rigid, unflattened cells with ring-shaped paramylon grains (plate F, 78); some (*L. cyclidiopsis*) secondarily non-photosynthetic, feeding by osmotrophy; 18S rRNA gene has GC base pair in the sixth position from the end of the Helix 12 and T in the second position of the Helix 23/27 spacer [381]; two ribosomal operon-containing inverted repeats present in the plastid genome [384].
        Type species: *Lepocinclis globulus* Perty, 1849. Type species and other species available in culture collections; 18S rRNA and full plastid genome sequences of multiple non-type species are available.
      ▪ Genus *Phacus* Dujardin, 1841. Rigid, laterally or triangularly compressed cells with ring-shaped paramylon grains (plate F, 79); some (*P. ocellatus*) secondarily non-photosynthetic, feeding by osmotrophy [381]; ribosomal operon present in one copy in the plastid genome [384].
        Type species: *Euglena longicauda* Ehrenberg, 1830 (= *Phacus longicauda*). Type species and other species available in culture collections; 18S rRNA and full plastid genome sequences of multiple non-type species are available.
  ○ Order Eutreptiales Leedale, 1967 emend. Marin and Melkonian, 2003. Solitary, free-living cells with two or four flagella of equal or unequal length, capable of metaboly [3]. Predominantly marine. Introns are present in their plastid genomes, but not abundant (usually fewer than 28 and never more than 51); intron maturase *mat1/ycf13* always present in the plastid genome, but *mat2* and *mat5* absent [384].
    • Family Eutreptiaceae Hollande, 1942. With the same definition as the order.
      Note: valid name under ICZN is Eutreptiidae Hollande, 1942.
      ▪ Genus *Eutreptia* Perty, 1852. Two emergent flagella of almost equal length [395]; ribosomal operon present in one copy in the plastid genome [384].
        Type species: *Eutreptia viridis* Perty, 1852. Type species and other species available in culture collections; 18S rRNA and a full plastid genome sequence of the type species are available.
      ▪ Genus *Eutreptiella* da Cunha, 1914. Two emergent flagella of notably unequal length [395] or four flagella composed of longer and shorter pairs [396] (plate F, 86); may possess epi- or endobiotic bacteria [397]; mostly psychrotolerant or psychrophilic [342,396]; 18S rRNA gene possesses a CA insertion after the second position in the loop of Helix 18 [381]; ribosomal operon present in two copies with opposite orientation in the plastid genome, but one copy may be split [170,381]. Type species: *Eutreptiella marina* da Cunha, 1914. Type species and other species available in culture collections; 18S rRNA sequences of multiple non-type species, two full plastid genome sequences (*Etl. gymnastica* and *Etl. pomquetensis*; non-type species) and a transcriptomic dataset of *Etl. gymnastica* (non-type species) are available.
        Note: the genus *Eutreptiella* is often paraphyletic in 18S phylogenies [170,381].
  ○ Order Rapazida Cavalier-Smith, 2016. Solitary, free-living cells with two flagella of unequal length, feeding by phagotrophy on microalgae such as *Tetraselmis*; marine, capable of metaboly [355].
    ▪ Family Rapazidae Cavalier-Smith, 2016. With the same definition as the order.
      ▪ Genus *Rapaza* Yamaguchi, 2012. With the same definition as the family.
        Type species: *Rapaza viridis* Yamaguchi, Yubuki and Leander, 2012 (plate F, 87); 18S rRNA sequence of the type species available.
  ○ Euglenophyceae *incertae sedis*—genera with unresolved position due to lack of molecular data, and therefore questionable status, are:
    ▪ Genus *Ascoglena* Stein, 1878. Small, solitary cells with lorica, often sessile (attached to filamentous algae) [392].
      Type species: *Ascoglena vaginicola* Stein, 1878.
    ▪ Genus *Euglenamorpha* Wenrich, 1924. Elongated, metabolic cells of highly variable size with 3–6 flagella of equal length; inhabit the intestinal tracts of *Rana* spp. tadpoles [357].
      Type species: *Euglenamorpha hegneri* Wenrich, 1924.
    ▪ Genus *Euglenopsis* Klebs, 1892. Sessile cells with four long flagella and transverse cell division, forming colonies of branched filaments attached to the surface [398].
      Type species: *Euglenopsis vorax* Klebs, 1892.
    ▪ Genus *Glenoclosterium* Carter, 1869. Spindle-shaped cells with visible eyespot and at least four longitudinally elongated chloroplasts, but without a notable emergent flagellum [399].
      Type species: *Glenoclosterium varians* Carter, 1869.
    ▪ Genus *Hegneria* Brumpt and Lavier, 1924. Elongated, colourless cells with six flagella of equal length; inhabits the intestinal tract of tadpoles [400].
      Type species: *Hegneria leptodactyli* Brumpt and Lavier, 1924.
    ▪ Genus *Klebsina* Silva, 1961. Sessile, loricate cells, inhabiting marine habitats; originally described as *Klebsiella* [392], renamed by Silva due to conflicting name with a bacterial genus [401].
      Type species: *Klebsiella alligata* Pascher, 1931 (= *Klebsina alligata*).
    ▪ Genus *Euglenocapsa* Steinecke, 1932. Small, oval-shaped, faintly coloured cells with multiple disc-shaped, pyrenoid-lacking chloroplasts adjacent to the cell wall and a single emergent flagellum of length up to three times the length of the cell [402].
      Type species: *Euglenocapsa ochracea* Steinecke, 1932.
      Note: No representative of the *incertae sedis* genera is available in culture collections.
  ▪ Clade Anisonemia Cavalier-Smith, 2016*.* The monophyletic group comprising predominantly flexible (metabolic), mostly biflagellate heterotrophs (phago- and osmotrophs), capable of skidding motility or gliding using posterior flagellum [378].
  ○ Order Anisonemida Cavalier-Smith, 2016. Feeding by phagotrophy, capable of gliding motility using posterior flagellum; paraphyletic with respect to Aphagea [8,378].
    • Family Anisonemidae Kent, 1880. With the same definition as the order.
      ▪ Genus *Anisonema* Dujardin, 1841. Weakly metabolic cells with two unequal flagella (posterior one is longer), occurs in brackish waters (plate F, 90); during mitosis, basal body duplication and replication occurs in the late stages [403]; many species/morphotypes distinguished [377].
        Type species: *Anisonema acinus* Dujardin, 1841. Not available in culture collections; 18S rRNA sequence of the type species and single-cell transcriptomes of multiple species are available.
      ▪ Genus *Dinema* Perty, 1852. Usually strongly metabolic cells (with few weakly metabolic or rigid species, e.g. *Dinema inaequale*) with a thick pellicle and two unequal flagella [300] (plate F, 92); paraphyletic with respect to *Anisonema* [378].
        Type species: *Dinema griseolum* Perty, 1852. 18S rRNA and single-cell transcriptomic data of several species available.
        Note: valid name under ICN is *Dinematomonas* Silva, 1960, since the name Dinema Perty, 1852 is a synonym of Dinema Lindley, 1826 (Plantae: Magnoliophyta).
    ○ Clade Aphagea Cavalier-Smith, 1993 emend. Busse and Preisfeld, 2002. Feeding by osmotrophy, without ingestion apparatus; monophyletic.
      ▪ Genus *Astasia* Dujardin, 1830. Cells without ingestion apparatus or stigma, with one emergent flagellum and visible metaboly; paraphyletic [170,404,405].
        Type species: *Astasia limpida* Dujardin, 1841. Multiple non-type species available in culture collections; 18S rRNA sequences of multiple non-type species are available.
      ▪ Genus *Distigma* Ehrenberg, 1831. Cells without ingestion apparatus, with two emergent flagella and intense metaboly; some (*D. proteus*) possess endobiotic bacteria; despite the name, no stigma present [170,379,404,405]; paraphyletic.
        Type species: *Distigma proteus* Ehrenberg, 1831. Type species and other species available in culture collections; 18S rRNA sequences of multiple species, including the type species, are available.
      ▪ Genus *Gyropaigne* Skuja, 1939. Rigid cells with prominent keels, fused pellicle and one emergent flagellum with hairs [404]; microtubule scroll present [404].
        Type species: *Gyropaigne kosmos* Skuja, 1939. *G. lefevrei* (non-type species) available in culture collection; 18S rRNA sequence of the same species is available.
        Note: this genus probably encompasses the organism described as *Helikotropis okteres* [406], as its existence as a separate entity is not supported by any distinguishing morphological feature or molecular data [392].
      ▪ Genus *Menoidium* Perty, 1852. Rigid, flattened and elongated cells with fused pellicle and one emergent flagellum without hairs [407]; microtubule scroll present [404] (plate F, 82).
        Type species: *Menoidium pellucidum* Perty, 1852. Multiple non-type species available in culture collection; 18S rRNA sequences of multiple species, including the type species, are available.
      ▪ Genus *Parmidium* Christen, 1962. Rigid cells with deep indentations, fused pellicle and one emergent flagellum without hairs [407]; microtubule scroll present [404].
        Type species: *Parmidium circulare* Christen, 1962. Type species and other species available in culture collection; 18S rRNA sequences of multiple species, including the type species, are available.
      ▪ Genus *Rhabdomonas* Fresenius, 1858. Rigid cells with fused pellicle and one emergent flagellum; microtubule scroll present; a rather disputable, paraphyletic genus with no distinct synapomorphy, encompassing multiple species sharing different traits of other genera [404].
        Type species: *Rhabdomonas incurva* Fresenius, 1858. Type species and other species available in culture collections; 18S rRNA sequences of multiple species, including the type species, are available.
    ○ Order Peranemida Cavalier-Smith, 1993. Uniflagellate or biflagellate cells, capable of gliding motility using anterior (or single) flagellum
      Note: this clade is resolved on some trees as polyphyletic, encompassing four clades (themselves monophyletic), scattered across the tree of Spirocuta [378]. Only a recent multigene phylogeny resolves them as a monophyletic sister clade to Euglenophyceae, but with very weak support [8]. Regardless of the phylogenetic uncertainties, these organisms have been informally referred to as ‘peranemids’ due to their common morphological traits.
      ▪ Genus *Peranema* Dujardin, 1841. Biflagellate, highly metabolic cells with longer and thicker anterior flagellum and protruding feeding apparatus, capable of cutting into other cells and sucking in its contents (plate F, 89); can and will attempt to feed on anything, including bacteria, yeast, microalgae, ink, raw starch and other euglenids [408]; very rapid response to light by rhodopsin-mediated phototaxis [409]; in recent phylogeny resolved as monophyletic [378].
        Type species: *Peranema globulosum* Dujardin, 1841. Non-type species (*P. trichophorum*) available in an educational resources repository (Carolina Biological Supply Co.), but not in culture collections; 18S rRNA sequence and single-cell transcriptomes of *P. trichophorum* (non-type species) are available.
        Note: valid name under ICN is *Pseudoperanema* Christen, 1962, since *Peranema* Dujardin, 1841 is synonym of *Peranema* D. Don 1825 (Plants: Polypodiopsida)
      ▪ Genus *Chasmostoma* Massart, 1920. Uniflagellate, metabolic cells with a pronounced flagellar cavity [378,410].
        Type species: *Chasmostoma nieuportense* Massart, 1920. Not available in culture collections; a single-cell transcriptome of the type species is available.
      ▪ Genus *Jenningsia* Schaeffer, 1918. Uniflagellate, metabolic cells [410] (plate F, 88); polyphyletic, currently comprising two separate monophyletic clades: one (*Jenningsia fusiforme*) branching at the base of Euglenophyceae, and another branching at the base of all non-peranemid Spirocuta [378]; in a recent multigene phylogeny, still resolved as polyphyletic, but both clades are placed within the monophyletic Peranemida [8].
        Type species: *Jenningsia diatomophaga* Schaeffer, 1918. Not available in culture collections; 18S rRNA sequences and single-cell transcriptomes of multiple non-type species strains are available.
      ▪ Genus *Teloprocta* Cavalier-Smith, 2016*.* Cylindrical or spindle-shaped cells with two long flagella (ventral and dorsal) and 28 pellicle strips; consists of species formerly classified as *Heteronema,* e.g. *Teloprocta scaphurum* [411].
        Type species: *Heteronema scaphurum* Skuja, 1934 (= *Teloprocta scaphurum*). Not available in culture collections; 18S rRNA sequence of the type species available.
      ▪ Genus *Urceolus* Mereschkovsky, 1877. Highly metabolic, sack-shaped cells with one emergent flagellum and a flattened anterior collar [300,378]; polyphyletic genus, with two strains (ABLN1 and WBF1) branching separately, with weak support, from the otherwise monophyletic sister clade to *Teloprocta* [378]; resolved as monophyletic in a recent multigene phylogeny; however, the two divergent strains mentioned above were not included in the analysis [8].
        Type species: *Urceolus alenizinii* Mereschkovsky, 1879. Not available in culture collections; 18S rRNA sequences and single-cell transcriptomes of multiple non-type species strains are available.
    ▪ Genera unassigned to any subordinate taxa within Spirocuta:
      ▪ Genus *Neometanema* Lee and Simpson, 2014. Biflagellate, flattened cells with two equally long flagella, visible feeding apparatus, weak metaboly and 22 helical pellicle strips; distinguished from *Heteronema/Anisonema* by skidding motility involving the use of both flagella; a taxonomic replacement for *Metanema* [331,378].
        Type species: *Neometanema parovale* Lee and Simpson, 2014. Not available in culture collections; single-cell transcriptomes of multiple species, including the type species, are available.
      ▪ Genus *Heteronema* Dujardin, 1841. Biflagellate cells, capable of metaboly and gliding movement using the longer and thicker anterior flagellum, freshwater (plate F, 91).
        Type species: *Heteronema marina* Dujardin, 1841. Not available in culture collections; 18S rRNA sequences and single-cell transcriptomes of multiple non-type species strains are available.
        Note: *Heteronema* is a highly disputable genus, comprising species with varied morphological features [331,411]; recent phylogeny resolves this taxon as polyphyletic, with *Heteronema globuliferum* branching within Peranemida, and *Heteronema vittatum* (monophyletic) branching within Anisonemida, neither of which is the type species [378].
• Clade Alistosa Lax *et al*., 2020. Oval-shaped, biflagellate cells, usually with 10–12 pellicle strips with keels; using the longer posterior flagellum for gliding motility; monophyletic [8,377].
  ▪ Genus *Ploeotia* Dujardin, 1841. Rigid, biflagellate cells (posterior one trailing against the substrate) with non-protrusible ingestion apparatus [300,412].
    Type species: *Ploeotia vitrea* Dujardin, 1841. Not available in culture; 18S rRNA sequences of multiple strains and two single-cell transcriptomes, both including the type species, are available.
  ▪ Genus *Serpenomonas* Triemer, 1986. Small, slightly flattened cells with two flagella (posterior one is longer) and a non-retractive feeding apparatus, inhabiting salt marshes [413].
    Type species: *Serpenomonas costata* Triemer, 1986. Type species available in culture; 18S rRNA sequences of multiple strains of the type species are available.
    Note: due to having a stable sister relationship, the genera *Ploeotia* and *Serpenomonas* are often referred to by a collective name Ploeotiidae [377,378].
  ▪ Genus *Keelungia* Chan, 2013. Biflagellate, very small cells [414] with 10 flat pellicle strips [377].
    Type species: *Keelungia pulex* Chan and Moestrup, 2013. Not available in culture; 18S rRNA sequences of multiple species, including the type species, are available.
  ▪ Genus *Lentomonas* Farmer and Triemer, 1994. Rigid biflagellate cells with thicker and longer posterior flagellum, and straight, longitudinal pellicle strips [415], out of which seven dorsal strips are prominent, while three ventral strips are flat [377] (plate F, 96).
    Type species: *Entosiphon applanatum* Preisig, 1979 (= *Lentomonas applanatum*). Not available in culture; 18S rRNA sequences of multiple non-type species are available.
  ▪ Genus *Decastava* Cavalier-Smith, 2016. Long anterior flagellum and short posterior flagellum; 10 longitudinal pellicle strips [411].
    Type species: *Decastava edaphica* Cavalier-Smith and Vickerman, 2016. Type species available in culture; 18S rRNA sequences of multiple species, including type species, are available.
  ○ Order Petalomonadida Cavalier-Smith, 1993. Uniflagellate or biflagellate cells, using the longer, anterior flagellum (or the single flagellum) for gliding motility; recent phylogeny resolves Petalomonadida as monophyletic with strong support [378].
    ▪ Genus *Petalomonas* Stein, 1859. Rigid, flattened cells with one emergent gliding flagellum, mostly freshwater [300].
      Type species: *Cyclidium abcissum* Dujardin 1841 (= *Petalomonas abcissa*). *P. cantuscygni* (non-type species) available in culture collection; 18S rRNA sequences and single-cell transcriptomes of multiple non-type species are available.
    ▪ Genus *Scytomonas* Stein, 1878. Possesses five pellicle strips, a single flagellum and centriole, feeds when sessile [411].
      Type species: *Scytomonas pusilla* Stein, 1878. *Sc. saepesendens* (non-type species) available in culture collection; 18S rRNA sequence of the same species is available.
    ▪ Genus *Notosolenus* Stokes, 1884. Rigid, flattened cells with long anterior and short posterior flagellum [300] (plate F, 94).
      Type species: *Solenotus apocamptus* Stokes, 1884 (= *Notosolenus apocamptus*). Not available in culture collections; 18S rRNA sequences of multiple non-type species strains and a single-cell transcriptome of *N. urceolatus* (non-type species) are available.
    ▪ Genus *Biundula* Cavalier-Smith, 2016. Possesses a single emergent flagellum, consists of four species formerly classified as *Petalomonas*, e.g. *Biundula sphagnophila, Biundula sulcata,* distinguished by pellicle structure (2–8 smooth undulations on dorsal and ventral surface, continuous pellicle without sutures between strips) [411].
      Type species: *Petalomonas sphagnophila* Christen, 1962 (= *Biundula sphagnophila*). Not available in culture collections; 18S rRNA sequence of the type species available.
    ▪ Genus *Sphenomonas* Stein, 1878. Small, rigid, biflagellate cells with a large hyaline inclusion [378] (plate F, 95); no phagotrophy observed, probably osmotrophic [416].
      Type species: *Sphenomonas quadrangularis* Stein, 1878. Not available in culture collections; a single-cell transcriptome of the type species is available.
• Genera unassigned to any subordinate taxa within Euglenida:
  ▪ Genus *Hemiolia* Lax, Lee, Eglit and Simpson, 2019. Oblong, moderately flattened cells with very long (over three times cell length) posterior flagellum, hardly notable pellicle strips and feeding apparatus not visible in light microscopy; consists of only one species (*H. trepidum*), formerly classified as *Anisonema* [377].
    Type species: *Anisonema trepidum* J. Larsen, 1987 (= *Hemiolia trepidum*). Not available in culture; 18S rRNA sequences of multiple strains of the type species are available.
  ▪ Genus *Liburna* Lax, Lee, Eglit and Simpson, 2019. Rigid, oblong cells with very long (about three times cell length), hooked posterior flagellum, hardly noticeable pellicle strips and feeding apparatus not visible in light microscopy; consists of only one species (*L. glaciale*), formerly classified as *Anisonema* [377].
    Type species: *Anisonema glaciale* Larsen and Patterson, 1990 (= *Liburna glaciale*). Not available in culture; 18S rRNA sequences of multiple strains of the type species and two single-cell transcriptomic datasets are available.
  ▪ Genus *Entosiphon* Stein, 1878. Cells with protrusible ingestion apparatus and 12 pellicle strips [377].
    Type species: *Anisonema sulcatum* Dujardin, 1841 (= *Entosiphon sulcatum*). Two species, including the type species, available in culture; 18S rRNA sequences of multiple species, including the type species, are avaiable.
    Note: the position of this genus is neither strongly supported nor stable, as depending on methods and datasets used for phylogeny, it may either branch off together with *Hemiolia* and *Liburna*, or form a separate branch in various positions among ‘rigid’ euglenids (i.e. Euglenida excluding Olkaspira). Therefore, despite the abundance of molecular data, *Entosiphon* cannot be classified as a member of any major group within Euglenida, and should be regarded as orphan genus among phagotrophic euglenids [8,377,378].
• Euglenida *incertae sedis*—15 genera with unresolved position due to lack of molecular data, and therefore questionable status, are:
  ▪ Genus *Atraktomonas* Christen, 1962. Possesses a single emergent flagellum, closely related to *Petalomonas* [331,417].
    Type species: *Atraktomonas laevis* Christen, 1962.
  ▪ Genus *Calycimonas* Christen, 1959. Non-metabolic cells, possesses a single emergent flagellum [417,418].
    Type species: *Calycimonas physaloides* Christen, 1959.
  ▪ Genus *Dolium* Larsen and Patterson, 1990. Rigid, sessile cells with one emergent flagellum [300]; generic name shared with an animal genus—*Dolium* Lamarck, 1801 (Mollusca: Gastropoda).
    Type species: *Dolium sedentarium* Larsen and Patterson, 1990.
  ▪ Genus *Dylakosoma* Skuja, 1964. Distinguished from *Petalomonas* due to the presence of epibiotic bacteria [419].
    Type species: *Dylakosoma pelophilum* Skuja, 1964.
  ▪ Genus *Peranemopsis* Lackey, 1940. Uniflagellate, metabolic, wedge-shaped cells, with only one rod in the feeding apparatus and no eyespot [183].
    Type species: *Peranemopsis striata* Lackey, 1940.
  ▪ Genus *Tropidoscyphus* Stein, 1878. Slightly plastic cells with eight strips and two unequal flagella, but both are described as anterior [420].
    Type species: *Tropidoscyphus octocostatus* Stein, 1878.
  ▪ Genus *Michajlowastasia* Krell and Shabalin, 2008. Organisms with two-stage life cycle: a free-living reproductive phase, and a parasitic feeding phase, taking place in the intestines or other body cavities of copepods and ending with formation of cyst-like structures. In free-living stage, cells are indistinguishable from genus *Astasia*; in parasitic stage, cells lose the emergent flagellum, become larger in size and enriched with paramylon grains [356].
    Type species: *Astasia cyclopis* Michajłow, 1956 (= *Michajlowastasia cyclopis*).
    Note: this genus had been originally described by Michajłow under the name *Parastasia* in order to distinguish the assemblage of parasitic forms from the exclusively free-living *Astasia* spp. That name, however, was recognized as invalid due to its homonymity with an earlier described beetle genus *Parastasia* Westwood, 1841 (Coleoptera: Scarabaeidae), and subsequently renamed by Krell & Shabalin [356,421].
  ▪ Genus *Parastasiella* Michajłow, 1965. Organisms with two-stage life cycle, similar to *Michajlowastasia*, but the parasitic stage involves copepod eggs and larvae (nauplii) as hosts instead of adults. Cells are among the smallest of all known euglenids, reaching a maximum length of 5 μm, and form heterogenous paramylon grains of different size [356].
    Type species: *Astasiella velox* Michajłow, 1965 (= *Parastasiella velox*).
  ▪ Genus *Dinemula* Michajłow, 1965. Organisms with two-stage life cycle (see *Parastasiella*). Spindle-shape cells with two unequal flagella; the anterior flagellum is the longer one and is formed earlier (during parasitic stage), while the posterior one is formed during the free-living stage [356].
    Type species: *Dinemula celer* Michajłow, 1965.
  ▪ Genus *Paradinemula* Monchenko, 1967. Organisms with two-stage life cycle (see *Parastasiella*). Morphologically similar to *Dinemula*, but more oval-shaped, with longer anterior flagellum, a stiff, laterally protruding flagellum turned to the back, and a large translucent nucleus [356].
    Type species: *Paradinemula polonica* Monchenko, 1967.
  ▪ Genus *Mononema* Michajłow, 1967. Organisms with two-stage life cycle (see *Parastasiella*). Similar to *Paradinemula*, but with a single emergent flagellum, protruding from a swelling in the anterior part of the cell and directed towards the back of the cell [356].
    Type species: *Mononema reptans* Michajłow, 1967.
  ▪ Genus *Ovicola* Michajłow, 1965. Organisms with two-stage life cycle, similar to *Parastasiella*, but reproduction occurs in parasitic stage within copepod eggs, with the free-living stage's role limited only to invasion of new hosts. Egg-shaped, uniflagellate cells with a thick, arched flagellum which makes rowing movements only with its distal part. In free-living stage, each cell contains only one large paramylon grain [356].
    Type species: *Ovicola abyssinicus* Michajłow, 1965.
  ▪ Genus *Naupliicola* Michajłow, 1965. Organisms with two-stage life cycle, similar to *Ovicola,* but reproduction occurs in body cavities of copepod nauplii instead of eggs. Morphologically similar to *Ovicola*, but with multiple paramylon grains in free-living stage [356].
    Type species: *Naupliicola necans* Michajłow, 1965.
  ▪ Genus *Embryocola* Michajłow, 1969. Organisms with two-stage life cycle, similar to *Naupliicola*. Morphologically similar to *Naupliicola*, but develops specifically inside the eyes of copepod nauplii in the parasitic stage of its life cycle [356].
    Type species: *Embryocola ocelli* Michajłow, 1969.
  ▪ Genus *Copromonas* Dobell, 1908. Rigid, pyriform, colourless cells with one long emergent flagellum and clearly visible cytopharynx, feeding by phagotrophy; isolated from intestines of frogs (*Rana temporaria*) and toads (*Bufo vulgaris*); described as resembling *Petalomonas* and *Scytomonas,* but also observed to conjugate, which makes its affiliation to Euglenida disputable [422].
    Type species: *Copromonas subtilis* Dobell, 1908.
    Note: no representative of the *incertae sedis* genera is available in culture collections.

Class Symbiontida Yubuki, Edgcomb, Bernhard and Leander, 2009.

This group has two shared synapomorphies: a thick mantle of rod-shaped epibiotic bacteria covering almost the entire cell, and a layer of mitochondria-derived organelles with reduced or absent cristae located beneath the cell membrane. Despite superficially similar morphology (with the exception of the pellicle strips), their relationship with euglenids has not been fully resolved. Most commonly found in hypoxic zones of marine habitats.

▪ Genus *Bihospites* Breglia, Yubuki, Hoppenrath and Leander, 2010. Cells with a rudimentary pellicle, robust feeding rod and two morphotypes of epibiotic bacteria (large, rod-shaped ones arranged in longitudinal bands and small, spherical ones with extrusive apparatuses) [329,423].
  Type species: *Bihospites bacati* Breglia, Yubuki, Hoppenrath and Leander, 2016. Not available in culture; 18S rRNA sequence of the type species available.
▪ Genus *Calkinsia* Lackey, 1960. Cells with reduced feeding rod and without pellicle, but with elaborate extracellular matrix, orange in colour; only rod-shaped epibiotic bacteria present [423,424].
  Type species: *Calkinsia aureus* Lackey, 1960 (plate F, 97). Not available in culture; 18S rRNA sequence of the type species available.
▪ Genus *Postgaardi* Fenschel, Bernard, Esteban, Findlay, Hansen and Iversen, 1995. Cells with complex feeding apparatus with an oval-shaped gutter, covered by the anterior lip overlapping a reinforced ridge, but with less developed extracellular matrix [423,425]; phylogenetic position unresolved due to lack of molecular data.
  Type species: *Postgaardi mariagerensis* Fenschel, Bernard, Esteban, Findlay, Hansen and Iversen, 1995. Not available in culture; no sequence data available.

## 5. Viruses in Euglenozoa

Viruses are the most abundant and widespread life form on our planet. During several billion years of coevolution, viruses have developed specific mechanisms allowing them to infect virtually any cellular organism [426]. It was estimated that viruses lyse about 20% of oceanic protists daily and, thus, play a major role in regulating the Earth's biogeochemical cycle [427]. Euglenozoa are no exception to this rule, although viral diversity was thoroughly investigated only in kinetoplastids [428]. The reason for such discrepancy is obvious—this is by far the best-studied group, which includes several parasites of medical or economic importance [10]. There is no doubt that representatives of Euglenida, Diplonemea and Symbiontida can be infected by viruses, but this has not been verified experimentally.

Kinetoplastids possess DNA and RNA viruses. The only documented case of a DNA virus is the one infecting free-living *Bodo saltans* [429]. This *Bodo saltans* virus belongs to the family *Mimiviridae* and its genome of about 1.39 Mb is among the largest described genomes of giant viruses. The functional role this virus may play in the biology of bodonids remains to be elucidated, but the plethora of acquired adaptation traits (such as the mechanism to facilitate membrane fusion, interference competition, contracted translation machinery and inflated genome with numerous genome rearrangements) makes this virus an interesting model for future studies. Also of note is that the abundance of such nucleo-cytoplasmic large DNA viruses was estimated at 10^4^–10^5^ genomes ml^−1^ in the photic zone and 10^2^–10^3^ genomes ml^−1^ of water in the oxygen minimum zone of the World Ocean [430].

The situation with RNA viruses is more complex. Here, we will only discuss viruses with known genetic structure and will not cover older reports of the mere presence of virus-like particles (reviewed by Grybchuk *et al*. [428]). The best-studied cases are of *Leishmania* RNA viruses (*Leishmaniavirus* spp., LRVs of the family *Totiviridae*). Discovered in the late 1980s in representatives of the *Leishmania* subgenus *Viannia* [431], the very first *Leishmaniavirus* LRV1 was sequenced in the early 1990s [432], and its biological role was uncovered about 20 years later [433]. Its presence is linked to the increased metastatic potential, parasite burden, immune response in mouse models of leishmaniasis and frequent treatment failures [434,435]. Notably, Old World leishmanias *L.* (*Leishmania*) possess a phylogenetically related *Leishmaniavirus*, LRV2, which is widespread in isolates of *L. major* [436,437], but its role in the disease progression is unknown. The phylogenies of viruses and their respective hosts are mainly congruent, suggesting long-term coevolution [438]. In addition to *Leishmania*, representatives of *Leishmaniavirus* LRV3 and LRV4 have been documented in another group of trypanosomatids, *Blechomonas* spp. [439]. These viruses have probably been acquired from *Leishmania* during co-infections.

The most successful group of viruses infecting trypanosomatids are bunyaviruses (LBVs, proposed family *Leishbunyaviridae*). They infect multiple *Crithidia* and *Leptomonas* spp. [440,441], *Leishmania* (*Mundinia*) *martiniquensis* [442], and at least one isolate of *Phytomonas* sp. [441]. The wide distribution of these viruses can be explained by their encapsulated structure, which promotes easy dispersion in co-infections.

Narnaviruses (family *Narnaviridae*) were detected in *Leptomonas seymouri* and *Phytomonas serpens* [95,441,443]. The viral load in *L. seymouri* is extremely high, indicating that this virus may enhance *Leishmania* virulence in the case of *Leishmania donovani–Leptomonas seymouri* co-infections [444]. Other viruses are less widespread and, in many instances, appear to be restricted to a particular trypanosomatid host, as can be exemplified by Tombus-like viruses and *Ostravirus* in *Leptomonas pyrrhocoris* [441].

## 6. Endo- and ectosymbioses in Euglenozoa

### 6.1. Kinetoplastid endosymbionts

Reports on endosymbionts in kinetoplastids are rare, but a few cases have been described in detail. Endosymbiosis between a bacterium and a trypanosomatid host occurred independently at least twice in the evolutionary history of trypanosomatids, in which neither hosts nor bacteria are closely related. The members of subfamily Strigomonadinae engaged in endosymbiotic relationship with *Ca.* Kinetoplastibacterium spp., representatives of Alcaligenaceae family (Burkholderiales; β-proteobacteria). Three genera of Strigomonadinae—*Angomonas*, *Strigomonas* and *Kentomonas*—are considered to share an endosymbiont-bearing ancestor, in which the reductive evolution of the endosymbiont genome occurred prior to the radiation of the host genera [274,445]. Each member of Strigomonadinae carries a different *Kinetoplastibacterium* species, which co-evolved together with its host [285]. Another trypanosomatid, *Novymonas esmeraldas* (most closely related to *Leishmania*), established endosymbiosis with *Ca.* Pandoraea novymonadis, representing another family (Burkholderiaceae) of Burkholderiales [275].

The presence of endosymbionts in all these cases likely compensates for the inability of their hosts to synthesize certain metabolites, such as haem, nucleotides, and several amino acids and vitamins, which are provided by the bacteria [275,285,445]. Both *P. novymonadis* and *Kinetoplastibacterium* spp. feature genomes, which are strongly reduced compared to related free-living β-proteobacteria, nevertheless preserving genes necessary for nutritional provisioning of their hosts [195]. Both endosymbiotic associations are permanent with bacteria being transmitted vertically; however, the association between *Kinetoplastibacterium* and Strigomonadinae is considered more ancient than that of *N. esmeraldas* and *P. novymonadis* [89,195]. The latter partnership is characterized by the lack of stringent control over the number of bacteria, less extensive genome reduction, higher GC content, and the presence of TCA and amino acid synthesis pathways [89].

Outside of these two systems, the presence of endosymbiotic bacteria was reported from a free-living freshwater kinetoplastid *Bodo curvifilus* [446], while the recently studied endosymbionts of *Bodo saltans* have been assigned to *Paracaedibacter*, with possible role in defensive endosymbiosis [447]. Finally, the trypanosomatid *Phytomonas borealis* isolated from the midgut of spiked shieldbugs also harbours endosymbionts [448], although their taxonomic identity and function remain unknown.

### 6.2. Diplonemid endosymbionts

Diplonemids are known for establishing symbiosis with members of Holosporaceae and Rickettsiaceae families, which are exclusively parasitic/endosymbiotic lineages of α-proteobacteria [449]. At present, there are only two reports on endosymbionts in diplonemids: Holosporaceae bacteria inside two *Diplonema* species [450], and a hemistasiid *Namystinia karyoxenos* with a Rickettsiaceae endosymbiont [325]. While *D. aggregatum* and *N. karyoxenos* contain a single endosymbiont, *D. japonicum* harbours two species of bacteria from closely related genera, the genomes of which have been sequenced, assembled and analysed [451]. They are severely reduced with similar gene content retained, and lack all energy metabolism pathways, including glycolysis, pentose-P pathway, the TCA cycle and oxidative phosphorylation. Although complete synthesis pathways for amino acids or vitamins are absent, the nutritional role of the endosymbionts cannot be ruled out due to the large number of proteins without known functions. A large portion of their highly reduced genomes is dedicated to secreted proteins that are possibly involved in manipulation of the host metabolism. Similar to Holosporaceae and Rickettsiaceae in other protist hosts, the role of the diplonemid endosymbionts is not clear. However, it was hypothesized that due to the presence of various secretion/toxin systems, the endosymbionts might take part in Defence against bacterial pathogens [451].

### 6.3. Symbiontid symbionts

As suggested by their very name, the distinctive trait of the Symbiontida is their capability of forming permanent, probably obligatory symbiotic relationships [7,41]. Thus far, three distinctive kinds of epibiotic bacteria have been described to thrive on the surface of symbiontid cells: rod-shaped, sulfide-oxidizing ε-proteobacteria associated with symbiontid genera *Calkinsia* and *Bihospites* [41], extrusive apparatus-bearing cocci with strong resemblance to hypotrich ciliate-associated Verrucomicrobia, endemic to the genus *Bihospites* [329], and magnetotactic Deltaproteobacteria, associated with multiple unclassified environmental strains of symbiontids [452].

Although the role of magnetosome-bearing, but non-motile δ-proteobacterial symbionts is clearly to provide their hosts with magnetotaxis [452], the functions of other microorganisms in their relationships with symbiontids seem to be more complex. It has been suggested that the ε-proteobacterial symbionts detoxify the local surroundings to limit the inhibitory effect of sulfide on the cellular respiration of the symbiontids [41], while the Verrucomicrobia-like bacteria provide their hosts with a Defence mechanism against predators [329]. Additionally, the epibiotic bacteria can be used by their hosts as an auxiliary food source [329,452]. In exchange, the eukaryotic hosts' role is to provide their epibionts with various metabolites, such as hydrogen, as all symbiontids possess hydrogenosomes [7], and to serve as efficient means of transport along the oxycline in the deep-sea environment [41]. It remains uncertain, however, whether the metabolic coupling between the symbiontids and ε-proteobacteria is limited to the outward hydrogen flux, and, perhaps more importantly, if any metabolic exchange occurs between the Verrucomicrobia-like bacteria and their symbiontid hosts.

### 6.4. Euglenid symbionts

Curiously, the observable affinity of the symbiontids towards prokaryotic partners is, to some extent, shared by their postulated close relatives—the euglenids. In their case, the tight relationships with bacteria are not as widespread, as a majority of the described euglenid genera have never been observed to harbour any symbionts, but the diversity of these relationships may be substantially greater [397]. Unfortunately, our knowledge of the euglenid–bacteria associations remains superficial due to the fact that they have mostly been reported in the pre-genomics and transcriptomics era [419,453,454]. Nonetheless, it is undeniable that these relationships are widespread, as they involve both phagotrophic (genera *Petalomonas* and *Dylakosoma*; [419,455]) and photosynthetic euglenids (genera *Euglena, Phacus, Lepocinclis* and others; [454]), and of rather diverse nature, as euglenids have been observe to harbour bacteria within their cells [389,454], on their surface [419,456], or even both [397]. What is more, a single heterotrophic euglenid species (*Anisonema platysomum*) has been observed to harbour magnetosomes. It remains unclear whether this typically prokaryotic trait has been acquired by *Anisonema* from magnetotactic bacteria (similar to those associated with symbiontids), or evolved independently [457].

As indicated by the so far most elaborate studies of euglenid–bacteria associations, involving a phagotrophic euglenid *Petalomonas sphagnophila* and a strain of photosynthetic *Eutreptiella* sp., it is evident that euglenids are capable of harbouring multiple distinct bacterial symbionts simultaneously [397,455]. *Petalomonas sphagnophila* from Canadian peatlands has been observed to carry six different bacteria within its cells, namely two strains of Rickettsiales, one representative of Firmicutes, one γ-proteobacterium, one δ-proteobacterium and one enigmatic, pigmented prokaryote with unidentified affiliation [455]. Moreover, *Eutreptiella* from the Long Island Sound possesses epibionts classified as *Roseovarius, Oceanicaulis* (Alphaproteobacteria) and *Marinobacter* (Gammaproteobacteria), as well as an endobiotic representative of Rickettsiales, though phylogenetically distant from those associated with *P. sphagnophila* [397]. Unfortunately, except for the hypothesis that the epibiotic bacteria of *Eutreptiella* supply their host with vitamin B12, well supported by cultivation experiments and transcriptomic data, very little is known about the nature and purpose of the relationships between bacteria and the two aforementioned euglenid hosts [397,455]. In fact, it is uncertain whether these associations are symbiotic at all, especially considering that Rickettsiales are common intracellular parasites of a vast variety of eukaryotes [458].

## Note added in proof

After the acceptance of this paper for publication, a taxonomic description of two new kinetoplastid flagellates, marine *Papus ankaliazontas* and freshwater *Apiculatamorpha spiralis*, has been published [459]. These new taxa represent free-living representatives of the order Prokinetoplastida. The available 18S rRNA gene-based phylogeny does not allow estimating reliably the relationships of these flagellates with the previously characterized genera *Perkinsela* and *Ichthyobodo* due to low statistical supports [459]. Moreover, the inferred position of these new taxa contradicts with the well-supported topology of the recently published phylogenomic tree, which, however, does not include *Ichthyobodo* [30]. Thus, it is currently premature to classify the two new genera and they are considered as Prokinetoplastida *incertae sedis*. Below is a short characterization of these forms.

*P. ankaliazontas*: free-living, solitary, eukaryvorous; two unattached flagella, the anterior one with thin undulate mastigonemes; flagellar pocket connected to oblique groove; pronounced rostrum with apical cytostome, tubular cytopharynx supported by prismatic microtubular rod; trichocysts at the anterior end.

*A. spiralis*: free-living, solitary, eukaryvorous; two unattached flagella the anterior one with fine fiber layer; cell surface with spherical and lamellate scales; pronounced rostrum with apical cytostome, tubular cytopharynx supported by prismatic microtubular rod; trichocysts at the anterior end.

## Data accessibility

This article has no additional data.
